# Climate change threatens European conservation areas

**DOI:** 10.1111/j.1461-0248.2011.01610.x

**Published:** 2011-05

**Authors:** Miguel B Araújo, Diogo Alagador, Mar Cabeza, David Nogués-Bravo, Wilfried Thuiller

**Affiliations:** 1 Department of Biodiversity and Evolutionary Biology, National Museum of Natural SciencesCSIC, 28006, Madrid, Spain; 2 Rui Nabeiro Biodiversity Chair, CIBIO, University of Évora, Largo dos Colegiais7000 Évora, Portugal; 3 Forest Research Centre, Instituto Superior de Agronomia, Technical University of LisbonTapada da Ajuda, 1349-017 Lisbon, Portugal; 4 Department of Biological and Environmental Sciences, University of HelsinkiViikinkaari 1, 00014, Finland; 5 Centre for Macroecology, Evolution and Climate, University of CopenhagenUniversitetsparken 15, 2100, Denmark; 6 Laboratoire d'Ecologie Alpine, UMR-CNRS 5553, Université J. FourierBP 53, 38041 Grenoble Cedex 9, France

**Keywords:** Bioclimatic envelope models, climate change, conservation planning, gap analysis, Natura 2000 networks, protected areas

## Abstract

Europe has the world's most extensive network of conservation areas. Conservation areas are selected without taking into account the effects of climate change. How effectively would such areas conserve biodiversity under climate change? We assess the effectiveness of protected areas and the Natura 2000 network in conserving a large proportion of European plant and terrestrial vertebrate species under climate change. We found that by 2080, 58 ± 2.6% of the species would lose suitable climate in protected areas, whereas losses affected 63 ± 2.1% of the species of European concern occurring in Natura 2000 areas. Protected areas are expected to retain climatic suitability for species better than unprotected areas (*P*<0.001), but Natura 2000 areas retain climate suitability for species no better and sometimes less effectively than unprotected areas. The risk is high that ongoing efforts to conserve Europe's biodiversity are jeopardized by climate change. New policies are required to avert this risk.

## Introduction

With more than 100 000 sites across 54 countries, Europe has more protected areas than any other region in the World. In addition to protected areas (e.g. national parks, natural parks, nature reserves, protected landscapes, etc.), which are designated by individual countries, the European Union (EU) established the Natura 2000 network to ensure the long-term survival of its most valuable biodiversity. The Natura 2000 network includes two sets of areas: Special Protection Areas (SPAs) are classified under the Birds Directive to help conserve important sites for rare and vulnerable birds; Special Areas of Conservation (SACs) are classified under the Habitats Directive to conserve rare and vulnerable non-bird animals, plants and habitats. In the 27 countries that constitute the EU, the Natura 2000 contributes 27 661 sites covering 117 million hectares (17% of the EU surface) (E.C. 2009). Even though a variety of conservation areas exist in Europe, a common assumption is that successful management is achieved by protecting the valued features from the processes that threaten them. Yet, it is becoming evident that in addition to providing sustainable management of habitats and ecosystems, effective conservation strategies need to mitigate impacts of climate change. While actions to mitigate climate change and its impacts are being debated worldwide, biologists are finding evidence that across a wide range of taxonomic and functional groups species already are responding to climate change by altering their phenology, and geographical distributions (Hickling *et al.* 2006; Lenoir *et al.* 2008). Forecasts project even greater changes for the 21st century (e.g. Pereira *et al.* 2010; Thuiller *et al.* 2011). Some species might persist only if they can colonize new areas when their former ranges become unsuitable, while others might persist in areas where they retain portions of their current ranges (Hannah *et al.* 2007). The conservation of such ‘climate refugia’ is of critical importance for biodiversity, but are existing European conservation areas up to the task?

Assessments of climate change impacts on biodiversity have often used bioclimatic envelope models (BEMs). These models use associations between climate and species’ occurrences to enable projections of future altered potential distributions of species under climate change scenarios. Implementations of these methods as well as their uncertainties have been extensively reviewed (e.g. Araújo & Guisan 2006; Heikkinen *et al.* 2006; Elith & Leathwick 2009). Although specific uses of BEM have been criticized (e.g. Botkin *et al.* 2007), models including a thorough treatment of algorithmic uncertainties followed by careful interpretation of results remain a useful tool for forecasting continental-wide impacts of climate change on large numbers of species (Araújo *et al.* 2005b; Green *et al.* 2008; Kearney *et al.* 2010; Dobrowski *et al.* 2011). Essentially, ensembles of BEMs have been shown to project successfully the direction of range changes for most species under climate change, while being less effective in estimating the magnitude of such changes (Araújo *et al.* 2005b).

We assessed impacts of climate change on *c.* 75% of terrestrial vertebrates (*n*=585) of Europe and *c.* 10% of the European flora (*n*=1298). Even though the proportion of plant species available for modelling is smaller than for vertebrates, they provide a representative sample of the species’ responses to climate change as most life forms among European plants are included (Thuiller *et al.* 2005). Uncertainty was handled within an ensemble forecasting framework (Araújo & New 2007), implemented with 7 bioclimatic modelling techniques × 3 general circulation models × 4 emission scenarios, and were projected into a baseline period and three periods in the future (see Materials and Methods); 336 projections were obtained for each of 1883 species, yielding a total of 632 688 projections. To avoid errors arising from estimating losses of species across modelled areas of distribution where species do not occur, changes in climatic suitability scores for species were assessed only for cells that overlap with species’ actual ranges. We also explored uncertainties arising from the emergence of non-analogue climates in the 21st century (Fitzpatrick & Hargrove 2009).

Studies have occasionally examined impacts of climate change on conservation areas (Hannah *et al.* 2007; Coetzee *et al.* 2009; Hole *et al.* 2009), but they did not compare impacts inside and outside conservation areas. Thus, it is difficult to assess the relative contribution of conservation areas for protecting regional biodiversity in these studies. To overcome this limitation, we compared projected shifts inside and outside conserved areas and tested whether current protection offers any buffer to climate change. Two sets of analyses are performed. First, we assess the ability of nationally designated protected areas (hereafter termed protected areas, Figure S1) to retain suitable climate conditions for all species considered. Species gaining suitable climate conditions are termed ‘winners’, whereas species losing suitable climate conditions are termed ‘losers’. Secondly, we examine potential impacts of climate change on subsets of species of conservation concern. Specifically, we examine impacts on globally threatened species (vulnerable, endangered, critically endangered according to the World Conservation Union IUCN) in protected areas, and on species prioritized by the EU legislation (hereafter termed Bird & Habitat Directive species) and occurring in Natura 2000 sites (Figure S1). The protected areas’ assessment is performed for 38 European countries (the Council of Europe member states excluding Turkey, Russia and the former Soviet republics), whereas the Natura 2000 analysis is performed for the European Union (EU) 26 Countries (excluding Cyprus).

## Materials and Methods

### Species data

We modelled 1883 European species: 1298 plants (Jalas & Suominen 1972-1996); 136 mammals (Mitchell-Jones *et al.* 1999); 343 breeding birds (Hagemeijer & Blair 1997); 42 amphibians (Gasc *et al.* 1997); and 64 reptiles (Gasc *et al.* 1997). Species with less than 20 records and mammals or birds with life cycles that are strictly aquatic or marine were not modelled. Plant species included all European pteridophytes, a sample of spermatophytes comprising representatives of all gymnosperm families (Coniferales, Taxales and Gnetales) and a fraction of angiosperm dicotyledons (Salicales, Myricales, Juglandales, Fagales, Urticales, Proteales, Santales, Aristolochiales, Balanophorales, Polygonales, Centrospermae, and Ranales), but no monocotyledons. The original grid is based on the Atlas Florae Europaeae (AFE), with cell boundaries following the 50 km lines of the Universal Transverse Mercator (UTM) grid, except near the border of the six-degree UTM zones and at coasts. The vertebrate atlases use slightly different grid systems, including different rules to represent data on islands and coasts. Hence, vertebrate data were converted to the AFE grid system by identifying unique (though sometimes approximate) correspondence between cells in these grids (Williams *et al.* 2000). The European mapped area (2434 grid cells) excludes most of the eastern European countries (except for the Baltic States) where recording effort was both less uniform and less intensive.

### Climate data

A set of climate parameters were derived from data provided by the Climate Research Unit at the University of East Anglia (Mitchell *et al.* 2004). The data provide monthly values for 1901–2000 in a 10′ grid resolution (*c.* 16 × 16 km). Average monthly temperature and precipitation in grid cells covering the mapped area of Europe were used to calculate mean values of four different climate parameters for 1961–1991 (referred to as ‘baseline data’). Variables included mean temperature of the coldest month (°C), mean annual summed precipitation (mm), mean annual growing degree days (> 5 °C) and a moisture index calculated as the ratio of mean annual actual evapotranspiration over mean annual potential evapotranspiration. Choice of variables was made to reflect two primary properties of the climate (energy and water) that have known roles in imposing constraints upon species distributions as a result of widely shared physiological limitations (e.g. Whittaker *et al.* 2007). All data were developed at a spatial resolution of 10′ across 11° W–32° E longitude and 34° N–72° N latitude and then projected to the AFE 50 km grid using bilinear interpolation.

Climate projections were derived for 1991–2020 (referred to as 2020), 2021–2050 (2050) and 2051–2080 (2080) from three climate models (CGCM2, CSIRO2 and HadCM3) (Mitchell *et al.* 2004). The modelled climate anomalies were scaled based on four scenarios proposed by the IPCC (Nakicenovic *et al.* 2000). The A1FI scenario describes a globalized world under rapid economic growth and global population that peaks in mid-century and declines thereafter. Concentrations of CO_2_ increase from 380 ppm in 2000 to 800 ppm in 2080, and temperature rises by 3.6 K. The A2 scenario describes a heterogeneous world with regionally oriented economic development. Per capita economic growth and technological change are slower than in the other scenarios. Global concentrations of CO_2_ increase from 380 ppm in 2000 to 700 ppm in 2080, and temperature rises by 2.8 K. The B1 scenario describes a convergent world with global population that peaks in mid-century and declines thereafter, as in A1, but with a rapid change towards the introduction of clean and resource-efficient technology. Concentrations of CO_2_ increase from 380 ppm in 2000 to 520 ppm in 2080, and temperature rises by 1.8 K. The B2 scenario describes a world in which the emphasis is on local solutions to socioeconomic and environmental sustainability. It is a world with continuously increasing global population (at a rate lower than A2), intermediate levels of economic development, and less rapid and more diverse technological change than in B1 and A1 scenarios. Concentrations of CO_2_ increase from 380 ppm in 2000 to 550 ppm in 2080, and temperature rises by 2.1 K.

### Protected areas

Two conservation areas datasets were used (see Figure S1): The World Database of Protected Areas (http://www.wdpa.org) and the NATURA 2000 GIS (European Commission, Directorate-General Environment, personal communication). WDPA-UNEP contains point and polygon data for protected areas, whereas the NATURA 2000 GIS database contains polygon data alone. Only protected areas of IUCN categories I–VI were considered. WDPA-UNEP data (point and polygon layers) were downloaded and then clipped to the geographical area of Europe. Points overlapping with polygons and points with no information on area coverage were removed. For each data point with surface area information, a polygon (circle) of corresponding surface area was created, centred at the point coordinates. All WDPA-UNEP and NATURA 2000 layers were converted to Lambert Equal Area Azimutal 10/52 projection to match the biodiversity data. The ATEAM-project geographical window (http://www.pik-potsdam.de/ateam/) was used to obtain the percentage of each 10′ grid cell overlapping with conservation areas polygons (Figure S1). Computations were performed separately for NATURA 2000 areas and WDPA areas, with custom-made functions for GIS (available upon request to MC).

### Bioclimatic modelling

An ensemble of BEM was generated for each of 1883 species considered. The ensemble included projections with seven methods: generalized linear models (GLM), generalized additive models (GAM), boosted regression trees (BRT), classification tree analysis (CTA), artificial neural networks (ANN), flexible discriminant analysis (FDA), and surface range envelope (SRE). Models were calibrated for the baseline (1961–1991) using 80% random sample of the initial data and evaluated against the remaining 20% data, using the area under the curve (AUC) of the receiver operation characteristic (ROC) and the true skill statistic (TSS) (Liu *et al.* 2011). Projections were performed 10 times, each time selecting a different 80% random sample while verifying model accuracy against the remaining 20%. Verification or internal evaluation does not allow assessing the predictive performance of the models – independent evaluation data would be required for this purpose – but it provides a measure of internal consistency of the models. Here, the evaluation statistics were used to consider the possibility of exclusion of species on the basis of poor matching between predictions and observations. Here, species with AUC < 0.7 or TSS values < 0.3 would be removed from the analysis. However, it was found that no species needed to be removed in addition to the species removed for being mainly aquatic or having too small range sizes (Figure S2). For the final assessment, models were calibrated using 100% of the species distribution data as it has been shown that random removal of presence records adds a non-trivial amount of uncertainty in future projections (Araújo *et al.* 2009). Given the ensemble of projections obtained with the seven BEMs and the three climate models, we calculated a consensus for each period and scenario. The consensus was based on a weighted mean probability of occurrence per species and per grid cell, where weights are obtained from the TSS obtained on the evaluation data (Marmion *et al.* 2009). The range of uncertainties obtained with the seven modelling techniques was also calculated (Table S4). All models were run using default options of the BIOMOD package (Thuiller *et al.* 2009).

### Assessing climate change impacts on species

Assessments of climate change impacts on biodiversity typically start with measurements of changes in the size and position of bioclimatic envelopes. This procedure can be problematic if impacts of climate change are assessed for protected areas, because envelopes represent potential distributions of species and it is changes in the actual distributions of species that matter for conservation planning. Using the full bioclimatic envelopes to assess the impacts of climate change on protected areas would amount to estimating species losses from areas where they might not occur, thus undermining the usefulness of the assessment. To overcome this problem, we restricted calculations of changes in climatic suitability to grid cells where species occur at present time. To do so, we downscaled species atlas information (originally at 50 km grid cell size) to 10′ grid cells (Araújo *et al.* 2005a): if a species occurs in a 50 km grid cell, it was assumed to occur in each of the respective 10′ grid cells with suitable climate; otherwise, it was assumed absent. Our analysis measures the exposure of species distributions to climate changes but it does not account for species migrating into conservation areas. Essentially, this is analogous to making an assumption of no dispersal.

### Assessing impacts of climate change on conservation areas

#### Matching species distributions with conservation areas

Although the European grid cells used in the assessment are of near-equal size (10′ latitude and longitude), the area conserved in each one of them varies (Figure S1). For example, in Belgium the average size of protected areas is 170 hectares (ha), whereas in Portugal it is 13 430 ha. To account for variation in conservation areas coverage when assessing species’ conservation status in grid cells, we applied an index derived from a probabilistic estimation of the matching between species’ climate suitability and the proportion of the grid cells that is conserved (Alagador *et al.* 2011). Starting with the assumption that modelled climate suitability for species is uniformly distributed within grid cells, the matching of climate suitability with conserved (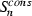) and non-conserved area (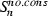), in a given grid cell, can be expressed for every species as: 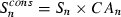
(1)
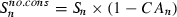
(2) where *S*_*n*_ is the suitability score of grid cell *n* for a given species and *CA*_*n*_ is the proportion of the grid cell *n* covered by conservation areas; both *S*_*n*_ and *CA*_*n*_ range from 0 to 1. Values for 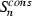
range from 0, for grid cells that are unsuitable for the species or with no areas conserved, to 1, for cells with climate suitability equal to 1 and for cells fully conserved. Values for 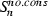 also range between 0, for grid cells where the climate is unsuitable for the species or where conservation areas fully cover the grid cell, to 1, for grid cells where climate suitability equals one and there are no areas conserved. This method avoids the multiple problems of using arbitrary thresholds for deciding whether grid cells are protected or not (Araújo 2004; Alagador *et al.* 2011).

For each one of the *T* combinations of timelines (baseline, 2020, 2050 and 2080) and emission scenario (A1FI, A2, B1, B2), we quantified the expected climate suitability *S* for each species within conservation areas (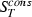) and outside conservation areas (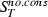) as: 
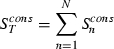
(3)
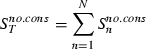
(4) where *N* is the number of grid cells in the analysis. For assessments focused on individual countries, *N* ranges from *N*=1, for Monaco and Liechtenstein, to *N*=2874, for Sweden. When the assessments are made for the whole of Europe, the geographic window varies because *N*=24 585 for European protected areas (i.e. countries associated with the Council of Europe) and *N*=20 871 for the NATURA 2000 (i.e. EU countries). Changes in 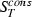
and 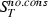 were calculated for each species with reference to the baseline period (*T*= baseline). Species projected to have increased climate suitability in conservation areas in each future time slice (*T*= future) were termed winners (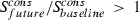), while species projected to have decreased climate suitability were termed losers (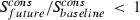). Numbers of winner and loser species were obtained for three geographic levels (European, country and grid cell). Standardized assessments of the proportion of winner and loser species at the country and grid-cell levels were obtained by dividing the number of winner and loser species in a particular time slice in the future by the total number of species present in the baseline period, respectively.

#### A null model for estimating the relative effectiveness of protected areas under climate change

We generated a null model to evaluate how European conservation areas (CA) perform in comparison to a random set of areas of equivalent total surface *R*. 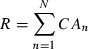
(5)

For protected areas, *R*=2078.74 and for NATURA 2000, *R*=2192.41. We developed a procedure to generate random sets of areas following the frequency distribution of conservation areas coverage in Europe, for protected areas and NATURA 2000. We undertook 1000 permutations of grid cells by randomly locating conservation area coverages across grid cells and keeping constant, for the first analysis, the coverage of protected areas and, for the second, NATURA 2000 sites. In each permutation, 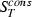
was assessed for each species, *T* combination of timelines (baseline, 2020, 2050 and 2080) and emission scenario (A1FI, A2, B1, B2). A null expected distribution of the proportion of winners and losers for each *T* combination was produced. The modelled proportion of losers and winners in conservation areas was then compared against the frequency distribution values from random trials at 95, 99 and 99.9 percentiles.

### Auxiliary analyses

McNemar chi-squared tests of marginal homogeneity were applied to analyse differences in the proportion of loser species in each climatic scenario and period considered (function *mcnemar.test* in R). We assessed the six possible scenario combinations (A1FI vs A2; A1FI vs B1; A1FI vs B2; A2 vs B1; A2 vs B2 and B1 vs B2) for all species in protected areas and for species of European concern in Natura 2000. As the number of Red-Listed species is small, the number of dissimilar occurrences in the contingency table (winner/loser or loser/winner) is less than 20 for these data. For this case, we used Wilcoxon signed ranked test for paired samples. Wilcoxon rank-sum tests were also used to compare range sizes of cold-adapted Bird & Habitat Directive species with warm-adapted Bird & Habitat Directive species.

## Results

**Figure 1:**
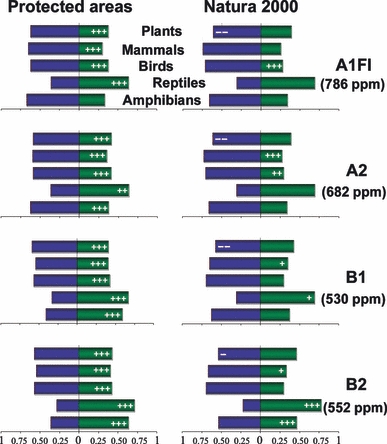
Proportion of species projected to gain (winners; green) or lose (losers; blue) climatic suitability in European conservation areas under four emission scenarios by 2080 (ppm are ‘part per million’ concentrations of CO_2_eq). Projections are provided for all modelled species in protected areas and for EU Bird & Habitat Directive species occurring in the Natura 2000. Conservation areas retaining more climatic suitability for species than expected in randomly selected unprotected areas are marked with +++ (*P*<0.001), ++ (*P* < 0.01), + (*P* < 0.05), whereas conservation areas retaining less climatic suitability for species than expected in randomly selected unprotected areas are marked with −− (*P* < 0.01) and − (0.05).

Most vertebrate and plant species (58 ± 2.6%; Median ± SD) are projected to lose suitable climate conditions within existing protected areas by 2080 (Fig. 1, full set of results in Table S1). Birds and mammals are projected to have greater proportions of loser than winner species in all scenarios, whereas amphibians are projected to have more losers than winners under A1FI and A2 and more winners under B1 and B2 scenarios (Fig. 1). Increases in climate suitability for species are expected for most reptiles in protected areas under all emission scenarios (67 ± 3.7%). This is unsurprising as ectothermic species are known to benefit from warming in temperate regions (Araújo *et al.* 2006), although local behaviour and population dynamics can alter, sometimes reverse, coarse projections from bioclimatic models (Sinervo *et al.* 2010). Amphibians are also ectotherms but they do not benefit from increases in aridity, which is the prediction for the southwest of Europe under the A1FI and A2 scenarios (Schroter *et al.* 2005). Projections also indicate that negative impacts of climate change are expected to be high among species of European conservation concern. Bird & Habitat Directive species (*n*=323) have higher proportions of plant and animal species losing climatic suitability in the Natura 2000 (63 ± 2.1%, Table S2) than species in protected areas. In fact, the Natura 2000 is less effective in retaining suitable climate for plant species than sets of randomly selected unprotected areas of the same total area (*P*<0.001 for A1FI, A2, B1; *P*<0.05 for B2). For half of the remaining combinations of taxonomic groups and scenarios, the Natura 2000 provides no better buffer against climate change than areas outside the network, with the exception of birds (*P*<0.001) (Fig. 1). In contrast, nationally designated protected areas are projected to retain climatic suitability for species better than randomly selected unprotected areas with the same total area (*P*<0.001). The one exception is amphibian species, under the A1FI scenario, where protected areas provide no better protection than randomly chosen unprotected areas. When threatened species are examined (*n*=53), protected areas retain climatic suitability no better than randomly selected unprotected areas for birds and reptiles (under the A1FI and A2 scenario), but they retain suitable ranges for the other taxa and climate scenarios well (*P*<0.05 or 0.01, see Table S1). Differences in changes of climate suitability between protected areas and Natura 2000 are partly related with topography. Most protected areas are in mountains (median altitude = 367.40 m) or rugged environments (median SD of altitude = 814.90). The Natura 2000 also prioritizes farmlands and these are located in lower (median altitude = 324.69) and flatter lands (median SD of altitude = 638.08). Notice, altitude and SD of altitude for the Natura 2000 were measured for the fraction of land that does not overlap with protected areas. Differences in altitude (Wilcoxon *W* = 13.4e6) and SD of altitude (*W* = 11.6e6) between protected and Natura 2000 areas are significantly different (*P* < 0.001). Because proportional range losses arising from climate change are usually more pronounced in flatlands than in rugged terrains (Peterson 2003; Loarie *et al.* 2009), the Natura 2000 is more vulnerable to climate change.

Losses of climatic suitability generally increase with greenhouse-gas emissions for IUCN Red-Listed species occurring in protected areas (*r*^2^=0.82, *P*<0.001), and similar relationship is recorded for the full set of species in protected areas and Bird & Habitat Directive species in the Natura 2000 (*r*^2^=0.71, *P*<0.001). In the worst-case scenario, with CO_2_ equivalent concentrations increasing from *c.* 380 ppm in 2000 to *c.* 800 ppm in 2080, and European temperatures rising by 3.6K ± 0.6 in 2080 (A1FI scenario), the proportion of loser species in protected areas exceeds 60% and is the greatest among all four emission scenarios (A1FI=62%, A2 = 58%, B1 = 57%, B2 = 55%; McNemar test χ^2^>37.3, *P*<0.001, Table S2). A similar trend is projected for the Red-listed species in protected areas and the Bird & Habitat Directive species in Natura 2000 (Table S2).

**Figure 2:**
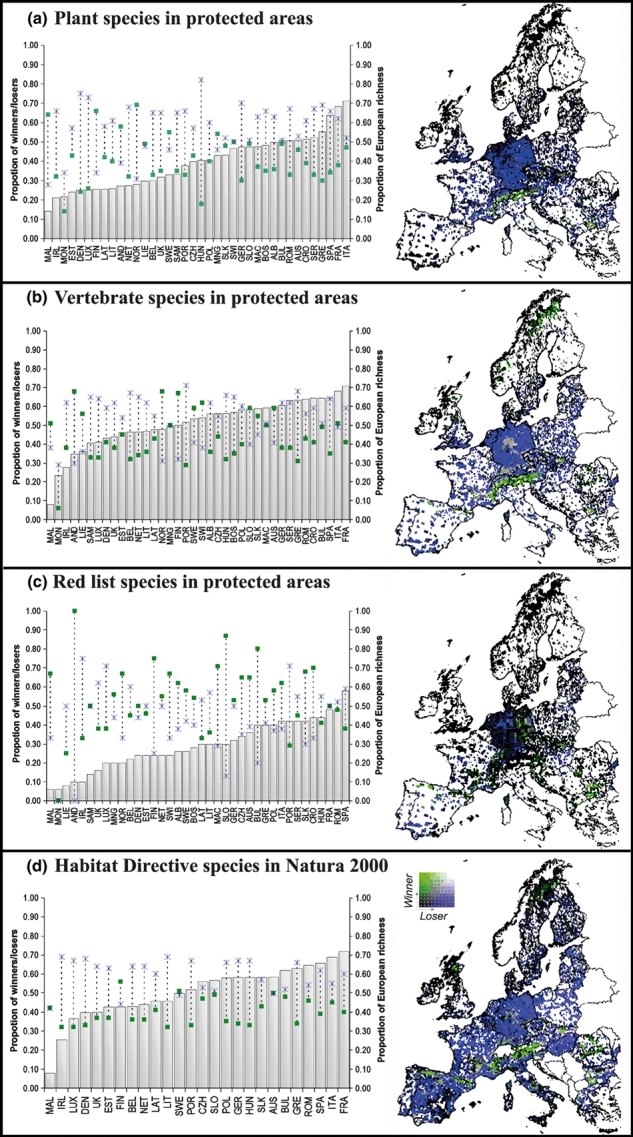
Geographical distribution of winners and losers. Left – The proportion of European species that occur in each individual country (bars, left axis) against the proportion of projected loser (blue asterisks, right axis) and winner species (green squares, right axis) as projected for 2080 with the A1FI scenario: (a) plant species occurring in protected areas; (b) vertebrate species occurring in protected areas; (c) IUCN Red data vertebrate and plant species occurring in protected areas (*n*=52); (d) Bird & Habitat directive vertebrate and plant species occurring in Natura 2000 sites (*n*=317). Notice that countries on the *x*-axis are ordered by the proportion of European species that occur in them. Right – Overlay between richness of species losing and winning suitable climate in conservation areas. Scores are divided into 10 equal-interval colour classes, where increasing intensities of blue represent increasing numbers of species losing suitable climate in conservation areas and increasing intensities of green represent increasing numbers of species winning suitable climate; shades of grey represent linearly covarying scores between winners and losers. All 10′ latitude and longitude cells with > 0% coverage with conservation areas are coloured. Regions with several small-sized conservation areas appear to have greater degree of protection but for the analyses, the percentage of grid-cell coverage by conservation areas was computed (Figure S1) and combined with modelled climatic suitabilities for each species. Country abbreviations are as follows: ALB – Albania; AND – Andorra; AUS – Austria, BEL – Belgium; BOS – Bosnia & Herzegovina; BUL – Bulgaria; CRO – Croatia; CZH – Czech Republic; DEN – Denmark; EST – Estonia; FIN – Finland; FRA – France; GER – Germany; GRE – Greece; HUN – Hungary; IRL – Ireland; ITA – Italy; LAT – Latvia; LIE – Liechtenstein; LIT – Lithuania; LUX – Luxembourg; MAC – Macedonia; MAL – Malta; MNG – Montenegro; MON – Monaco; NET – Netherlands; NOR – Norway; POL – Poland; POR – Portugal; ROM – Romania; SAM – San Marino; SER – Serbia; SLK – Slovakia; SLO – Slovenia; SPA – Spain; SWE – Sweden; SWI – Switzerland; UK – United Kingdom.

**Figure 3:**
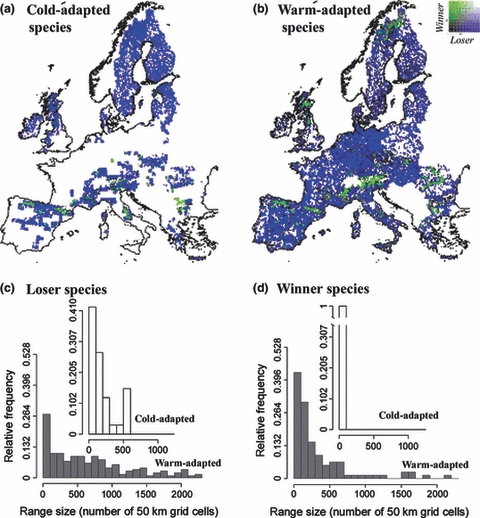
Vulnerability of cold-adapted vs. warm-adapted Bird & Habitat Directive species occurring in Natura 2000 areas: (a) overlay between richness of cold-adapted loser and winner species; (b) overlay between richness of warm-adapted loser and winner species. Scores on the maps are divided into 10 equal-interval colour classes, where increasing intensities of blue represent increasing numbers of loser species and increasing intensities of green represent increasing number of winner species; shades of grey represent linearly covarying scores between winners and losers; (c) Frequency distribution of the range sizes (number of grid cells occupied) of cold-adapted (empty bars) and warm-adapted loser species (dark bars) (*W* = 1530, *P* < 0.001); (d) Frequency distribution of range sizes of cold-adapted (empty bars) and warm-adapted winner species (dark bars) (*W* = 3, *P* = 0.100).

A geographical analysis reveals that loser species are predominant over winners across most protected and Natura 2000 areas. Higher proportion of winner species is projected in conservation areas of northern Scandinavia and Britain and in mountains such as the Alps, the Pyrenees and the Carpathians (Fig. 2). A country-by-country analysis reveals that all but two countries (Finland and Sweden) have more loser than winner species in Natura 2000 sites (Fig. 2, Table S3). The number of countries with a higher ratio between winners and losers is greater for protected areas than for Natura 2000, but the general tendency is for increased numbers of winners in the colder edges of Europe (Fig. 2). As expected, differences in thermal tolerance play a major role in accounting for the excesses of winners over losers in these areas. Many warm-tolerant species exist in high latitudes and altitudes and these will gain climatic suitability with climate warming, but the overwhelming majority of alpine and sub-arctic species of European concern (i.e. 97.2%) are projected to lose suitability (Fig. 3). Indeed, because such cold-adapted species have smaller ranges (range sizes at quartiles 25% = 35.5, 50% = 135.5, 75% = 260) than warm-adapted species (25% = 366; 50% = 1706; 75% = 2214), they are exposed to the double jeopardy of being rare and more negatively affected by climate change (Fig. 3).

**Figure 4:**
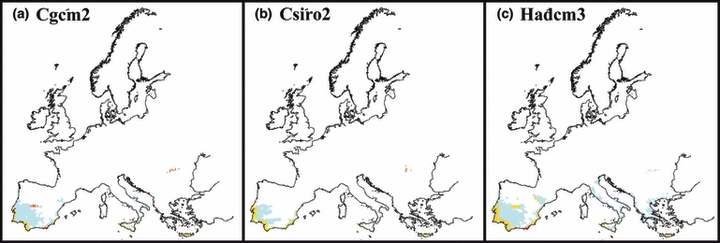
Distribution of non-analogue climates in 2080 under the A1FI emission scenario. For each variable (mean temperature of the coldest month, mean annual summed precipitation, mean annual growing degree days, and the ratio of mean annual actual evapotranspiration over mean annual potential evapotranspiration), non-analogue climates are defined as those exceeding the highest and lowest values recorded for the baseline. Colours indicate ‘richness’ of non-analogue climates, i.e. the summed occurrence of non-analogue climates for each variable, where increasing gradients of red indicate increased richness of non-analogue climates and white cells indicate absence of non-analogue climates.

Uncertainties from extrapolating beyond the climatic values used for calibration of the models are restricted to southern Europe, particularly the Iberian Peninsula (Fig. 4), thus not affecting the robustness of the results in most of Europe.

## Discussion

This study provides the most comprehensive analysis of climate change impacts and their uncertainties, on protected biodiversity anywhere in the world. However, not all uncertainties were accounted for. For example, population dynamics causing nonlinear responses to climate change (e.g. Keith *et al.* 2008; Anderson *et al.* 2009) were not modelled. The same can be said about interdependencies between species, some of which may have cascading effects and cause secondary extinctions when key species are removed (e.g. Ebenman & Jonsson 2005; Tylianakis *et al.* 2008). In fact, given the extent of the study region and the sheer number of species, simplifications are inevitable. The question is whether such simplifications enable useful projections under climate change. A number of studies have empirically demonstrated that carefully implemented bioclimatic models can recover the broad-scale direction of species range changes under climate change (Araújo *et al.* 2005b; Green *et al.* 2008; Rodríguez-Sánchez & Arroyo 2008; Kearney *et al.* 2010; Dobrowski *et al.* 2011) and others have demonstrated the usefulness of models for uncovering deep-time biological processes, such as extinction and speciation (e.g. Raxworthy *et al.* 2003; Nogués-Bravo *et al.* 2008; Carnaval *et al.* 2009). There are important uncertainties with regards to the magnitude of modelled range changes, as these are contingent on several unmeasured factors. However, evidence shows that models can recover the tendency of range increase or decrease with reasonable accuracy. Thus, one possible approach to limit uncertainty is to interpret model projections conservatively. By quantifying whether species are expected to win or lose climate suitability under climate change, we avoid making quantitative inferences about population parameters, such as changes in range, abundance or extinction risk, that are not explicitly modelled (e.g. Brook *et al.* 2009). Our ensemble forecasting strategy also surmounts familiar shortcomings of BEMs, particularly their potential to yield very different projections under future scenarios. Of particular relevance is the use of weighted-majority criterion to generate consensus among projections (Araújo *et al.* 2005b; Marmion *et al.* 2009) and for the restriction of analysis to areas with known records of occurrence for species, thus removing commission errors, and the use of a probabilistic-based approach to match conservation areas with climate suitability scores for species (Alagador *et al.* 2011). We also show that the risk of models extrapolating beyond the baseline climate is small, as non-analogue climates are restricted to southern Europe and impacts are projected for conservation areas throughout Europe. Despite methodological advances, any study using models needs to exercise caution when deriving conclusions with relevance for policy making. We argue that by examining changes in climatic suitability rather than making inferences about species range changes, we focus on what models truly deliver and substantially reduce uncertainties arising from simplification of complex ecological processes. Despite restricting our assessment to statistics of winners and losers, results have profound consequences for policy making.

Using analysis of 21st century climate change impacts on terrestrial vertebrate and plant species diversity in conservation areas, we demonstrate that climate change presents a challenge to the view that species distributions change relatively slowly unless they are directly affected by human activities. Specifically, we show that during the 21st century climate conditions are likely to become less suitable for species in European conservation areas. Nationally designated protected areas would preserve species better than unprotected areas, probably because they tend to occur in mountains, which act as climate refugia. Species in the Natura 2000 network are more vulnerable as more flatlands are included in the network and proportional range losses under climate impacts are greater there (Peterson 2003; Loarie *et al.* 2009). Our analysis does not provide quantitative estimates of extinction risk for species, because such estimates are beyond current data and modelling capabilities. However, future conservation efforts should be fully aware that the distribution of biodiversity, and species of concern, will be dramatically altered by climate change and that increased extinctions risk is one of the possible outcomes. Although reduction of greenhouse gas emissions would help mitigating climate impacts on biodiversity, conserving biodiversity will require approaches above and beyond those that are currently implemented in Europe. Such approaches might include the reclassification of existing conservation areas (Fuller *et al.* 2010) and the designation of new areas, as well as the implementation of mechanisms for integrated management of the countryside to facilitate movement of species between conservation areas. Making such moves implies a major paradigm shift in current conservation policies.

## SUPPORTING INFORMATION

**Figure S1** Proportion of 10' grid cells that are covered by protected areas (a) and Natura 2000 (b), and the respective frequency distribution values of the proportion of coverage of protected areas (c) and Natura 2000 (d).

**Figure S2** Predictive modelling accuracy for the five species groups. Each box represents the extreme of the lower whisker, the lower hinge, the median, the upper hinge and the extreme of the upper whisker for the true skill statistic (TSS), and the area under the curve (AUC). The boxes represent the metrics estimated during the cross-validation phase (on the testing data not used for calibrating the models) and across the seven different models retained.

**Table S1** (**a**) Numbers of species projected to gain (win) and lose (los) climatic suitability in protected areas (in parenthesis is the expected value outside protected areas), for different combinations of emission scenarios and time periods. Projections are provided for IUCN Red-Listed species (RL species), and for the complete pool of vertebrate and plant species considered (all). Protected areas retaining more loser species than 95% of a set of similar sized random selected areas are marked with ***(*P* < 0.001), **(*P* < 0.01), *(*P* < 0.05). (**b**) Numbers of species projected to gain (win) and lose (los) climatic suitability in Natura 2000 areas (in parenthesis is the expected value outside the Natura 2000), for different combinations of emission scenarios and time periods. Projections are provided for Habitat Directive species (HD species), and for the complete pool of vertebrate and plant species considered (all). Protected areas retaining more loser species than 95% of a set of similar sized random selected areas are marked with ***(*P* < 0.001), **(*P* < 0.01), *(*P* < 0.05).

**Table S2** Percentage of projected loser species. Values are provided for different combinations of suites of species, conservation areas, emission scenarios and years. Significant pairwise differences between means were assessed with McNemar tests for the complete pool of species in protected areas as well as for the Habitat Directive species in NATURA sites. Wilcoxon signed rank tests are provided for Red-listed species in protected areas. Letters a to e code the pairs of emission scenarios with proportions of loser species projected to be different with the following levels of significance ****P* < 0.001, ***P* < 0.01, **P* < 0.05. For the all-species in protected areas set, the A1FI scenario for 2020 differs from the A2 scenario (*a*, *P* < 0.01), the A2 scenario differs from the B1 scenario (*b*, *P* < 0.05), and the B1 scenario differs from the B2 scenario (*c*, *P* < 0.05).

**Table S3** (**a**) Number of species projected to gain (win) and lose (los) climatic suitability in protected areas (in parenthesis: outside protected areas), for the different countries, climatic storylines and analysed years. Projections are provided for Red-List species (RL species) and for all species (all). (**b**) Numbers of species projected to gain (win) and lose (los) climatic suitability in Natura 2000 areas (in parenthesis is expected value outside the Natura 2000), for different combinations of countries, emission scenarios and time periods. Projections are provided for Habitat Directive species (HD species), and for the complete pool of vertebrate and plant species considered (all).

**Table S4** (**a**) Variability in the numbers of species projected to gain (win) and lose (los) climatic suitability in European protected areas. Values are provided for different combinations of emission scenarios and time periods and using projections made with ensembles from seven bioclimatic modelling techniques and three general circulation models. Results for Red-List species (RL species) and for the complete pool of vertebrate and plant species considered (all). For each year, the winner and loser values in the first line refers to minimum and maximum values projected from the ensemble; second line refers to median value; and third line to standard deviation of values from the ensemble of forecasts. (**b**) Variability in the numbers of species projected to gain (win) and lose (los) climatic suitability in the Natura 2000 areas. Values are provided for different combinations of emission scenarios and time periods and using projections made with ensembles from seven bioclimatic modelling techniques and three general circulation models. Results for Habitat-directive species (HD species) and for the complete pool of vertebrate and plant species considered (all). For each year, the winner and loser values in the first line refers to minimum and maximum values projected from the ensemble; second line refers to median value; and third line to standard deviation of values from the ensemble of forecasts.

**Table S5** Species projected win and lose climate suitability in (in) and outside (out) protected areas (PA) and Natura 2000 areas (RN), for 2020, 2050 and 2080 under different emission scenarios. W stands for projected winners, whereas L stands for projected loser species.

As a service to our authors and readers, this journal provides supporting information supplied by the authors. Such materials are peer-reviewed and may be re-organized for online delivery, but are not copy-edited or typeset. Technical support issues arising from supporting information (other than missing files) should be addressed to the authors.
