# Supplementary material for: Climate change threatens European conservation areas
Source: Ecol Lett. 2011 May;14(5):484–92. doi: 10.1111/j.1461-0248.2011.01610.x (PMC3116148; doi:10.1111/j.1461-0248.2011.01610.x)
Supplement: Supplementary file 1 [file ele0014-0484-SD1.doc]

Supporting Online Material for

**Climate Change threatens European Conservation Areas**

Miguel B. Araújo*, Diogo Alagador, Mar Cabeza, Bruno Lafourcade, David Nogués-Bravo, & Wilfried Thuiller

*To whom correspondence should be addressed. E-mail: maraujo@mncn.csic.es

**This PDF file includes:**

**S1 (Figures)**

**S2 (Tables)**

Supporting Online Material for

**Climate Change threatens European Conservation Areas**

Miguel B. Araújo, Diogo Alagador, Mar Cabeza, Bruno Lafourcade, David Nogués-Bravo, & Wilfried Thuiller

**Contents**

S1. FIGURES

Figure S1, page 3

FIGURE S2, page 4

S2. Tables

table s1a, page 5

table s1b, page 7

table s2, page 9

TABLE S3a, page 10

table s3b, page 21

TABlE S4a, page 29

table s4b, page 30

# S1. Figures


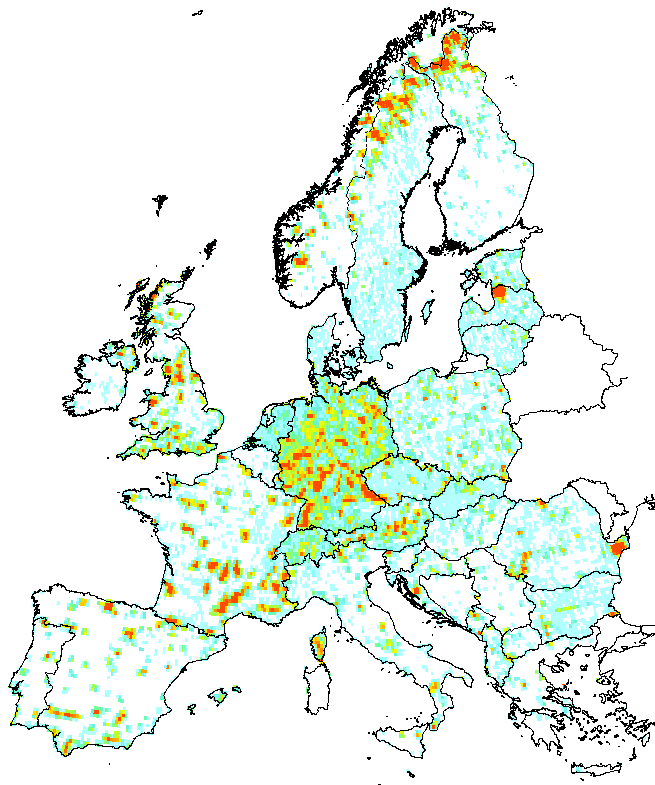

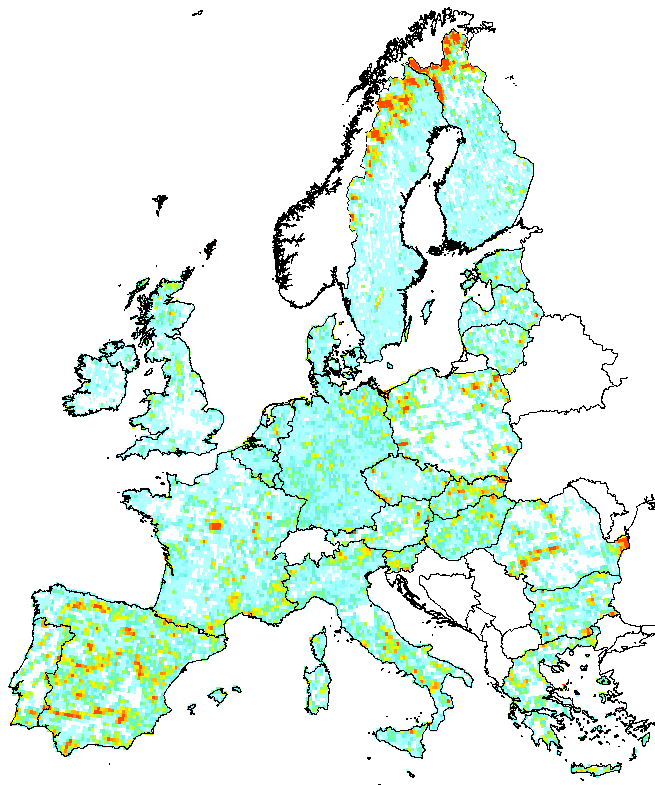


**A) B) B)**

**C) D) D)**


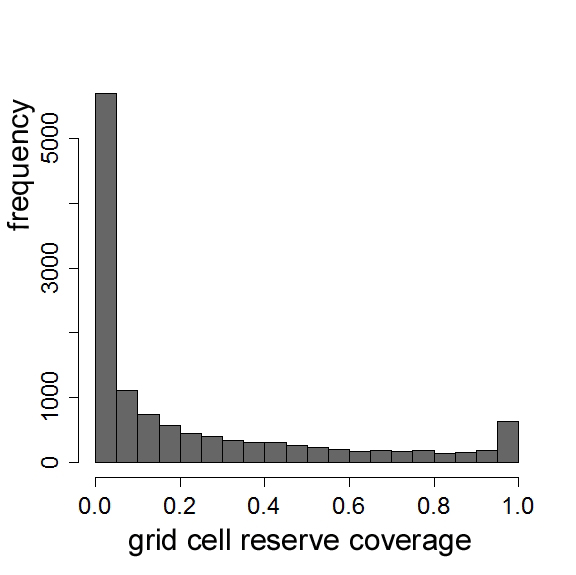

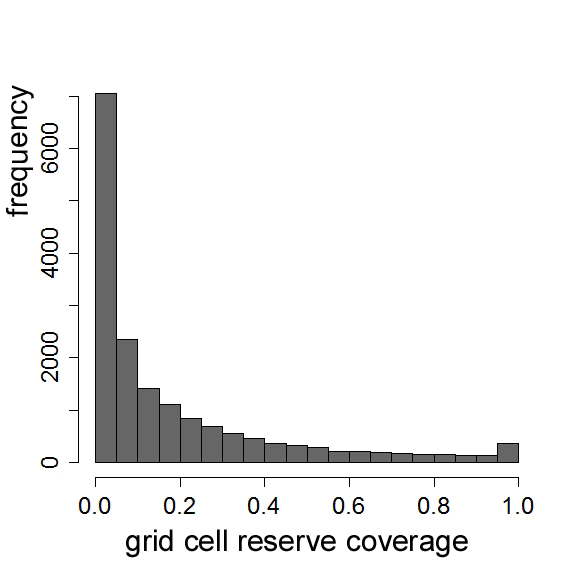


**Fig S1** – Proportion of 10’ grid cells that are covered by protected areas (A) and Natura 2000 (B), and the respective frequency distribution values of the proportion of coverage of protected areas (C) and Natura 2000 (D).


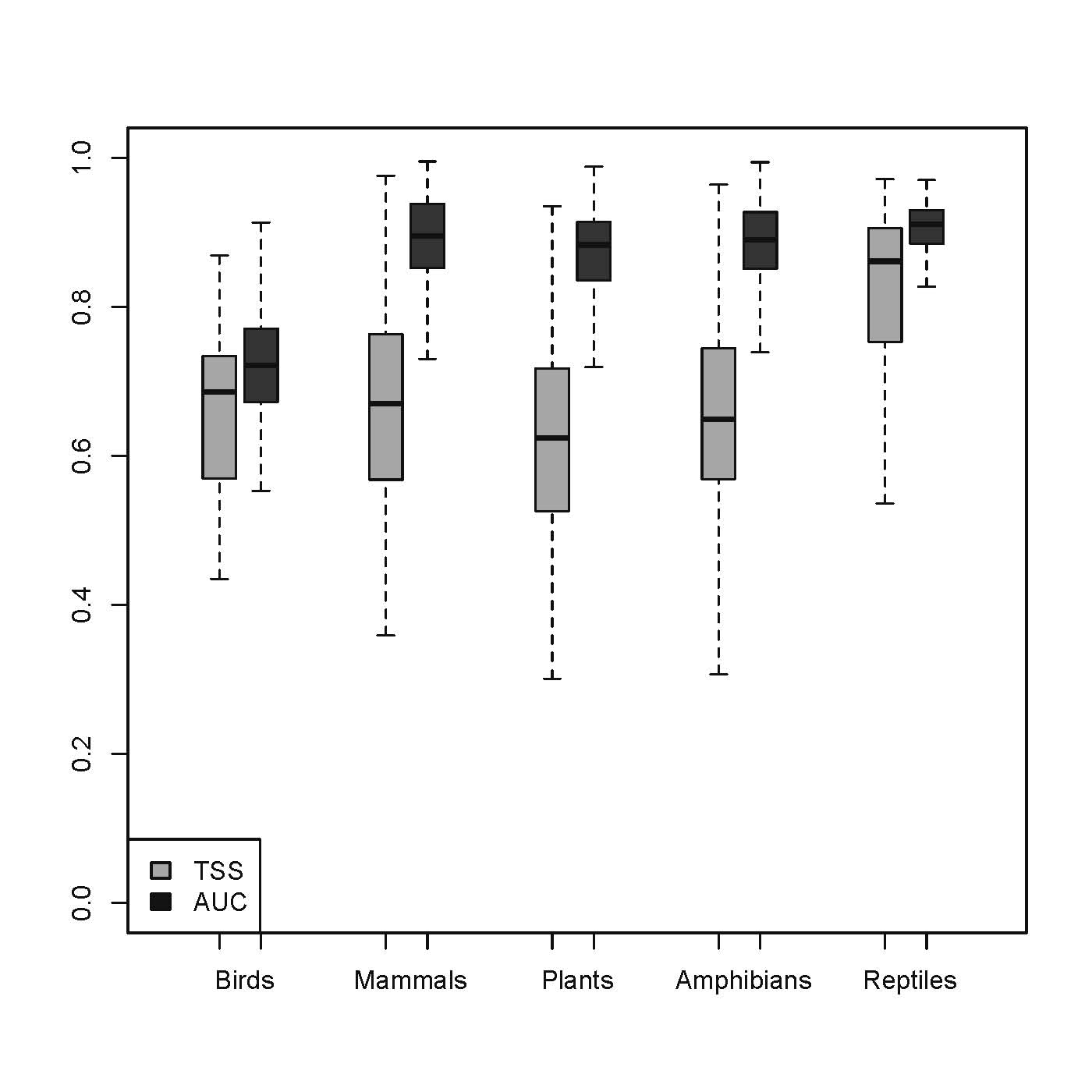


**Fig S2** –Predictive modelling accuracy for the five species groups. Each box represents the extreme of the lower whisker, the lower hinge, the median, the upper hinge and the extreme of the upper whisker for the True Skill Statistic (TSS), and the Area Under the Curve (AUC). The boxes represent the metrics estimated during the cross-validation phase (on the testing data not used for calibrating the models) and across the 7 different models retained.

# S2. Tables

**Table S1a -** Numbers of species projected to gain (win) and lose (los) climatic suitability in protected areas (in parenthesis is the expected value outside protected areas), for different combinations of emission scenarios and time periods. Projections are provided for IUCN Red-Listed species (RL species), and for the complete pool of vertebrate and plant species considered (all). Protected areas retaining more loser species than 95% of a set of similar sized random selected areas are marked with *** (*p*<0.001), ** (*p*<0.01), * (*p*<0.05).

|  | **A1FI** | | | | **A2** | | | | **B1** | | | | **B2** | | | |
| --- | --- | --- | --- | --- | --- | --- | --- | --- | --- | --- | --- | --- | --- | --- | --- | --- |
|  | **RL species** | | **all** | | **RL species** | | **all** | | **RL species** | | **all** | | **RL species** | | **all** | |
|  | **win** | **los** | **win** | **los** | **win** | **los** | **win** | **los** | **win** | **los** | **win** | **los** | **win** | **los** | **win** | **los** |
|  | |  |  |  |  |  |  |  |  |  |  |  |  |  |  |  |
| ***Amphibians*** | |  |  |  |  |  |  |  |  |  |  |  |  |  |  |  |
| **2020** | 8  (2) | 0  (6) | 32 (19) | 10 (23) | 8  (2) | 0  (6) | 32 (19) | 10 (23) | 7  (3) | 1  (5) | 32 (21) | 10 (21) | 8  (3) | 0  (5) | 34 (21) | 8  (21) |
|  | *** |  | *** |  | *** |  | *** |  | *** |  | *** |  | *** |  | *** |  |
| **2050** | 3  (2) | 5  (6) | 21 (18) | 21 (24) | 3  (2) | 5  (6) | 24 (19) | 18 (23) | 5  (2) | 3  (6) | 27 (19) | 15 (23) | 4  (2) | 4  (6) | 25 (19) | 17 (23) |
|  | * |  | *** |  | ** |  | *** |  | *** |  | *** |  | *** |  | *** |  |
| **2080** | 2  (2) | 6  (6) | 14 (14) | 28 (28) | 2  (2) | 6  (6) | 16 (14) | 26 (28) | 3  (2) | 5  (6) | 25 (13) | 17 (29) | 5  (2) | 3  (6) | 27 (14) | 15 (28) |
|  |  |  |  |  |  |  |  |  | * |  | *** |  | *** |  | *** |  |
| ***Reptiles*** | |  |  |  |  |  |  |  |  |  |  |  |  |  |  |  |
| **2020** | 5  (3) | 2  (4) | 46 (40) | 18 (24) | 5  (3) | 2  (4) | 48 (41) | 16 (23) | 5  (3) | 2  (4) | 45 (40) | 19 (24) | 5  (3) | 2  (4) | 49 (41) | 15 (23) |
|  | *** |  | *** |  | *** |  | *** |  | *** |  | *** |  | *** |  | *** |  |
| **2050** | 3  (2) | 4  (5) | 41 (36) | 23 (28) | 3  (2) | 4  (5) | 41 (34) | 23 (30) | 2  (2) | 5  (5) | 41 (37) | 23 (27) | 3  (2) | 4  (5) | 41 (36) | 23 (28) |
|  | *** |  | *** |  | *** |  | *** |  | *** |  | *** |  | *** |  | *** |  |
| **2080** | 3  (2) | 4  (5) | 41 (38) | 23 (26) | 3  (2) | 4  (5) | 41 (38) | 23 (26) | 4  (2) | 3  (5) | 43 (37) | 21 (27) | 6  (2) | 1  (5) | 46 (38) | 18 (26) |
|  | ** |  | *** |  | ** |  | *** |  | ** |  | *** |  | *** |  | *** |  |
|  | |  |  |  |  |  |  |  |  |  |  |  |  |  |  |  |
| ***Birds*** | |  |  |  |  |  |  |  |  |  |  |  |  |  |  |  |
| **2020** | 6  (4) | 8  (10) | 157 (132) | 185 (211) | 6  (4) | 8 (10) | 156 (135) | 186 (208) | 5  (3) | 9  (11) | 153 (128) | 189 (215) | 5  (3) | 9  (11) | 155 (130) | 187 (213) |
|  | *** |  | *** |  | *** |  | *** |  |  |  | ** |  |  |  | *** |  |
| **2050** | 5  (6) | 9  (8) | 154 (130) | 188 (213) | 6  (6) | 8  (8) | 157 (128) | 185 (215) | 5  (6) | 9  (8) | 158 (130) | 184 (213) | 4  (5) | 10  (9) | 154 (128) | 188 (215) |
|  |  | * | *** |  |  | * | *** |  |  | * | *** |  |  | * | *** |  |
| **2080** | 5  (4) | 9  (10) | 130 (101) | 212 (242) | 5  (4) | 9 (10) | 141 (111) | 201 (232) | 4  (3) | 10 (11) | 148 (120) | 194 (223) | 4  (4) | 10 (10) | 147 (118) | 195 (225) |
|  |  |  | *** |  |  |  | *** |  |  |  | *** |  |  |  | *** |  |
|  | |  |  |  |  |  |  |  |  |  |  |  |  |  |  |  |
| ***Mammals*** | |  |  |  |  |  |  |  |  |  |  |  |  |  |  |  |
| **2020** | 12 (10) | 9  (11) | 65 (51) | 70 (84) | 12 (11) | 9  (10) | 65 (52) | 70 (83) | 11 (11) | 10  (10) | 64 (55) | 71 (80) | 11 (11) | 10  (10) | 64 (57) | 71 (78) |
|  |  |  | *** |  |  |  | *** |  |  |  | *** |  |  |  | *** |  |
| **2050** | 11  (8) | 10  (13) | 56 (37) | 79 (98) | 11  (9) | 10  (12) | 57 (39) | 78 (96) | 11  (9) | 10  (12) | 59 (39) | 76 (96) | 11  (9) | 10  (12) | 58 (40) | 77 (95) |
|  | *** |  | *** |  | *** |  | *** |  | *** |  | *** |  | *** |  | *** |  |
| **2080** | 9  (7) | 12  (14) | 43 (35) | 92 (100) | 11  (7) | 10  (14) | 51 (36) | 84 (99) | 11  (7) | 10  (14) | 58 (37) | 77 (98) | 10  (7) | 11  (14) | 58 (37) | 77 (98) |
|  | *** |  | *** |  | *** |  | *** |  | *** |  | *** |  | ** |  | *** |  |
|  | |  |  |  |  |  |  |  |  |  |  |  |  |  |  |  |
| ***Plants*** | |  |  |  |  |  |  |  |  |  |  |  |  |  |  |  |
| **2020** | 1  (1) | 2  (2) | 663 (565) | 641 (740) | 1  (1) | 2  (2) | 678 (567) | 626 (738) | 1  (1) | 2  (2) | 668 (554) | 636 (751) | 1  (1) | 2  (2) | 676 (557) | 629 (748) |
|  |  |  | *** |  |  |  | *** |  |  |  | *** |  |  |  | *** |  |
| **2050** | 3  (2) | 0  (1) | 613 (469) | 692 (836) | 3  (2) | 0  (1) | 631 (489) | 674 (816) | 3  (2) | 0  (1) | 630 (490) | 675 (815) | 3  (2) | 0  (1) | 633 (498) | 672 (807) |
|  |  |  | *** |  |  |  | *** |  |  |  | *** |  |  |  | *** |  |
| **2080** | 2  (1) | 1  (2) | 497 (324) | 808 (981) | 2  (1) | 1  (2) | 542 (355) | 763 (950) | 2  (1) | 1  (2) | 538 (368) | 767 (937) | 2  (1) | 1  (2) | 563 (387) | 742 (918) |
|  | *** |  | *** |  | *** |  | *** |  | *** |  | *** |  | *** |  | *** |  |

**Table S1a (continued)**

|  | **A1FI** | | | | **A2** | | | | **B1** | | | | **B2** | | | |
| --- | --- | --- | --- | --- | --- | --- | --- | --- | --- | --- | --- | --- | --- | --- | --- | --- |
|  | **RL species** | | **all** | | **RL species** | | **all** | | **RL species** | | **all** | | **RL species** | | **all** | |
|  | **win** | **los** | **win** | **los** | **win** | **los** | **win** | **los** | **win** | **los** | **win** | **los** | **win** | **los** | **win** | **los** |
|  | |  |  |  |  |  |  |  |  |  |  |  |  |  |  |  |
| ***All taxa*** | |  |  |  |  |  |  |  |  |  |  |  |  |  |  |  |
| **2020** | 32  (20) | 21  (33) | 963 (807) | 924 (1082) | 32  (21) | 21  (32) | 979 (814) | 908 (1075) | 29  (21) | 24  (32) | 962 (798) | 925 (1091) | 30  (21) | 23  (32) | 978 (806) | 910 (1083) |
|  | *** |  | *** |  | *** |  | *** |  | ** |  | *** |  | *** |  | *** |  |
| **2050** | 25  (20) | 28  (33) | 885 (690) | 1003 (1199) | 26  (21) | 27  (32) | 910 (709) | 978 (1180) | 26  (21) | 27  (32) | 915 (715) | 973 (1174) | 25  (20) | 28  (33) | 911 (721) | 977 (1168) |
|  | ** |  | *** |  | ** |  | *** |  | *** |  | *** |  | *** |  | *** |  |
| **2080** | 21  (16) | 32  (47) | 725 (512) | 1163 (1377) | 23  (16) | 30  (37) | 791 (554) | 1097 (1335) | 24  (15) | 29  (38) | 812 (575) | 1076 (1314) | 27  (16) | 26  (37) | 841 (594) | 1047 (1295) |
|  | *** |  | *** |  | *** |  | *** |  | *** |  | *** |  | *** |  | *** |  |

**Table S1b –** Numbers of species projected to gain (win) and lose (los) climatic suitability in

Natura 2000 areas (in parenthesis is the expected value outside the Natura 2000), for different combinations of emission scenarios and time periods. Projections are provided for Habitat Directive species (HD species), and for the complete pool of vertebrate and plant species considered (all). Protected areas retaining more loser species than 95% of a set of similar sized random selected areas are marked with *** (*p*<0.001), ** (*p*<0.01), * (*p*<0.05).

|  | **A1FI** | | | | **A2** | | | | **B1** | | | | **B2** | | | |
| --- | --- | --- | --- | --- | --- | --- | --- | --- | --- | --- | --- | --- | --- | --- | --- | --- |
|  | **HD species** | | **All** | | **HD species** | | **all** | | **HD species** | | **all** | | **HD species** | | **all** | |
|  | **win** | **los** | **win** | **los** | **win** | **los** | **win** | **los** | **win** | **los** | **win** | **los** | **win** | **los** | **win** | **los** |
|  | |  |  |  |  |  |  |  |  |  |  |  |  |  |  |  |
| ***Amphibians*** | |  |  |  |  |  |  |  |  |  |  |  |  |  |  |  |
| **2020** | 23 (17) | 9  (15) | 28  (19) | 14  (23) | 23 (17) | 9  (15) | 28  (19) | 14  (23) | 24 (17) | 8  (15) | 31  (21) | 11  (21) | 24 (18) | 8  (14) | 31  (21) | 11  (21) |
|  | *** |  | *** |  | *** |  | *** |  | *** |  | *** |  | *** |  | *** |  |
| **2050** | 17 (14) | 15  (18) | 20  (17) | 22  (25) | 17 (16) | 15  (16) | 20  (19) | 22  (23) | 17 (16) | 15  (16) | 20  (19) | 22  (23) | 18 (17) | 14  (15) | 21  (20) | 21  (22) |
|  | ** |  | ** |  | ** |  | ** |  | ** |  | ** |  | ** |  | ** |  |
| **2080** | 11  (9) | 21  (23) | 13  (10) | 29  (32) | 11 (11) | 21  (21) | 13  (12) | 29  (30) | 12 (11) | 20  (21) | 14  (11) | 28  (31) | 15 (11) | 17  (21) | 18  (13) | 24  (29) |
|  |  |  | ** |  |  |  | ** |  |  |  | ** |  | *** |  | ** |  |
|  | |  |  |  |  |  |  |  |  |  |  |  |  |  |  |  |
| ***Reptiles*** | |  |  |  |  |  |  |  |  |  |  |  |  |  |  |  |
| **2020** | 23 (22) | 13  (14) | 40  (37) | 24  (27) | 23 (22) | 13  (14) | 40  (38) | 24  (26) | 22 (21) | 14  (15) | 41  (36) | 23  (28) | 24 (23) | 12  (13) | 42  (39) | 22  (25) |
|  |  |  | * |  |  |  | * |  |  |  | * |  |  |  | ** |  |
| **2050** | 25 (25) | 11  (11) | 36  (35) | 28  (29) | 26 (25) | 10  (11) | 36  (35) | 28  (29) | 25 (25) | 11  (11) | 37  (38) | 27  (26) | 25 (25) | 11  (11) | 37  (36) | 27  (28) |
|  |  |  |  |  |  |  |  |  |  |  |  |  |  |  |  |  |
| **2080** | 25 (22) | 11  (14) | 39  (35) | 25  (29) | 25 (23) | 11  (13) | 39  (36) | 25  (28) | 25 (23) | 11  (13) | 38  (35) | 26  (29) | 28 (24) | 8  (12) | 41  (37) | 23  (27) |
|  |  |  | * |  |  |  | * |  |  |  |  |  | ** |  | ** |  |
|  | |  |  |  |  |  |  |  |  |  |  |  |  |  |  |  |
| ***Birds*** | |  |  |  |  |  |  |  |  |  |  |  |  |  |  |  |
| **2020** | 61 (58) | 111 (114) | 130 (124) | 212 (219) | 63 (58) | 109 (114) | 134 (125) | 208 (218) | 59 (54) | 113 (118) | 131 (120) | 211 (223) | 61 (54) | 111 (118) | 134 (120) | 208 (223) |
|  | * |  | * |  | ** |  | *** |  |  |  | * |  | * |  | *** |  |
| **2050** | 66 (64) | 106 (108) | 128 (124) | 214 (219) | 67 (64) | 105 (108) | 129 (124) | 213 (219) | 65 (64) | 107 (108) | 128 (125) | 214 (218) | 64 (64) | 108 (108) | 126 (125) | 216 (218) |
|  |  |  |  |  |  |  |  |  |  |  |  |  |  |  |  |  |
| **2080** | 49 (42) | 123 (130) | 106 (97) | 236 (246) | 51 (48) | 121 (124) | 111 (105) | 231 (238) | 52 (50) | 120 (122) | 117 (114) | 225 (229) | 52 (49) | 120 (123) | 117 (112) | 225 (231) |
|  | *** |  | *** |  | *** |  | *** |  | *** |  | *** |  | *** |  | *** |  |
|  | |  |  |  |  |  |  |  |  |  |  |  |  |  |  |  |
| ***Mammals*** | |  |  |  |  |  |  |  |  |  |  |  |  |  |  |  |
| **2020** | 29 (23) | 25  (31) | 63  (50) | 72  (85) | 29 (23) | 25  (31) | 63  (50) | 72  (85) | 28 (23) | 26  (31) | 62  (48) | 73  (87) | 29 (23) | 25  (31) | 63  (51) | 72  (84) |
|  | *** |  | *** |  | *** |  | *** |  | *** |  | *** |  | *** |  | *** |  |
| **2050** | 20 (17) | 34  (37) | 43  (37) | 92  (98) | 20 (17) | 34  (37) | 46  (38) | 89  (97) | 23 (17) | 31  (37) | 49  (40) | 86  (95) | 23 (17) | 31  (37) | 49  (42) | 86  (93) |
|  | *** |  | *** |  | *** |  | *** |  | *** |  | *** |  | *** |  | *** |  |
| **2080** | 14 (13) | 40  (41) | 33  (31) | 102  (104) | 15 (12) | 39  (42) | 36  (30) | 99  (105) | 19 (15) | 35  (39) | 43  (35) | 92  (100) | 18 (15) | 36  (39) | 42  (35) | 93  (100) |
|  |  |  |  |  |  |  | *** |  | *** |  | *** |  | *** |  | *** |  |
|  | |  |  |  |  |  |  |  |  |  |  |  |  |  |  |  |
| ***Plants*** | |  |  |  |  |  |  |  |  |  |  |  |  |  |  |  |
| **2020** | 13 (13) | 15  (15) | 581 (535) | 724 (770) | 13 (13) | 15  (15) | 585 (535) | 720 (770) | 13 (13) | 15  (15) | 571 (511) | 734 (794) | 14 (13) | 14  (15) | 586 (519) | 719 (786) |
|  |  | *** | *** |  |  | *** | *** |  |  | *** | *** |  |  | ** | *** |  |
| **2050** | 13 (13) | 15  (15) | 512 (448) | 793 (857) | 13 (13) | 15  (15) | 523 (458) | 782 (847) | 13 (13) | 15  (15) | 531 (456) | 774 (849) | 15 (15) | 13  (13) | 537 (476) | 768 (829) |
|  |  | *** | *** |  |  | *** | *** |  |  | *** | *** |  |  | ** | *** |  |
| **2080** | 11 (11) | 17  (17) | 363 (316) | 942 (989) | 11 (13) | 17  (15) | 398 (350) | 907 (955) | 12 (13) | 16  (15) | 407 (356) | 897 (949) | 13 (14) | 15  (14) | 432 (382) | 873 (923) |
|  |  | *** | *** |  |  | *** | *** |  |  | *** | *** |  |  | ** | *** |  |

**Table S1b (continued)**

|  | **A1FI** | | | | **A2** | | | | **B1** | | | | **B2** | | | |
| --- | --- | --- | --- | --- | --- | --- | --- | --- | --- | --- | --- | --- | --- | --- | --- | --- |
|  | **HD species** | | **all** | | **HD species** | | **all** | | **HD species** | | **all** | | **HD species** | | **all** | |
|  | **win** | **los** | **win** | **los** | **win** | **los** | **win** | **los** | **win** | **los** | **win** | **los** | **win** | **los** | **win** | **los** |
|  | |  |  |  |  |  |  |  |  |  |  |  |  |  |  |  |
| ***All taxa*** | |  |  |  |  |  |  |  |  |  |  |  |  |  |  |  |
| **2020** | 149 (133) | 173  (189) | 842 (765) | 1046 (1124) | 151 (133) | 171  (189) | 850 (767) | 1038 (1122) | 146 (128) | 176  (194) | 836 (736) | 1052 (1153) | 152 (131) | 170  (191) | 856 (750) | 1032 (1139) |
|  | * |  | *** |  | * |  | *** |  | * |  | *** |  | * |  | *** |  |
| **2050** | 141 (133) | 181  (189) | 739 (661) | 1149 (1228) | 143 (135) | 179  (187) | 754 (674) | 1134 (1215) | 143 (135) | 179  (187) | 765 (678) | 1123 (1211) | 145 (138) | 177  (184) | 770 (699) | 1118 (1190) |
|  | * |  | *** |  | * |  | *** |  | * |  | *** |  | * |  | *** |  |
| **2080** | 110 (97) | 212  (225) | 554 (489) | 1334 (1400) | 113 (107) | 209  (215) | 597 (533) | 1291 (1356) | 121 (112) | 202  (210) | 619 (551) | 1268 (1338) | 126 (113) | 196  (209) | 650 (579) | 1238 (1310) |
|  | * |  | *** |  |  |  | *** |  | * |  | *** |  | * |  | *** |  |

**Table S2 -** Percentage of projected loser species. Values are provided for different combinations of suites of species, conservation areas, emission scenarios and years. Significant pairwise differences between means were assessed with Mcnemar tests for the complete pool of species in protected areas as well as for the Habitat Directive species in NATURA sites. Wilcoxon-signed rank tests are provided for Red-listed species in protected areas. Letters *a* to *e* code the pairs of emission scenarios with proportions of loser species projected to be different with the following levels of significance ****p*<0.001, ** *p*<0.01, * *p*<0.05. For the all-species in protected areas set, the A1FI scenario for 2020 differs from the A2 scenario (*a*, *p*<0.01), the A2 scenario differs from the B1 scenario (*b*, *p*<0.05), and the B1 scenario differs from the B2 scenario (*c*, *p*<0.05).

|  |  | **A1FI** | **A2** | **B1** | **B2** | **mean±SD** |
| --- | --- | --- | --- | --- | --- | --- |
|  |  |  |  |  |  | |
| ***all species*** | |  |  |  |  | |
| **Protected áreas** | 2020 | 48.91 | 48.07 | 48.97 | 48.17 | 48.53±0.48 |
|  |  | *a*** | *a**,b** | *b*,c** | *c** |  |
|  |  |  |  |  |  |  |
|  | 2050 | 53.10 | 51.77 | 51.51 | 51.72 | 52.02±0.72 |
|  |  | *a***,b***,c**** | *a**** | *B**** | *c**** |  |
|  |  |  |  |  |  |  |
|  | 2080 | 61.57 | 58.07 | 56.96 | 55.43 | 58.01±2.61 |
|  |  | *a***,b***,c**** | *a***,d**** | *b***,e**** | *c***,d***,e**** |  |
|  |  |  |  |  |  |  |
| ***priority species*** | |  |  |  |  | |
| **Protected areas** | 2020 | 39.62 | 39.62 | 45.28 | 43.40 | 41.98±2.83 |
|  |  |  |  |  |  |  |
|  |  |  |  |  |  |  |
|  | 2050 | 52.83 | 50.94 | 50.94 | 52.83 | 51.89±1.09 |
|  |  |  |  |  |  |  |
|  |  |  |  |  |  |  |
|  | 2080 | 60.38 | 56.60 | 54.72 | 49.06 | 55.19±4.72 |
|  |  | *a** | *b** |  | *a*, b** |  |
| **Natura 2000** | 2020 | 53.56 | 52.94 | 54.49 | 52.63 | 53.41±0.82 |
|  |  |  |  |  |  |  |
|  |  |  |  |  |  |  |
|  | 2050 | 56.04 | 55.42 | 55.42 | 54.80 | 55.42±0.51 |
|  |  |  |  |  |  |  |
|  |  |  |  |  |  |  |
|  | 2080 | 65.63 | 64.71 | 62.54 | 60.68 | 63.39±2.22 |
|  |  | a** | b** | c* | a**,b**,c* |  |
|  |  |  |  |  |  |  |

**Table S3a – Number of species projected to gain (win) and lose (los) climatic suitability in protected areas (in parenthesis: outside protected areas), for the different countries, climatic storylines and analysed years. Projections are provided for Red-List species (RL species) and for all species (all).**

|  |  | **A1FI** | | | | **A2** | | | | **B1** | | | | **B2** | | | |
| --- | --- | --- | --- | --- | --- | --- | --- | --- | --- | --- | --- | --- | --- | --- | --- | --- | --- |
|  |  | **all species** | | **RL species** | | **all species** | | **RL species** | | **all species** | | **RL species** | | **all species** | | **RL species** | |
|  |  | **win** | **los** | **win** | **los** | **win** | **los** | **win** | **los** | **win** | **los** | **win** | **los** | **win** | **los** | **win** | **los** |

| ***Amphibians*** | |  |  |  |  |  |  |  |  |  |  |  |  |  |  |  |  |
| --- | --- | --- | --- | --- | --- | --- | --- | --- | --- | --- | --- | --- | --- | --- | --- | --- | --- |
| **Albania** | **2020** | 7 (7) | 8 (8) | 0 (0) | 0 (0) | 7 (7) | 8 (8) | 0 (0) | 0 (0) | 7 (6) | 8 (9) | 0 (0) | 0 (0) | 7 (7) | 8 (8) | 0 (0) | 0 (0) |
|  | **2050** | 10 (10) | 5 (5) | 0 (0) | 0 (0) | 10 (10) | 5 (5) | 0 (0) | 0 (0) | 10 (10) | 5 (5) | 0 (0) | 0 (0) | 10 (10) | 5 (5) | 0 (0) | 0 (0) |
|  | **2080** | 6 (5) | 9 (10) | 0 (0) | 0 (0) | 6 (5) | 9 (10) | 0 (0) | 0 (0) | 6 (3) | 9 (12) | 0 (0) | 0 (0) | 6 (5) | 9 (10) | 0 (0) | 0 (0) |
| **Andorra** | **2020** | 7 (8) | 1 (0) | 1 (1) | 0 (0) | 7 (8) | 1 (0) | 1 (1) | 0 (0) | 7 (8) | 1 (0) | 1 (1) | 0 (0) | 7 (8) | 1 (0) | 1 (1) | 0 (0) |
|  | **2050** | 7 (7) | 1 (1) | 1 (1) | 0 (0) | 8 (8) | 0 (0) | 1 (1) | 0 (0) | 8 (8) | 0 (0) | 1 (1) | 0 (0) | 8 (7) | 0 (0) | 1 (1) | 0 (0) |
|  | **2080** | 7 (7) | 1 (1) | 1 (1) | 0 (0) | 6 (7) | 2 (1) | 1 (1) | 0 (0) | 7 (6) | 1 (2) | 1 (1) | 0 (0) | 7 (6) | 1 (2) | 1 (1) | 0 (0) |
| **Austria** | **2020** | 13 (12) | 7 (8) | 0 (0) | 0 (0) | 13 (12) | 7 (8) | 0 (0) | 0 (0) | 13 (12) | 7 (8) | 0 (0) | 0 (0) | 12 (12) | 8 (8) | 0 (0) | 0 (0) |
|  | **2050** | 14 (13) | 6 (7) | 0 (0) | 0 (0) | 16 (15) | 4 (5) | 0 (0) | 0 (0) | 14 (14) | 5 (5) | 0 (0) | 0 (0) | 14 (15) | 5 (5) | 0 (0) | 0 (0) |
|  | **2080** | 14 (10) | 6 (10) | 0 (0) | 0 (0) | 14 (12) | 6 (8) | 0 (0) | 0 (0) | 14 (15) | 4 (5) | 0 (0) | 0 (0) | 14 (14) | 4 (5) | 0 (0) | 0 (0) |
| **Belgium** | **2020** | 14 (13) | 4 (6) | 0 (0) | 0 (0) | 13 (13) | 4 (6) | 0 (0) | 0 (0) | 14 (13) | 4 (6) | 0 (0) | 0 (0) | 13 (12) | 5 (7) | 0 (0) | 0 (0) |
|  | **2050** | 12 (5) | 7 (14) | 0 (0) | 0 (0) | 12 (5) | 7 (14) | 0 (0) | 0 (0) | 12 (5) | 7 (14) | 0 (0) | 0 (0) | 12 (5) | 7 (14) | 0 (0) | 0 (0) |
|  | **2080** | 5 (3) | 14 (16) | 0 (0) | 0 (0) | 8 (4) | 11 (15) | 0 (0) | 0 (0) | 8 (7) | 11 (12) | 0 (0) | 0 (0) | 6 (6) | 13 (13) | 0 (0) | 0 (0) |
| **Bosnia and**  **Herzeg** | **2020** | 10 (6) | 9 (13) | 1 (1) | 0 (0) | 10 (7) | 9 (12) | 1 (1) | 0 (0) | 7 (6) | 11 (13) | 1 (1) | 0 (0) | 8 (6) | 10 (13) | 1 (1) | 0 (0) |
|  | **2050** | 11 (9) | 8 (10) | 1 (1) | 0 (0) | 12 (9) | 7 (10) | 1 (1) | 0 (0) | 12 (8) | 7 (11) | 1 (1) | 0 (0) | 12 (9) | 7 (10) | 1 (1) | 0 (0) |
|  | **2080** | 7 (7) | 12 (12) | 0 (1) | 1 (0) | 11 (8) | 8 (11) | 1 (1) | 0 (0) | 8 (8) | 10 (11) | 0 (1) | 0 (0) | 10 (8) | 9 (11) | 1 (1) | 0 (0) |
| **Bulgaria** | **2020** | 10 (7) | 7 (10) | 0 (0) | 0 (0) | 11 (7) | 6 (10) | 0 (0) | 0 (0) | 11 (7) | 6 (10) | 0 (0) | 0 (0) | 11 (7) | 6 (10) | 0 (0) | 0 (0) |
|  | **2050** | 12 (10) | 5 (7) | 0 (0) | 0 (0) | 12 (11) | 5 (6) | 0 (0) | 0 (0) | 13 (10) | 4 (7) | 0 (0) | 0 (0) | 13 (11) | 4 (6) | 0 (0) | 0 (0) |
|  | **2080** | 9 (9) | 8 (8) | 0 (0) | 0 (0) | 9 (9) | 8 (8) | 0 (0) | 0 (0) | 11 (9) | 6 (8) | 0 (0) | 0 (0) | 10 (10) | 7 (7) | 0 (0) | 0 (0) |
| **Croatia** | **2020** | 6 (4) | 13 (15) | 2 (2) | 0 (0) | 6 (4) | 13 (15) | 2 (2) | 0 (0) | 4 (4) | 15 (15) | 1 (2) | 1 (0) | 6 (4) | 13 (15) | 2 (2) | 0 (0) |
|  | **2050** | 6 (6) | 13 (13) | 2 (2) | 0 (0) | 6 (6) | 13 (13) | 2 (2) | 0 (0) | 6 (6) | 13 (13) | 2 (2) | 0 (0) | 6 (6) | 13 (13) | 2 (2) | 0 (0) |
|  | **2080** | 5 (5) | 14 (14) | 2 (2) | 0 (0) | 7 (6) | 12 (13) | 2 (2) | 0 (0) | 5 (5) | 14 (14) | 2 (2) | 0 (0) | 5 (5) | 14 (14) | 2 (2) | 0 (0) |
| **Czech Republic** | **2020** | 17 (11) | 2 (8) | 0 (0) | 0 (0) | 17 (11) | 2 (8) | 0 (0) | 0 (0) | 17 (13) | 2 (6) | 0 (0) | 0 (0) | 17 (13) | 2 (6) | 0 (0) | 0 (0) |
|  | **2050** | 13 (10) | 6 (9) | 0 (0) | 0 (0) | 14 (13) | 5 (6) | 0 (0) | 0 (0) | 14 (13) | 5 (6) | 0 (0) | 0 (0) | 14 (13) | 5 (6) | 0 (0) | 0 (0) |
|  | **2080** | 11 (7) | 8 (12) | 0 (0) | 0 (0) | 13 (10) | 6 (9) | 0 (0) | 0 (0) | 14 (12) | 5 (7) | 0 (0) | 0 (0) | 14 (12) | 5 (7) | 0 (0) | 0 (0) |
| **Denmark** | **2020** | 10 (10) | 4 (4) | 0 (0) | 0 (0) | 10 (10) | 4 (4) | 0 (0) | 0 (0) | 10 (10) | 4 (4) | 0 (0) | 0 (0) | 10 (10) | 4 (4) | 0 (0) | 0 (0) |
|  | **2050** | 9 (10) | 5 (4) | 0 (0) | 0 (0) | 10 (10) | 4 (4) | 0 (0) | 0 (0) | 9 (10) | 5 (4) | 0 (0) | 0 (0) | 9 (9) | 5 (5) | 0 (0) | 0 (0) |
|  | **2080** | 6 (7) | 8 (8) | 0 (0) | 0 (0) | 7 (7) | 7 (7) | 0 (0) | 0 (0) | 7 (8) | 7 (6) | 0 (0) | 0 (0) | 7 (7) | 7 (7) | 0 (0) | 0 (0) |
| **Estonia** | **2020** | 11 (11) | 0 (0) | 0 (0) | 0 (0) | 11 (11) | 0 (0) | 0 (0) | 0 (0) | 11 (11) | 0 (0) | 0 (0) | 0 (0) | 11 (11) | 0 (0) | 0 (0) | 0 (0) |
|  | **2050** | 11 (11) | 0 (0) | 0 (0) | 0 (0) | 11 (11) | 0 (0) | 0 (0) | 0 (0) | 11 (10) | 0 (1) | 0 (0) | 0 (0) | 11 (11) | 0 (0) | 0 (0) | 0 (0) |
|  | **2080** | 11 (11) | 0 (0) | 0 (0) | 0 (0) | 11 (11) | 0 (0) | 0 (0) | 0 (0) | 11 (11) | 0 (0) | 0 (0) | 0 (0) | 11 (11) | 0 (0) | 0 (0) | 0 (0) |
| **Finland** | **2020** | 5 (4) | 0 (1) | 0 (0) | 0 (0) | 5 (4) | 0 (1) | 0 (0) | 0 (0) | 5 (4) | 0 (1) | 0 (0) | 0 (0) | 5 (4) | 0 (1) | 0 (0) | 0 (0) |
|  | **2050** | 5 (4) | 0 (1) | 0 (0) | 0 (0) | 5 (4) | 0 (1) | 0 (0) | 0 (0) | 5 (4) | 0 (1) | 0 (0) | 0 (0) | 5 (4) | 0 (1) | 0 (0) | 0 (0) |
|  | **2080** | 5 (4) | 0 (1) | 0 (0) | 0 (0) | 5 (4) | 0 (1) | 0 (0) | 0 (0) | 5 (4) | 0 (1) | 0 (0) | 0 (0) | 5 (4) | 0 (1) | 0 (0) | 0 (0) |
| **France** | **2020** | 15 (9) | 12 (18) | 2 (1) | 0 (1) | 15 (9) | 12 (18) | 2 (1) | 0 (1) | 15 (9) | 12 (18) | 2 (1) | 0 (1) | 14 (9) | 13 (18) | 2 (1) | 0 (1) |
|  | **2050** | 11 (6) | 16 (21) | 2 (1) | 0 (1) | 11 (4) | 16 (23) | 2 (1) | 0 (1) | 13 (3) | 14 (24) | 2 (1) | 0 (1) | 13 (5) | 14 (22) | 2 (1) | 0 (1) |
|  | **2080** | 13 (8) | 15 (20) | 1 (1) | 1 (1) | 12 (7) | 16 (20) | 1 (1) | 1 (1) | 13 (5) | 15 (22) | 2 (1) | 0 (1) | 13 (7) | 15 (20) | 2 (1) | 0 (1) |
| **Germany** | **2020** | 12 (10) | 8 (10) | 0 (0) | 0 (0) | 12 (10) | 8 (10) | 0 (0) | 0 (0) | 12 (11) | 8 (9) | 0 (0) | 0 (0) | 12 (11) | 8 (9) | 0 (0) | 0 (0) |
|  | **2050** | 10 (9) | 10 (11) | 0 (0) | 0 (0) | 10 (10) | 10 (10) | 0 (0) | 0 (0) | 11 (11) | 9 (9) | 0 (0) | 0 (0) | 10 (9) | 10 (11) | 0 (0) | 0 (0) |
|  | **2080** | 3 (3) | 17 (17) | 0 (0) | 0 (0) | 5 (4) | 15 (16) | 0 (0) | 0 (0) | 8 (6) | 12 (14) | 0 (0) | 0 (0) | 6 (5) | 14 (15) | 0 (0) | 0 (0) |
| **Greece** | **2020** | 4 (2) | 10 (13) | 0 (0) | 0 (0) | 4 (2) | 9 (13) | 0 (0) | 0 (0) | 4 (2) | 10 (13) | 0 (0) | 0 (0) | 4 (2) | 10 (13) | 0 (0) | 0 (0) |
|  | **2050** | 9 (7) | 6 (8) | 0 (0) | 0 (0) | 10 (9) | 5 (6) | 0 (0) | 0 (0) | 10 (9) | 5 (6) | 0 (0) | 0 (0) | 10 (10) | 5 (5) | 0 (0) | 0 (0) |
|  | **2080** | 3 (2) | 11 (13) | 0 (0) | 0 (0) | 4 (2) | 10 (13) | 0 (0) | 0 (0) | 3 (1) | 12 (14) | 0 (0) | 0 (0) | 4 (2) | 10 (13) | 0 (0) | 0 (0) |
| **Hungary** | **2020** | 1 (1) | 15 (15) | 0 (0) | 0 (0) | 1 (2) | 15 (14) | 0 (0) | 0 (0) | 2 (1) | 14 (15) | 0 (0) | 0 (0) | 2 (2) | 14 (14) | 0 (0) | 0 (0) |
|  | **2050** | 4 (4) | 12 (12) | 0 (0) | 0 (0) | 4 (5) | 12 (11) | 0 (0) | 0 (0) | 5 (6) | 11 (10) | 0 (0) | 0 (0) | 6 (6) | 10 (10) | 0 (0) | 0 (0) |
|  | **2080** | 6 (7) | 10 (9) | 0 (0) | 0 (0) | 5 (8) | 11 (8) | 0 (0) | 0 (0) | 5 (8) | 11 (8) | 0 (0) | 0 (0) | 5 (8) | 11 (8) | 0 (0) | 0 (0) |
| **Ireland** | **2020** | 2 (2) | 1 (1) | 0 (0) | 0 (0) | 2 (2) | 1 (1) | 0 (0) | 0 (0) | 2 (2) | 1 (1) | 0 (0) | 0 (0) | 2 (2) | 1 (1) | 0 (0) | 0 (0) |
|  | **2050** | 3 (1) | 0 (2) | 0 (0) | 0 (0) | 3 (1) | 0 (2) | 0 (0) | 0 (0) | 3 (1) | 0 (2) | 0 (0) | 0 (0) | 3 (1) | 0 (2) | 0 (0) | 0 (0) |
|  | **2080** | 1 (1) | 2 (2) | 0 (0) | 0 (0) | 3 (1) | 0 (2) | 0 (0) | 0 (0) | 3 (1) | 0 (2) | 0 (0) | 0 (0) | 3 (1) | 0 (2) | 0 (0) | 0 (0) |
| **Italy** | **2020** | 20 (16) | 7 (12) | 1 (2) | 1 (0) | 20 (15) | 7 (13) | 1 (2) | 1 (0) | 20 (15) | 6 (12) | 1 (2) | 1 (0) | 20 (15) | 7 (13) | 1 (2) | 1 (0) |
|  | **2050** | 21 (17) | 6 (11) | 2 (2) | 0 (0) | 20 (17) | 7 (11) | 2 (2) | 0 (0) | 19 (17) | 8 (11) | 2 (2) | 0 (0) | 20 (17) | 7 (11) | 2 (2) | 0 (0) |
|  | **2080** | 17 (14) | 10 (14) | 2 (2) | 0 (0) | 17 (15) | 10 (13) | 2 (2) | 0 (0) | 19 (15) | 8 (13) | 2 (1) | 0 (1) | 19 (15) | 8 (13) | 2 (2) | 0 (0) |
| **Latvia** | **2020** | 12 (12) | 0 (0) | 0 (0) | 0 (0) | 12 (12) | 0 (0) | 0 (0) | 0 (0) | 12 (12) | 0 (0) | 0 (0) | 0 (0) | 12 (12) | 0 (0) | 0 (0) | 0 (0) |
|  | **2050** | 12 (12) | 0 (0) | 0 (0) | 0 (0) | 12 (12) | 0 (0) | 0 (0) | 0 (0) | 12 (12) | 0 (0) | 0 (0) | 0 (0) | 12 (12) | 0 (0) | 0 (0) | 0 (0) |
|  | **2080** | 10 (9) | 2 (3) | 0 (0) | 0 (0) | 12 (11) | 0 (1) | 0 (0) | 0 (0) | 12 (11) | 0 (1) | 0 (0) | 0 (0) | 12 (11) | 0 (1) | 0 (0) | 0 (0) |
| **Liechten-stein** | **2020** | 7 (8) | 6 (6) | 0 (0) | 0 (0) | 8 (8) | 6 (6) | 0 (0) | 0 (0) | 8 (8) | 6 (6) | 0 (0) | 0 (0) | 7 (8) | 6 (6) | 0 (0) | 0 (0) |
|  | **2050** | 9 (9) | 5 (5) | 0 (0) | 0 (0) | 14 (14) | 0 (0) | 0 (0) | 0 (0) | 14 (14) | 0 (0) | 0 (0) | 0 (0) | 14 (14) | 0 (0) | 0 (0) | 0 (0) |
|  | **2080** | 11 (11) | 3 (3) | 0 (0) | 0 (0) | 13 (13) | 1 (1) | 0 (0) | 0 (0) | 13 (13) | 1 (1) | 0 (0) | 0 (0) | 13 (13) | 1 (1) | 0 (0) | 0 (0) |
| **Lithuania** | **2020** | 12 (12) | 1 (1) | 0 (0) | 0 (0) | 12 (12) | 1 (1) | 0 (0) | 0 (0) | 12 (12) | 1 (1) | 0 (0) | 0 (0) | 12 (12) | 1 (1) | 0 (0) | 0 (0) |
|  | **2050** | 12 (12) | 1 (1) | 0 (0) | 0 (0) | 12 (12) | 1 (1) | 0 (0) | 0 (0) | 12 (12) | 1 (1) | 0 (0) | 0 (0) | 12 (12) | 1 (1) | 0 (0) | 0 (0) |
|  | **2080** | 9 (8) | 4 (5) | 0 (0) | 0 (0) | 10 (10) | 3 (3) | 0 (0) | 0 (0) | 12 (12) | 1 (1) | 0 (0) | 0 (0) | 12 (11) | 1 (2) | 0 (0) | 0 (0) |
| **Luxembourg** | **2020** | 14 (15) | 2 (4) | 0 (0) | 0 (0) | 14 (15) | 2 (4) | 0 (0) | 0 (0) | 14 (15) | 2 (4) | 0 (0) | 0 (0) | 14 (15) | 2 (4) | 0 (0) | 0 (0) |
|  | **2050** | 10 (9) | 7 (10) | 0 (0) | 0 (0) | 10 (11) | 7 (8) | 0 (0) | 0 (0) | 10 (11) | 7 (8) | 0 (0) | 0 (0) | 9 (9) | 8 (10) | 0 (0) | 0 (0) |
|  | **2080** | 4 (3) | 15 (16) | 0 (0) | 0 (0) | 8 (4) | 10 (15) | 0 (0) | 0 (0) | 9 (8) | 9 (11) | 0 (0) | 0 (0) | 9 (8) | 9 (11) | 0 (0) | 0 (0) |
| **Macedonia** | **2020** | 8 (9) | 6 (5) | 0 (0) | 0 (0) | 8 (9) | 6 (5) | 0 (0) | 0 (0) | 8 (10) | 6 (4) | 0 (0) | 0 (0) | 8 (10) | 6 (4) | 0 (0) | 0 (0) |
|  | **2050** | 10 (8) | 4 (6) | 0 (0) | 0 (0) | 10 (8) | 4 (6) | 0 (0) | 0 (0) | 10 (8) | 4 (6) | 0 (0) | 0 (0) | 10 (8) | 4 (6) | 0 (0) | 0 (0) |
|  | **2080** | 9 (7) | 5 (7) | 0 (0) | 0 (0) | 10 (7) | 4 (7) | 0 (0) | 0 (0) | 10 (8) | 4 (6) | 0 (0) | 0 (0) | 10 (9) | 4 (5) | 0 (0) | 0 (0) |
| **Malta** | **2020** | 2 (2) | 0 (0) | 0 (0) | 0 (0) | 2 (2) | 0 (0) | 0 (0) | 0 (0) | 2 (2) | 0 (0) | 0 (0) | 0 (0) | 2 (2) | 0 (0) | 0 (0) | 0 (0) |
|  | **2050** | 2 (2) | 0 (0) | 0 (0) | 0 (0) | 2 (2) | 0 (0) | 0 (0) | 0 (0) | 2 (2) | 0 (0) | 0 (0) | 0 (0) | 2 (2) | 0 (0) | 0 (0) | 0 (0) |
|  | **2080** | 2 (2) | 0 (0) | 0 (0) | 0 (0) | 2 (2) | 0 (0) | 0 (0) | 0 (0) | 2 (2) | 0 (0) | 0 (0) | 0 (0) | 2 (2) | 0 (0) | 0 (0) | 0 (0) |
| **Monaco** | **2020** | 0 (2) | 1 (3) | 0 (0) | 0 (0) | 0 (2) | 1 (3) | 0 (0) | 0 (0) | 0 (2) | 1 (3) | 0 (0) | 0 (0) | 0 (2) | 1 (3) | 0 (0) | 0 (0) |
|  | **2050** | 0 (1) | 1 (4) | 0 (0) | 0 (0) | 0 (1) | 1 (4) | 0 (0) | 0 (0) | 0 (0) | 1 (5) | 0 (0) | 0 (0) | 0 (1) | 1 (4) | 0 (0) | 0 (0) |
|  | **2080** | 0 (1) | 1 (4) | 0 (0) | 0 (0) | 0 (0) | 1 (5) | 0 (0) | 0 (0) | 0 (0) | 1 (5) | 0 (0) | 0 (0) | 0 (0) | 1 (5) | 0 (0) | 0 (0) |
| **Montenegro** | **2020** | 9 (10) | 5 (4) | 0 (0) | 0 (0) | 9 (10) | 5 (4) | 0 (0) | 0 (0) | 6 (10) | 8 (4) | 0 (0) | 0 (0) | 8 (10) | 6 (4) | 0 (0) | 0 (0) |
|  | **2050** | 9 (9) | 5 (5) | 0 (0) | 0 (0) | 9 (9) | 5 (5) | 0 (0) | 0 (0) | 8 (9) | 6 (5) | 0 (0) | 0 (0) | 9 (9) | 5 (5) | 0 (0) | 0 (0) |
|  | **2080** | 7 (8) | 7 (6) | 0 (0) | 0 (0) | 8 (9) | 6 (5) | 0 (0) | 0 (0) | 7 (9) | 7 (5) | 0 (0) | 0 (0) | 7 (9) | 7 (5) | 0 (0) | 0 (0) |
| **Netherlands** | **2020** | 9 (9) | 9 (9) | 0 (0) | 0 (0) | 9 (9) | 9 (9) | 0 (0) | 0 (0) | 9 (9) | 9 (9) | 0 (0) | 0 (0) | 8 (8) | 10 (10) | 0 (0) | 0 (0) |
|  | **2050** | 7 (7) | 11 (11) | 0 (0) | 0 (0) | 8 (8) | 10 (10) | 0 (0) | 0 (0) | 9 (9) | 9 (9) | 0 (0) | 0 (0) | 9 (8) | 9 (10) | 0 (0) | 0 (0) |
|  | **2080** | 5 (5) | 13 (13) | 0 (0) | 0 (0) | 5 (5) | 13 (13) | 0 (0) | 0 (0) | 6 (6) | 12 (12) | 0 (0) | 0 (0) | 6 (6) | 12 (12) | 0 (0) | 0 (0) |
| **Norway** | **2020** | 4 (6) | 1 (0) | 0 (0) | 0 (0) | 4 (6) | 1 (0) | 0 (0) | 0 (0) | 4 (6) | 1 (0) | 0 (0) | 0 (0) | 4 (6) | 1 (0) | 0 (0) | 0 (0) |
|  | **2050** | 5 (6) | 0 (0) | 0 (0) | 0 (0) | 5 (6) | 0 (0) | 0 (0) | 0 (0) | 5 (6) | 0 (0) | 0 (0) | 0 (0) | 5 (6) | 0 (0) | 0 (0) | 0 (0) |
|  | **2080** | 4 (5) | 1 (1) | 0 (0) | 0 (0) | 4 (5) | 1 (1) | 0 (0) | 0 (0) | 4 (5) | 1 (1) | 0 (0) | 0 (0) | 4 (5) | 1 (1) | 0 (0) | 0 (0) |
| **Poland** | **2020** | 15 (15) | 3 (3) | 0 (0) | 0 (0) | 15 (15) | 3 (3) | 0 (0) | 0 (0) | 15 (15) | 3 (3) | 0 (0) | 0 (0) | 15 (15) | 3 (3) | 0 (0) | 0 (0) |
|  | **2050** | 10 (9) | 8 (9) | 0 (0) | 0 (0) | 11 (10) | 7 (8) | 0 (0) | 0 (0) | 12 (11) | 6 (7) | 0 (0) | 0 (0) | 11 (11) | 7 (7) | 0 (0) | 0 (0) |
|  | **2080** | 8 (7) | 10 (11) | 0 (0) | 0 (0) | 8 (8) | 10 (10) | 0 (0) | 0 (0) | 10 (9) | 8 (9) | 0 (0) | 0 (0) | 10 (9) | 8 (9) | 0 (0) | 0 (0) |
| **Portugal** | **2020** | 9 (5) | 10 (14) | 2 (1) | 3 (4) | 8 (5) | 11 (14) | 2 (1) | 3 (4) | 9 (6) | 10 (13) | 2 (1) | 3 (4) | 10 (6) | 9 (13) | 2 (1) | 3 (4) |
|  | **2050** | 0 (2) | 19 (17) | 0 (1) | 5 (4) | 1 (2) | 18 (17) | 1 (1) | 4 (4) | 2 (2) | 17 (17) | 2 (1) | 3 (4) | 3 (3) | 16 (16) | 2 (1) | 3 (4) |
|  | **2080** | 2 (2) | 17 (17) | 1 (0) | 4 (5) | 2 (2) | 17 (17) | 1 (0) | 4 (5) | 2 (3) | 17 (16) | 1 (1) | 4 (4) | 2 (3) | 17 (16) | 1 (1) | 4 (4) |
| **Romania** | **2020** | 12 (9) | 6 (9) | 0 (0) | 0 (0) | 12 (9) | 6 (9) | 0 (0) | 0 (0) | 11 (9) | 7 (9) | 0 (0) | 0 (0) | 11 (9) | 7 (9) | 0 (0) | 0 (0) |
|  | **2050** | 14 (9) | 4 (9) | 0 (0) | 0 (0) | 13 (8) | 5 (10) | 0 (0) | 0 (0) | 14 (9) | 4 (9) | 0 (0) | 0 (0) | 14 (9) | 4 (9) | 0 (0) | 0 (0) |
|  | **2080** | 10 (7) | 8 (11) | 0 (0) | 0 (0) | 10 (8) | 8 (10) | 0 (0) | 0 (0) | 11 (8) | 7 (10) | 0 (0) | 0 (0) | 11 (8) | 7 (10) | 0 (0) | 0 (0) |
| **San Marino** | **2020** | 5 (5) | 9 (10) | 0 (0) | 0 (0) | 5 (6) | 9 (9) | 0 (0) | 0 (0) | 5 (4) | 9 (11) | 0 (0) | 0 (0) | 5 (6) | 9 (9) | 0 (0) | 0 (0) |
|  | **2050** | 5 (7) | 9 (8) | 0 (0) | 0 (0) | 5 (8) | 9 (7) | 0 (0) | 0 (0) | 5 (8) | 9 (7) | 0 (0) | 0 (0) | 5 (9) | 9 (6) | 0 (0) | 0 (0) |
|  | **2080** | 4 (5) | 10 (10) | 0 (0) | 0 (0) | 6 (5) | 8 (10) | 0 (0) | 0 (0) | 4 (5) | 10 (10) | 0 (0) | 0 (0) | 5 (5) | 9 (10) | 0 (0) | 0 (0) |
| **Serbia** | **2020** | 5 (6) | 14 (14) | 0 (0) | 0 (0) | 5 (6) | 14 (14) | 0 (0) | 0 (0) | 5 (7) | 14 (13) | 0 (0) | 0 (0) | 5 (7) | 14 (13) | 0 (0) | 0 (0) |
|  | **2050** | 7 (8) | 11 (12) | 0 (0) | 0 (0) | 10 (8) | 9 (12) | 0 (0) | 0 (0) | 10 (8) | 9 (12) | 0 (0) | 0 (0) | 9 (8) | 10 (12) | 0 (0) | 0 (0) |
|  | **2080** | 7 (8) | 12 (12) | 0 (0) | 0 (0) | 8 (8) | 11 (12) | 0 (0) | 0 (0) | 9 (8) | 10 (12) | 0 (0) | 0 (0) | 10 (11) | 9 (9) | 0 (0) | 0 (0) |
| **Slovakia** | **2020** | 16 (8) | 2 (10) | 0 (0) | 0 (0) | 16 (8) | 2 (10) | 0 (0) | 0 (0) | 16 (8) | 2 (10) | 0 (0) | 0 (0) | 16 (9) | 2 (9) | 0 (0) | 0 (0) |
|  | **2050** | 15 (8) | 3 (10) | 0 (0) | 0 (0) | 15 (8) | 3 (10) | 0 (0) | 0 (0) | 16 (7) | 2 (11) | 0 (0) | 0 (0) | 15 (7) | 3 (11) | 0 (0) | 0 (0) |
|  | **2080** | 12 (6) | 5 (12) | 0 (0) | 0 (0) | 14 (6) | 4 (12) | 0 (0) | 0 (0) | 14 (8) | 4 (10) | 0 (0) | 0 (0) | 14 (8) | 4 (10) | 0 (0) | 0 (0) |
| **Slovenia** | **2020** | 9 (5) | 10 (14) | 2 (2) | 0 (0) | 9 (5) | 10 (14) | 2 (2) | 0 (0) | 9 (5) | 10 (14) | 2 (2) | 0 (0) | 9 (5) | 10 (14) | 2 (2) | 0 (0) |
|  | **2050** | 12 (7) | 7 (12) | 2 (2) | 0 (0) | 13 (7) | 6 (12) | 2 (2) | 0 (0) | 12 (7) | 7 (12) | 2 (2) | 0 (0) | 12 (7) | 7 (12) | 2 (2) | 0 (0) |
|  | **2080** | 10 (5) | 9 (14) | 2 (2) | 0 (0) | 11 (6) | 8 (13) | 2 (2) | 0 (0) | 9 (5) | 10 (14) | 2 (2) | 0 (0) | 9 (5) | 10 (14) | 2 (2) | 0 (0) |
| **Spain** | **2020** | 21 (7) | 4 (18) | 6 (2) | 0 (4) | 20 (6) | 5 (19) | 6 (2) | 0 (4) | 20 (6) | 5 (19) | 6 (2) | 0 (4) | 21 (8) | 4 (17) | 6 (2) | 0 (4) |
|  | **2050** | 9 (4) | 15 (21) | 2 (0) | 4 (6) | 10 (4) | 15 (21) | 3 (0) | 3 (6) | 10 (5) | 14 (20) | 3 (0) | 3 (6) | 12 (5) | 13 (20) | 3 (1) | 3 (5) |
|  | **2080** | 8 (5) | 17 (20) | 1 (0) | 5 (6) | 8 (7) | 17 (18) | 2 (2) | 4 (4) | 9 (7) | 16 (18) | 2 (2) | 4 (4) | 10 (7) | 15 (18) | 2 (2) | 4 (4) |
| **Sweden** | **2020** | 9 (10) | 3 (2) | 0 (0) | 0 (0) | 9 (10) | 3 (2) | 0 (0) | 0 (0) | 9 (11) | 3 (1) | 0 (0) | 0 (0) | 9 (11) | 3 (1) | 0 (0) | 0 (0) |
|  | **2050** | 9 (11) | 3 (1) | 0 (0) | 0 (0) | 9 (11) | 3 (1) | 0 (0) | 0 (0) | 9 (11) | 3 (1) | 0 (0) | 0 (0) | 9 (11) | 3 (1) | 0 (0) | 0 (0) |
|  | **2080** | 8 (7) | 4 (5) | 0 (0) | 0 (0) | 9 (8) | 3 (4) | 0 (0) | 0 (0) | 9 (10) | 3 (2) | 0 (0) | 0 (0) | 9 (10) | 3 (2) | 0 (0) | 0 (0) |
| **Switzerland** | **2020** | 16 (16) | 4 (4) | 0 (0) | 0 (0) | 16 (16) | 4 (4) | 0 (0) | 0 (0) | 16 (16) | 4 (4) | 0 (0) | 0 (0) | 16 (16) | 4 (4) | 0 (0) | 0 (0) |
|  | **2050** | 16 (15) | 4 (5) | 0 (0) | 0 (0) | 16 (16) | 4 (4) | 0 (0) | 0 (0) | 16 (16) | 4 (4) | 0 (0) | 0 (0) | 16 (16) | 4 (4) | 0 (0) | 0 (0) |
|  | **2080** | 12 (8) | 8 (12) | 0 (0) | 0 (0) | 14 (10) | 6 (10) | 0 (0) | 0 (0) | 14 (11) | 6 (9) | 0 (0) | 0 (0) | 14 (10) | 6 (10) | 0 (0) | 0 (0) |
| **UK** | **2020** | 5 (3) | 3 (5) | 0 (0) | 0 (0) | 5 (3) | 3 (5) | 0 (0) | 0 (0) | 5 (4) | 3 (4) | 0 (0) | 0 (0) | 5 (3) | 3 (5) | 0 (0) | 0 (0) |
|  | **2050** | 4 (3) | 4 (5) | 0 (0) | 0 (0) | 3 (3) | 5 (5) | 0 (0) | 0 (0) | 3 (3) | 5 (5) | 0 (0) | 0 (0) | 3 (3) | 5 (5) | 0 (0) | 0 (0) |
|  | **2080** | 3 (4) | 5 (4) | 0 (0) | 0 (0) | 4 (4) | 4 (4) | 0 (0) | 0 (0) | 4 (4) | 4 (4) | 0 (0) | 0 (0) | 4 (3) | 4 (5) | 0 (0) | 0 (0) |

| ***Reptiles*** | |  |  |  |  |  |  |  |  |  |  |  |  |  |  |  |  |
| --- | --- | --- | --- | --- | --- | --- | --- | --- | --- | --- | --- | --- | --- | --- | --- | --- | --- |
| **Albania** | **2020** | 18 (16) | 13 (17) | 1 (1) | 0 (1) | 22 (18) | 10 (15) | 1 (1) | 0 (1) | 18 (17) | 13 (16) | 1 (1) | 0 (1) | 23 (18) | 9 (15) | 1 (1) | 0 (1) |
|  | **2050** | 23 (23) | 10 (10) | 1 (1) | 1 (1) | 23 (23) | 10 (10) | 1 (1) | 1 (1) | 24 (23) | 9 (10) | 2 (1) | 0 (1) | 23 (23) | 10 (10) | 1 (1) | 1 (1) |
|  | **2080** | 25 (26) | 8 (7) | 2 (2) | 0 (0) | 26 (27) | 7 (6) | 2 (2) | 0 (0) | 27 (27) | 6 (6) | 2 (2) | 0 (0) | 28 (27) | 5 (6) | 2 (2) | 0 (0) |
| **Andorra** | **2020** | 8 (13) | 1 (0) | 0 (0) | 0 (0) | 8 (12) | 1 (0) | 0 (0) | 0 (0) | 9 (12) | 1 (0) | 0 (0) | 0 (0) | 9 (11) | 1 (1) | 0 (0) | 0 (0) |
|  | **2050** | 9 (12) | 1 (2) | 0 (0) | 0 (0) | 10 (13) | 0 (2) | 0 (0) | 0 (0) | 9 (13) | 1 (2) | 0 (0) | 0 (0) | 10 (13) | 0 (1) | 0 (0) | 0 (0) |
|  | **2080** | 15 (15) | 0 (0) | 0 (0) | 0 (0) | 15 (15) | 0 (0) | 0 (0) | 0 (0) | 13 (14) | 1 (1) | 0 (0) | 0 (0) | 14 (14) | 1 (1) | 0 (0) | 0 (0) |
| **Austria** | **2020** | 12 (10) | 1 (3) | 1 (0) | 0 (1) | 12 (10) | 1 (3) | 1 (0) | 0 (1) | 12 (10) | 1 (3) | 1 (0) | 0 (1) | 12 (10) | 1 (3) | 1 (0) | 0 (1) |
|  | **2050** | 11 (10) | 1 (3) | 1 (0) | 0 (1) | 11 (10) | 1 (3) | 0 (0) | 0 (1) | 10 (10) | 2 (3) | 0 (0) | 0 (1) | 10 (10) | 3 (3) | 0 (0) | 1 (1) |
|  | **2080** | 11 (10) | 2 (3) | 1 (0) | 0 (1) | 12 (10) | 1 (3) | 1 (0) | 0 (1) | 12 (10) | 1 (3) | 1 (0) | 0 (1) | 12 (10) | 1 (3) | 1 (0) | 0 (1) |
| **Belgium** | **2020** | 4 (2) | 2 (5) | 0 (1) | 0 (0) | 3 (2) | 3 (5) | 0 (1) | 0 (0) | 3 (1) | 3 (5) | 0 (0) | 0 (0) | 3 (1) | 3 (6) | 0 (0) | 0 (1) |
|  | **2050** | 1 (2) | 5 (5) | 0 (0) | 1 (1) | 2 (2) | 5 (5) | 0 (0) | 1 (1) | 1 (2) | 6 (5) | 0 (0) | 1 (1) | 3 (3) | 4 (4) | 0 (0) | 1 (1) |
|  | **2080** | 3 (3) | 4 (4) | 1 (1) | 0 (0) | 3 (2) | 4 (5) | 1 (0) | 0 (1) | 3 (2) | 4 (5) | 1 (0) | 0 (1) | 3 (2) | 4 (5) | 1 (0) | 0 (1) |
| **Bosnia and**  **Herzeg** | **2020** | 16 (23) | 5 (2) | 0 (1) | 0 (0) | 16 (23) | 5 (2) | 0 (1) | 0 (0) | 15 (23) | 6 (2) | 0 (1) | 0 (0) | 15 (23) | 5 (2) | 0 (1) | 0 (0) |
|  | **2050** | 18 (21) | 4 (5) | 1 (2) | 0 (0) | 17 (20) | 4 (6) | 0 (2) | 0 (0) | 17 (21) | 4 (5) | 0 (2) | 0 (0) | 17 (21) | 4 (5) | 0 (2) | 0 (0) |
|  | **2080** | 18 (22) | 5 (4) | 2 (2) | 0 (0) | 18 (22) | 5 (4) | 2 (2) | 0 (0) | 16 (22) | 5 (4) | 0 (2) | 0 (0) | 17 (22) | 5 (4) | 1 (2) | 0 (0) |
| **Bulgaria** | **2020** | 25 (25) | 4 (4) | 2 (2) | 0 (0) | 23 (24) | 6 (5) | 2 (2) | 0 (0) | 24 (25) | 5 (4) | 2 (2) | 0 (0) | 24 (25) | 5 (4) | 2 (2) | 0 (0) |
|  | **2050** | 21 (20) | 8 (9) | 2 (2) | 0 (0) | 21 (20) | 8 (9) | 2 (2) | 0 (0) | 22 (20) | 7 (9) | 2 (2) | 0 (0) | 21 (21) | 8 (8) | 2 (2) | 0 (0) |
|  | **2080** | 25 (24) | 4 (5) | 2 (2) | 0 (0) | 25 (24) | 4 (5) | 2 (2) | 0 (0) | 25 (24) | 4 (5) | 2 (2) | 0 (0) | 25 (24) | 4 (5) | 2 (2) | 0 (0) |
| **Croatia** | **2020** | 24 (24) | 2 (4) | 0 (1) | 0 (0) | 24 (24) | 2 (4) | 0 (1) | 0 (0) | 24 (25) | 3 (3) | 1 (1) | 0 (0) | 25 (25) | 2 (3) | 1 (1) | 0 (0) |
|  | **2050** | 24 (23) | 3 (5) | 1 (1) | 0 (0) | 24 (23) | 3 (5) | 1 (1) | 0 (0) | 24 (23) | 3 (5) | 1 (1) | 0 (0) | 24 (23) | 3 (5) | 1 (1) | 0 (0) |
|  | **2080** | 24 (24) | 3 (4) | 1 (1) | 0 (0) | 24 (23) | 3 (5) | 1 (1) | 0 (0) | 24 (25) | 3 (3) | 1 (1) | 0 (0) | 24 (24) | 3 (4) | 1 (1) | 0 (0) |
| **Czech Republic** | **2020** | 10 (10) | 0 (0) | 1 (1) | 0 (0) | 10 (10) | 0 (0) | 1 (1) | 0 (0) | 10 (10) | 0 (0) | 1 (1) | 0 (0) | 10 (10) | 0 (0) | 1 (1) | 0 (0) |
|  | **2050** | 7 (6) | 3 (4) | 0 (0) | 1 (1) | 7 (7) | 3 (3) | 0 (0) | 1 (1) | 7 (7) | 3 (3) | 0 (0) | 1 (1) | 7 (7) | 3 (3) | 0 (0) | 1 (1) |
|  | **2080** | 7 (7) | 3 (3) | 0 (0) | 1 (1) | 7 (7) | 3 (3) | 0 (0) | 1 (1) | 8 (7) | 2 (3) | 0 (0) | 1 (1) | 8 (6) | 2 (4) | 0 (0) | 1 (1) |
| **Denmark** | **2020** | 5 (6) | 2 (1) | 1 (1) | 0 (0) | 5 (6) | 2 (1) | 1 (1) | 0 (0) | 5 (6) | 2 (1) | 1 (1) | 0 (0) | 5 (6) | 2 (1) | 1 (1) | 0 (0) |
|  | **2050** | 5 (5) | 2 (2) | 1 (1) | 0 (0) | 5 (6) | 2 (1) | 1 (1) | 0 (0) | 5 (6) | 2 (1) | 1 (1) | 0 (0) | 5 (6) | 2 (1) | 1 (1) | 0 (0) |
|  | **2080** | 5 (5) | 2 (2) | 1 (1) | 0 (0) | 5 (5) | 2 (2) | 1 (1) | 0 (0) | 5 (5) | 2 (2) | 1 (1) | 0 (0) | 5 (5) | 2 (2) | 1 (1) | 0 (0) |
| **Estonia** | **2020** | 4 (4) | 0 (1) | 0 (1) | 0 (0) | 4 (5) | 0 (0) | 0 (1) | 0 (0) | 4 (4) | 0 (1) | 0 (1) | 0 (0) | 4 (5) | 0 (0) | 0 (1) | 0 (0) |
|  | **2050** | 4 (3) | 0 (1) | 0 (0) | 0 (0) | 3 (3) | 1 (1) | 0 (0) | 0 (0) | 3 (3) | 1 (1) | 0 (0) | 0 (0) | 3 (3) | 1 (1) | 0 (0) | 0 (0) |
|  | **2080** | 4 (4) | 0 (0) | 0 (0) | 0 (0) | 4 (4) | 0 (0) | 0 (0) | 0 (0) | 4 (4) | 0 (0) | 0 (0) | 0 (0) | 4 (4) | 0 (0) | 0 (0) | 0 (0) |
| **Finland** | **2020** | 3 (4) | 0 (1) | 0 (1) | 0 (0) | 3 (4) | 0 (1) | 0 (1) | 0 (0) | 3 (4) | 0 (1) | 0 (1) | 0 (0) | 3 (4) | 0 (1) | 0 (1) | 0 (0) |
|  | **2050** | 3 (4) | 0 (1) | 0 (1) | 0 (0) | 3 (3) | 0 (1) | 0 (0) | 0 (0) | 3 (3) | 0 (2) | 0 (0) | 0 (1) | 3 (3) | 0 (2) | 0 (0) | 0 (1) |
|  | **2080** | 4 (4) | 0 (1) | 1 (1) | 0 (0) | 3 (4) | 0 (1) | 0 (1) | 0 (0) | 3 (4) | 0 (1) | 0 (1) | 0 (0) | 3 (4) | 0 (1) | 0 (1) | 0 (0) |
| **France** | **2020** | 21 (20) | 10 (13) | 3 (3) | 0 (1) | 21 (20) | 11 (13) | 3 (3) | 0 (1) | 21 (20) | 10 (13) | 3 (3) | 0 (1) | 21 (20) | 10 (13) | 3 (3) | 0 (1) |
|  | **2050** | 21 (19) | 12 (15) | 2 (2) | 1 (2) | 20 (18) | 12 (15) | 2 (2) | 1 (2) | 20 (18) | 12 (14) | 2 (2) | 1 (2) | 20 (18) | 12 (14) | 2 (2) | 1 (2) |
|  | **2080** | 22 (20) | 13 (15) | 2 (1) | 2 (3) | 23 (19) | 12 (15) | 3 (1) | 1 (3) | 24 (20) | 11 (14) | 3 (2) | 1 (2) | 23 (19) | 12 (15) | 2 (1) | 2 (3) |
| **Germany** | **2020** | 6 (6) | 6 (6) | 1 (1) | 0 (0) | 6 (6) | 6 (6) | 1 (1) | 0 (0) | 6 (6) | 6 (6) | 1 (1) | 0 (0) | 6 (6) | 6 (6) | 1 (1) | 0 (0) |
|  | **2050** | 8 (8) | 4 (4) | 0 (0) | 1 (1) | 7 (8) | 5 (4) | 0 (0) | 1 (1) | 8 (7) | 4 (5) | 0 (0) | 1 (1) | 7 (7) | 5 (5) | 0 (0) | 1 (1) |
|  | **2080** | 8 (8) | 4 (4) | 1 (1) | 0 (0) | 8 (8) | 4 (4) | 1 (1) | 0 (0) | 9 (9) | 3 (3) | 1 (1) | 0 (0) | 8 (8) | 4 (4) | 1 (1) | 0 (0) |
| **Greece** | **2020** | 15 (13) | 19 (24) | 1 (1) | 1 (1) | 19 (13) | 16 (24) | 1 (1) | 1 (1) | 17 (13) | 18 (24) | 1 (1) | 1 (1) | 23 (17) | 12 (20) | 1 (1) | 1 (1) |
|  | **2050** | 21 (22) | 14 (15) | 1 (1) | 1 (1) | 21 (23) | 14 (14) | 1 (1) | 1 (1) | 21 (22) | 14 (15) | 1 (1) | 1 (1) | 21 (23) | 14 (14) | 1 (1) | 1 (1) |
|  | **2080** | 24 (23) | 11 (14) | 1 (1) | 1 (1) | 24 (24) | 11 (13) | 1 (1) | 1 (1) | 25 (25) | 10 (12) | 1 (2) | 1 (0) | 25 (25) | 10 (12) | 1 (1) | 1 (1) |
| **Hungary** | **2020** | 10 (11) | 4 (3) | 1 (1) | 0 (0) | 10 (11) | 4 (3) | 1 (1) | 0 (0) | 10 (11) | 4 (3) | 1 (1) | 0 (0) | 10 (11) | 4 (3) | 1 (1) | 0 (0) |
|  | **2050** | 9 (9) | 5 (5) | 1 (1) | 0 (0) | 8 (9) | 5 (5) | 0 (1) | 0 (0) | 8 (10) | 6 (4) | 0 (1) | 1 (0) | 8 (9) | 5 (5) | 0 (0) | 0 (1) |
|  | **2080** | 11 (11) | 3 (3) | 1 (1) | 0 (0) | 11 (12) | 3 (2) | 1 (1) | 0 (0) | 12 (12) | 2 (2) | 1 (1) | 0 (0) | 12 (12) | 2 (2) | 1 (1) | 0 (0) |
| **Ireland** | **2020** | 1 (2) | 0 (0) | 0 (0) | 0 (0) | 1 (2) | 0 (0) | 0 (0) | 0 (0) | 1 (2) | 0 (0) | 0 (0) | 0 (0) | 1 (2) | 0 (0) | 0 (0) | 0 (0) |
|  | **2050** | 1 (1) | 1 (1) | 0 (0) | 0 (0) | 1 (1) | 1 (1) | 0 (0) | 0 (0) | 1 (1) | 1 (1) | 0 (0) | 0 (0) | 1 (1) | 1 (1) | 0 (0) | 0 (0) |
|  | **2080** | 1 (1) | 1 (1) | 0 (0) | 0 (0) | 1 (1) | 1 (1) | 0 (0) | 0 (0) | 1 (1) | 1 (1) | 0 (0) | 0 (0) | 1 (1) | 1 (1) | 0 (0) | 0 (0) |
| **Italy** | **2020** | 24 (23) | 14 (17) | 2 (2) | 2 (2) | 25 (23) | 13 (17) | 2 (2) | 2 (2) | 26 (24) | 12 (17) | 2 (2) | 2 (2) | 25 (24) | 13 (16) | 2 (2) | 2 (2) |
|  | **2050** | 25 (28) | 13 (13) | 3 (3) | 1 (1) | 25 (28) | 13 (13) | 3 (3) | 1 (1) | 25 (28) | 13 (13) | 3 (3) | 1 (1) | 27 (27) | 11 (14) | 3 (3) | 1 (1) |
|  | **2080** | 29 (27) | 10 (14) | 3 (2) | 1 (2) | 27 (27) | 11 (14) | 2 (2) | 2 (2) | 26 (26) | 12 (15) | 2 (2) | 2 (2) | 28 (26) | 10 (15) | 2 (2) | 2 (2) |
| **Latvia** | **2020** | 5 (6) | 0 (0) | 0 (1) | 0 (0) | 5 (6) | 0 (0) | 0 (1) | 0 (0) | 5 (6) | 0 (0) | 0 (1) | 0 (0) | 5 (6) | 0 (0) | 0 (1) | 0 (0) |
|  | **2050** | 5 (5) | 0 (0) | 0 (0) | 0 (0) | 5 (5) | 0 (0) | 0 (0) | 0 (0) | 5 (5) | 0 (0) | 0 (0) | 0 (0) | 5 (5) | 0 (0) | 0 (0) | 0 (0) |
|  | **2080** | 4 (4) | 1 (1) | 0 (0) | 0 (0) | 5 (6) | 0 (0) | 0 (0) | 0 (0) | 5 (5) | 0 (0) | 0 (0) | 0 (0) | 5 (5) | 0 (0) | 0 (0) | 0 (0) |
| **Liechten-stein** | **2020** | 5 (7) | 0 (0) | 0 (1) | 0 (0) | 5 (7) | 0 (0) | 0 (1) | 0 (0) | 5 (7) | 0 (0) | 0 (1) | 0 (0) | 5 (7) | 0 (0) | 0 (1) | 0 (0) |
|  | **2050** | 4 (4) | 1 (1) | 0 (0) | 0 (0) | 5 (5) | 0 (0) | 0 (0) | 0 (0) | 5 (5) | 0 (0) | 0 (0) | 0 (0) | 5 (5) | 0 (0) | 0 (0) | 0 (0) |
|  | **2080** | 5 (6) | 0 (0) | 0 (0) | 0 (0) | 5 (6) | 0 (0) | 0 (0) | 0 (0) | 5 (6) | 0 (0) | 0 (0) | 0 (0) | 5 (6) | 0 (0) | 0 (0) | 0 (0) |
| **Lithuania** | **2020** | 5 (5) | 0 (0) | 0 (0) | 0 (0) | 5 (5) | 0 (0) | 0 (0) | 0 (0) | 5 (5) | 0 (0) | 0 (0) | 0 (0) | 5 (6) | 0 (0) | 0 (0) | 0 (0) |
|  | **2050** | 5 (5) | 0 (0) | 0 (0) | 0 (0) | 5 (5) | 0 (0) | 0 (0) | 0 (0) | 5 (5) | 0 (0) | 0 (0) | 0 (0) | 5 (5) | 0 (0) | 0 (0) | 0 (0) |
|  | **2080** | 5 (4) | 0 (1) | 0 (0) | 0 (0) | 5 (6) | 0 (0) | 0 (0) | 0 (0) | 5 (5) | 0 (0) | 0 (0) | 0 (0) | 5 (6) | 0 (0) | 0 (0) | 0 (0) |
| **Luxembourg** | **2020** | 4 (5) | 1 (3) | 0 (1) | 0 (0) | 5 (5) | 1 (3) | 0 (1) | 0 (0) | 4 (5) | 1 (3) | 0 (1) | 0 (0) | 4 (5) | 1 (3) | 0 (1) | 0 (0) |
|  | **2050** | 4 (5) | 3 (3) | 0 (1) | 0 (0) | 5 (6) | 2 (2) | 0 (1) | 0 (0) | 5 (6) | 2 (2) | 0 (1) | 0 (0) | 5 (6) | 2 (2) | 0 (1) | 0 (0) |
|  | **2080** | 4 (5) | 3 (3) | 0 (1) | 0 (0) | 4 (5) | 3 (3) | 0 (1) | 0 (0) | 4 (5) | 3 (3) | 0 (1) | 0 (0) | 4 (5) | 3 (3) | 0 (1) | 0 (0) |
| **Macedonia** | **2020** | 21 (21) | 8 (8) | 1 (1) | 1 (1) | 21 (22) | 8 (7) | 1 (1) | 1 (1) | 22 (23) | 7 (5) | 1 (1) | 1 (1) | 22 (24) | 6 (5) | 1 (1) | 1 (1) |
|  | **2050** | 23 (21) | 7 (9) | 1 (1) | 1 (1) | 22 (21) | 8 (9) | 1 (1) | 1 (1) | 23 (22) | 7 (8) | 1 (1) | 1 (1) | 22 (21) | 7 (9) | 1 (1) | 1 (1) |
|  | **2080** | 25 (27) | 5 (3) | 1 (2) | 1 (0) | 25 (25) | 5 (5) | 1 (1) | 1 (1) | 25 (26) | 5 (4) | 1 (1) | 1 (1) | 25 (25) | 5 (5) | 1 (1) | 1 (1) |
| **Malta** | **2020** | 7 (9) | 0 (0) | 1 (1) | 0 (0) | 7 (9) | 0 (0) | 1 (1) | 0 (0) | 7 (9) | 0 (0) | 1 (1) | 0 (0) | 7 (9) | 0 (0) | 1 (1) | 0 (0) |
|  | **2050** | 8 (8) | 1 (1) | 1 (1) | 0 (0) | 8 (8) | 1 (1) | 1 (1) | 0 (0) | 8 (8) | 1 (1) | 1 (1) | 0 (0) | 8 (7) | 1 (1) | 1 (1) | 0 (0) |
|  | **2080** | 8 (9) | 1 (0) | 1 (1) | 0 (0) | 8 (9) | 1 (0) | 1 (1) | 0 (0) | 9 (9) | 0 (0) | 1 (1) | 0 (0) | 8 (9) | 1 (0) | 1 (1) | 0 (0) |
| **Monaco** | **2020** | 2 (12) | 0 (5) | 1 (1) | 0 (1) | 2 (11) | 0 (4) | 1 (1) | 0 (1) | 2 (11) | 0 (6) | 1 (1) | 0 (1) | 2 (11) | 0 (5) | 1 (1) | 0 (1) |
|  | **2050** | 3 (9) | 0 (8) | 1 (1) | 0 (1) | 3 (9) | 0 (8) | 1 (1) | 0 (1) | 3 (9) | 0 (8) | 1 (1) | 0 (1) | 3 (9) | 0 (8) | 1 (1) | 0 (1) |
|  | **2080** | 3 (6) | 2 (11) | 0 (0) | 0 (2) | 3 (9) | 2 (8) | 1 (1) | 0 (1) | 3 (9) | 1 (7) | 1 (1) | 0 (1) | 4 (9) | 0 (8) | 1 (1) | 0 (1) |
| **Montenegro** | **2020** | 25 (25) | 5 (5) | 0 (0) | 0 (0) | 25 (24) | 5 (6) | 0 (0) | 0 (0) | 25 (23) | 5 (7) | 0 (0) | 0 (0) | 25 (23) | 5 (7) | 0 (0) | 0 (0) |
|  | **2050** | 24 (24) | 7 (7) | 1 (1) | 0 (0) | 23 (24) | 7 (7) | 0 (1) | 0 (0) | 23 (24) | 7 (7) | 0 (1) | 0 (0) | 24 (24) | 7 (7) | 1 (1) | 0 (0) |
|  | **2080** | 25 (24) | 6 (7) | 1 (1) | 0 (0) | 25 (24) | 6 (7) | 1 (1) | 0 (0) | 23 (25) | 7 (6) | 0 (1) | 0 (0) | 23 (24) | 7 (7) | 0 (1) | 0 (0) |
| **Netherlands** | **2020** | 4 (3) | 3 (4) | 1 (1) | 0 (0) | 4 (3) | 3 (4) | 1 (1) | 0 (0) | 4 (2) | 3 (5) | 1 (0) | 0 (1) | 3 (2) | 4 (5) | 1 (0) | 0 (1) |
|  | **2050** | 3 (3) | 4 (4) | 0 (0) | 1 (1) | 3 (3) | 4 (4) | 0 (0) | 1 (1) | 3 (3) | 4 (4) | 0 (0) | 1 (1) | 3 (3) | 4 (4) | 0 (0) | 1 (1) |
|  | **2080** | 3 (3) | 4 (4) | 0 (0) | 1 (1) | 3 (3) | 4 (4) | 0 (0) | 1 (1) | 3 (3) | 4 (4) | 0 (0) | 1 (1) | 3 (3) | 4 (4) | 0 (0) | 1 (1) |
| **Norway** | **2020** | 4 (6) | 1 (0) | 1 (1) | 0 (0) | 4 (6) | 1 (0) | 1 (1) | 0 (0) | 4 (6) | 1 (0) | 1 (1) | 0 (0) | 4 (6) | 1 (0) | 1 (1) | 0 (0) |
|  | **2050** | 5 (6) | 0 (0) | 1 (1) | 0 (0) | 5 (6) | 0 (0) | 1 (1) | 0 (0) | 5 (6) | 0 (0) | 1 (1) | 0 (0) | 5 (6) | 0 (0) | 1 (1) | 0 (0) |
|  | **2080** | 5 (6) | 0 (0) | 1 (1) | 0 (0) | 5 (6) | 0 (0) | 1 (1) | 0 (0) | 5 (6) | 0 (0) | 1 (1) | 0 (0) | 5 (6) | 0 (0) | 1 (1) | 0 (0) |
| **Poland** | **2020** | 8 (8) | 1 (1) | 1 (1) | 0 (0) | 8 (8) | 1 (1) | 1 (1) | 0 (0) | 8 (8) | 1 (1) | 1 (1) | 0 (0) | 8 (8) | 1 (1) | 1 (1) | 0 (0) |
|  | **2050** | 7 (6) | 3 (4) | 0 (0) | 1 (1) | 7 (7) | 3 (3) | 0 (0) | 1 (1) | 7 (7) | 3 (3) | 0 (0) | 1 (1) | 6 (5) | 4 (5) | 0 (0) | 1 (1) |
|  | **2080** | 7 (7) | 3 (3) | 1 (1) | 0 (0) | 7 (7) | 3 (3) | 1 (1) | 0 (0) | 5 (6) | 4 (4) | 0 (0) | 1 (1) | 6 (7) | 4 (3) | 0 (1) | 1 (0) |
| **Portugal** | **2020** | 11 (10) | 17 (18) | 0 (0) | 4 (4) | 11 (10) | 17 (18) | 0 (0) | 4 (4) | 11 (10) | 16 (18) | 0 (0) | 4 (4) | 11 (10) | 17 (18) | 0 (0) | 4 (4) |
|  | **2050** | 10 (10) | 18 (18) | 1 (0) | 3 (4) | 10 (11) | 18 (17) | 1 (0) | 3 (4) | 12 (12) | 16 (16) | 1 (1) | 3 (3) | 12 (11) | 16 (17) | 1 (0) | 3 (4) |
|  | **2080** | 10 (9) | 18 (19) | 0 (0) | 4 (4) | 10 (10) | 18 (18) | 0 (0) | 4 (4) | 10 (10) | 18 (18) | 0 (0) | 4 (4) | 10 (10) | 18 (18) | 0 (0) | 4 (4) |
| **Romania** | **2020** | 19 (19) | 3 (3) | 2 (2) | 0 (0) | 19 (19) | 3 (3) | 2 (2) | 0 (0) | 19 (19) | 3 (3) | 2 (2) | 0 (0) | 19 (19) | 3 (3) | 2 (2) | 0 (0) |
|  | **2050** | 19 (17) | 3 (5) | 2 (2) | 0 (0) | 19 (17) | 3 (5) | 2 (2) | 0 (0) | 19 (17) | 3 (5) | 2 (2) | 0 (0) | 19 (17) | 3 (5) | 2 (2) | 0 (0) |
|  | **2080** | 20 (18) | 2 (4) | 2 (2) | 0 (0) | 20 (18) | 2 (4) | 2 (2) | 0 (0) | 19 (18) | 3 (4) | 2 (2) | 0 (0) | 19 (18) | 3 (4) | 2 (2) | 0 (0) |
| **San Marino** | **2020** | 8 (8) | 3 (5) | 0 (0) | 0 (0) | 7 (8) | 4 (5) | 0 (0) | 0 (0) | 9 (8) | 3 (5) | 0 (0) | 0 (0) | 9 (9) | 3 (4) | 0 (0) | 0 (0) |
|  | **2050** | 9 (8) | 3 (5) | 0 (0) | 0 (0) | 9 (8) | 3 (5) | 0 (0) | 0 (0) | 9 (8) | 3 (5) | 0 (0) | 0 (0) | 9 (9) | 3 (4) | 0 (0) | 0 (0) |
|  | **2080** | 9 (9) | 3 (4) | 0 (0) | 0 (0) | 9 (9) | 3 (4) | 0 (0) | 0 (0) | 9 (9) | 3 (4) | 0 (0) | 0 (0) | 9 (9) | 3 (4) | 0 (0) | 0 (0) |
| **Serbia** | **2020** | 21 (23) | 2 (6) | 1 (2) | 0 (0) | 20 (24) | 2 (5) | 1 (2) | 0 (0) | 22 (24) | 2 (5) | 1 (2) | 0 (0) | 20 (23) | 2 (6) | 1 (2) | 0 (0) |
|  | **2050** | 22 (26) | 3 (4) | 1 (2) | 0 (0) | 21 (26) | 4 (4) | 1 (2) | 0 (0) | 23 (27) | 2 (3) | 1 (2) | 0 (0) | 23 (25) | 2 (4) | 1 (2) | 0 (0) |
|  | **2080** | 22 (26) | 4 (4) | 1 (2) | 0 (0) | 22 (26) | 4 (4) | 1 (2) | 0 (0) | 22 (26) | 3 (4) | 1 (2) | 0 (0) | 22 (26) | 3 (4) | 1 (2) | 0 (0) |
| **Slovakia** | **2020** | 10 (8) | 0 (3) | 0 (1) | 0 (0) | 10 (8) | 0 (3) | 0 (1) | 0 (0) | 10 (9) | 0 (2) | 0 (1) | 0 (0) | 10 (9) | 0 (2) | 0 (1) | 0 (0) |
|  | **2050** | 9 (8) | 1 (3) | 0 (1) | 0 (0) | 9 (7) | 1 (3) | 0 (1) | 0 (0) | 8 (8) | 2 (3) | 0 (1) | 0 (0) | 9 (6) | 1 (5) | 0 (1) | 0 (0) |
|  | **2080** | 9 (8) | 1 (3) | 0 (1) | 0 (0) | 9 (9) | 1 (2) | 0 (1) | 0 (0) | 10 (9) | 0 (2) | 0 (1) | 0 (0) | 9 (9) | 1 (2) | 0 (1) | 0 (0) |
| **Slovenia** | **2020** | 17 (22) | 2 (4) | 1 (1) | 0 (0) | 18 (22) | 2 (4) | 1 (1) | 0 (0) | 17 (22) | 3 (4) | 1 (1) | 0 (0) | 17 (22) | 2 (4) | 1 (1) | 0 (0) |
|  | **2050** | 20 (22) | 2 (4) | 0 (1) | 0 (0) | 20 (22) | 2 (4) | 0 (1) | 0 (0) | 20 (22) | 2 (4) | 0 (1) | 0 (0) | 20 (22) | 2 (4) | 0 (1) | 0 (0) |
|  | **2080** | 21 (22) | 3 (4) | 1 (1) | 0 (0) | 20 (22) | 3 (4) | 0 (1) | 0 (0) | 20 (22) | 3 (4) | 1 (1) | 0 (0) | 20 (22) | 3 (4) | 1 (1) | 0 (0) |
| **Spain** | **2020** | 23 (18) | 13 (18) | 3 (2) | 2 (3) | 23 (18) | 13 (18) | 3 (2) | 2 (3) | 22 (17) | 14 (19) | 3 (2) | 2 (3) | 24 (18) | 12 (18) | 3 (2) | 2 (3) |
|  | **2050** | 17 (15) | 19 (21) | 3 (1) | 2 (4) | 19 (15) | 17 (21) | 3 (1) | 2 (4) | 17 (15) | 19 (21) | 2 (1) | 3 (4) | 21 (15) | 15 (21) | 4 (1) | 1 (4) |
|  | **2080** | 18 (16) | 18 (20) | 2 (1) | 3 (4) | 20 (16) | 16 (20) | 4 (1) | 1 (4) | 20 (16) | 16 (20) | 3 (1) | 2 (4) | 21 (17) | 15 (19) | 4 (2) | 1 (3) |
| **Sweden** | **2020** | 6 (6) | 0 (0) | 1 (1) | 0 (0) | 6 (6) | 0 (0) | 1 (1) | 0 (0) | 6 (6) | 0 (0) | 1 (1) | 0 (0) | 6 (6) | 0 (0) | 1 (1) | 0 (0) |
|  | **2050** | 4 (5) | 1 (1) | 0 (0) | 1 (1) | 5 (5) | 1 (1) | 0 (0) | 1 (1) | 5 (5) | 1 (1) | 0 (0) | 1 (1) | 5 (5) | 1 (1) | 0 (0) | 1 (1) |
|  | **2080** | 6 (5) | 0 (1) | 1 (1) | 0 (0) | 6 (6) | 0 (0) | 1 (1) | 0 (0) | 6 (6) | 0 (0) | 1 (1) | 0 (0) | 6 (6) | 0 (0) | 1 (1) | 0 (0) |
| **Switzerland** | **2020** | 15 (14) | 0 (1) | 1 (1) | 0 (0) | 15 (14) | 0 (1) | 1 (1) | 0 (0) | 15 (13) | 0 (2) | 1 (1) | 0 (0) | 15 (13) | 0 (2) | 1 (1) | 0 (0) |
|  | **2050** | 11 (12) | 4 (3) | 0 (1) | 1 (0) | 11 (12) | 4 (3) | 0 (1) | 1 (0) | 12 (12) | 3 (3) | 1 (1) | 0 (0) | 12 (12) | 3 (3) | 1 (1) | 0 (0) |
|  | **2080** | 11 (12) | 4 (3) | 1 (1) | 0 (0) | 11 (12) | 4 (3) | 1 (1) | 0 (0) | 11 (12) | 4 (3) | 1 (1) | 0 (0) | 11 (12) | 4 (3) | 1 (1) | 0 (0) |
| **UK** | **2020** | 3 (2) | 3 (4) | 1 (1) | 0 (0) | 3 (2) | 3 (4) | 1 (1) | 0 (0) | 2 (2) | 4 (4) | 1 (1) | 0 (0) | 3 (2) | 3 (4) | 1 (1) | 0 (0) |
|  | **2050** | 3 (2) | 3 (4) | 1 (0) | 0 (1) | 3 (2) | 3 (4) | 1 (0) | 0 (1) | 3 (3) | 3 (3) | 1 (1) | 0 (0) | 3 (2) | 3 (4) | 1 (0) | 0 (1) |
|  | **2080** | 3 (3) | 3 (3) | 1 (1) | 0 (0) | 3 (3) | 3 (3) | 1 (1) | 0 (0) | 3 (3) | 3 (3) | 1 (1) | 0 (0) | 3 (3) | 3 (3) | 1 (1) | 0 (0) |

| ***Birds*** | |  |  |  |  |  |  |  |  |  |  |  |  |  |  |  |  |
| --- | --- | --- | --- | --- | --- | --- | --- | --- | --- | --- | --- | --- | --- | --- | --- | --- | --- |
| **Albania** | **2020** | 87 (111) | 101 (92) | 2 (1) | 1 (2) | 88 (111) | 101 (92) | 2 (1) | 1 (2) | 87 (109) | 102 (94) | 2 (1) | 1 (2) | 87 (108) | 102 (95) | 2 (1) | 1 (2) |
|  | **2050** | 77 (90) | 114 (113) | 3 (3) | 0 (0) | 78 (90) | 115 (112) | 3 (3) | 0 (0) | 77 (88) | 115 (115) | 3 (3) | 0 (0) | 77 (89) | 115 (114) | 3 (3) | 0 (0) |
|  | **2080** | 65 (74) | 129 (130) | 3 (3) | 0 (0) | 63 (76) | 130 (128) | 3 (3) | 0 (0) | 64 (74) | 128 (130) | 3 (3) | 0 (0) | 67 (75) | 125 (129) | 3 (3) | 0 (0) |
| **Andorra** | **2020** | 70 (78) | 41 (40) | 1 (1) | 0 (0) | 71 (78) | 41 (40) | 1 (1) | 0 (0) | 71 (78) | 39 (41) | 1 (1) | 0 (0) | 71 (78) | 39 (41) | 1 (1) | 0 (0) |
|  | **2050** | 77 (81) | 41 (44) | 1 (1) | 0 (0) | 86 (85) | 33 (37) | 1 (1) | 0 (0) | 84 (83) | 36 (40) | 1 (1) | 0 (0) | 82 (82) | 35 (41) | 1 (1) | 0 (0) |
|  | **2080** | 86 (89) | 37 (38) | 1 (1) | 0 (0) | 79 (81) | 42 (44) | 1 (1) | 0 (0) | 80 (79) | 39 (45) | 1 (1) | 0 (0) | 79 (79) | 42 (46) | 1 (1) | 0 (0) |
| **Austria** | **2020** | 118 (114) | 104 (113) | 3 (4) | 7 (6) | 121 (113) | 103 (113) | 3 (4) | 7 (6) | 118 (112) | 105 (115) | 3 (4) | 7 (6) | 118 (112) | 106 (114) | 3 (4) | 7 (6) |
|  | **2050** | 137 (126) | 89 (100) | 6 (6) | 4 (4) | 140 (125) | 86 (101) | 6 (6) | 4 (4) | 138 (123) | 88 (103) | 5 (5) | 5 (5) | 139 (124) | 87 (103) | 6 (5) | 4 (5) |
|  | **2080** | 125 (113) | 102 (114) | 5 (4) | 5 (6) | 130 (118) | 98 (109) | 5 (4) | 5 (6) | 131 (117) | 95 (110) | 4 (4) | 6 (6) | 130 (115) | 96 (112) | 4 (4) | 6 (6) |
| **Belgium** | **2020** | 55 (60) | 116 (122) | 2 (2) | 1 (2) | 54 (55) | 118 (127) | 2 (2) | 1 (2) | 53 (56) | 119 (126) | 2 (2) | 1 (2) | 52 (50) | 119 (133) | 1 (2) | 2 (2) |
|  | **2050** | 73 (75) | 97 (108) | 2 (1) | 2 (3) | 73 (73) | 97 (109) | 2 (1) | 2 (3) | 73 (74) | 98 (109) | 1 (1) | 3 (3) | 67 (69) | 105 (114) | 2 (2) | 2 (2) |
|  | **2080** | 56 (57) | 120 (126) | 1 (1) | 3 (3) | 57 (60) | 116 (123) | 1 (1) | 3 (3) | 60 (59) | 113 (124) | 0 (1) | 3 (3) | 56 (61) | 118 (122) | 0 (1) | 3 (3) |
| **Bosnia and**  **Herzeg** | **2020** | 67 (89) | 117 (118) | 1 (2) | 2 (2) | 65 (92) | 117 (115) | 1 (2) | 2 (2) | 64 (90) | 119 (117) | 1 (2) | 2 (2) | 65 (90) | 117 (117) | 1 (2) | 2 (2) |
|  | **2050** | 70 (88) | 117 (121) | 2 (3) | 1 (1) | 74 (91) | 114 (118) | 2 (3) | 1 (1) | 72 (89) | 115 (119) | 2 (3) | 1 (1) | 72 (89) | 116 (120) | 2 (3) | 1 (1) |
|  | **2080** | 53 (78) | 135 (132) | 3 (3) | 1 (1) | 59 (83) | 128 (127) | 2 (3) | 1 (1) | 57 (86) | 128 (124) | 2 (3) | 1 (1) | 63 (84) | 122 (126) | 2 (3) | 1 (1) |
| **Bulgaria** | **2020** | 133 (116) | 112 (128) | 5 (4) | 4 (5) | 131 (120) | 114 (124) | 5 (5) | 4 (4) | 129 (114) | 116 (130) | 5 (3) | 4 (6) | 129 (115) | 116 (129) | 5 (3) | 4 (6) |
|  | **2050** | 132 (106) | 112 (138) | 8 (6) | 1 (3) | 134 (108) | 111 (136) | 8 (5) | 1 (4) | 135 (110) | 110 (134) | 8 (5) | 1 (4) | 132 (109) | 113 (135) | 8 (5) | 1 (4) |
|  | **2080** | 104 (91) | 141 (153) | 7 (6) | 2 (3) | 118 (97) | 127 (147) | 6 (5) | 3 (4) | 117 (99) | 128 (145) | 4 (5) | 5 (4) | 112 (99) | 133 (145) | 5 (5) | 4 (4) |
| **Croatia** | **2020** | 101 (91) | 132 (147) | 3 (3) | 5 (6) | 104 (91) | 129 (147) | 3 (3) | 5 (6) | 105 (90) | 126 (148) | 2 (3) | 5 (6) | 103 (90) | 127 (148) | 3 (3) | 5 (6) |
|  | **2050** | 104 (88) | 129 (151) | 5 (5) | 3 (4) | 104 (94) | 129 (145) | 5 (5) | 3 (4) | 104 (92) | 128 (147) | 5 (5) | 3 (4) | 101 (85) | 131 (154) | 4 (5) | 4 (4) |
|  | **2080** | 84 (74) | 149 (165) | 5 (4) | 3 (5) | 89 (80) | 143 (159) | 5 (5) | 3 (4) | 94 (84) | 138 (155) | 3 (4) | 5 (5) | 90 (81) | 141 (158) | 3 (4) | 5 (5) |
| **Czech Republic** | **2020** | 105 (100) | 112 (118) | 8 (5) | 1 (4) | 104 (99) | 113 (119) | 7 (5) | 2 (4) | 102 (99) | 113 (119) | 7 (5) | 2 (4) | 101 (99) | 116 (119) | 7 (5) | 2 (4) |
|  | **2050** | 98 (95) | 118 (123) | 7 (6) | 2 (3) | 97 (95) | 120 (123) | 7 (6) | 2 (3) | 93 (90) | 124 (128) | 7 (6) | 2 (3) | 93 (86) | 123 (132) | 7 (5) | 2 (4) |
|  | **2080** | 90 (90) | 128 (128) | 6 (5) | 3 (4) | 98 (89) | 120 (129) | 7 (5) | 2 (4) | 99 (90) | 118 (128) | 6 (5) | 3 (4) | 92 (85) | 125 (133) | 6 (4) | 3 (5) |
| **Denmark** | **2020** | 110 (105) | 63 (72) | 2 (2) | 1 (3) | 111 (105) | 62 (72) | 2 (2) | 1 (3) | 108 (105) | 65 (72) | 2 (2) | 1 (3) | 111 (106) | 63 (71) | 3 (2) | 1 (3) |
|  | **2050** | 107 (110) | 66 (68) | 3 (3) | 1 (2) | 108 (110) | 65 (68) | 3 (3) | 1 (2) | 107 (109) | 66 (69) | 3 (3) | 1 (2) | 104 (107) | 67 (71) | 3 (3) | 1 (2) |
|  | **2080** | 65 (72) | 109 (106) | 1 (2) | 3 (3) | 73 (77) | 101 (101) | 2 (2) | 3 (3) | 78 (79) | 97 (99) | 2 (2) | 3 (3) | 78 (78) | 97 (100) | 2 (2) | 3 (3) |
| **Estonia** | **2020** | 76 (79) | 120 (120) | 2 (2) | 5 (5) | 76 (78) | 121 (121) | 2 (2) | 5 (5) | 77 (77) | 120 (122) | 2 (2) | 5 (5) | 78 (79) | 119 (120) | 2 (2) | 5 (5) |
|  | **2050** | 95 (94) | 102 (105) | 3 (3) | 4 (4) | 94 (92) | 103 (107) | 3 (3) | 4 (4) | 93 (95) | 102 (104) | 3 (3) | 4 (4) | 97 (96) | 100 (103) | 3 (3) | 4 (4) |
|  | **2080** | 79 (80) | 119 (119) | 3 (3) | 4 (4) | 84 (83) | 114 (116) | 4 (3) | 3 (4) | 91 (90) | 107 (109) | 4 (4) | 3 (3) | 92 (93) | 106 (106) | 4 (4) | 3 (3) |
| **Finland** | **2020** | 160 (129) | 53 (91) | 4 (3) | 1 (3) | 160 (128) | 54 (92) | 4 (3) | 1 (3) | 158 (128) | 56 (92) | 4 (4) | 1 (2) | 158 (128) | 56 (92) | 4 (3) | 1 (3) |
|  | **2050** | 147 (119) | 67 (101) | 3 (4) | 2 (2) | 147 (119) | 67 (101) | 3 (4) | 2 (2) | 147 (122) | 67 (98) | 4 (4) | 2 (2) | 146 (119) | 67 (101) | 3 (4) | 2 (2) |
|  | **2080** | 140 (110) | 75 (110) | 4 (4) | 2 (2) | 146 (118) | 69 (102) | 4 (4) | 2 (2) | 146 (123) | 69 (97) | 4 (4) | 2 (2) | 147 (122) | 68 (98) | 4 (4) | 2 (2) |
| **France** | **2020** | 96 (72) | 154 (182) | 2 (2) | 3 (4) | 98 (72) | 153 (182) | 3 (2) | 3 (4) | 94 (69) | 156 (185) | 2 (2) | 3 (4) | 93 (68) | 157 (186) | 2 (2) | 3 (4) |
|  | **2050** | 114 (95) | 137 (160) | 3 (3) | 3 (3) | 116 (99) | 135 (156) | 3 (3) | 3 (3) | 118 (98) | 134 (157) | 3 (3) | 3 (3) | 115 (98) | 137 (157) | 3 (3) | 3 (3) |
|  | **2080** | 94 (80) | 157 (175) | 3 (3) | 3 (3) | 103 (84) | 147 (171) | 3 (3) | 3 (3) | 105 (86) | 145 (169) | 3 (3) | 3 (3) | 103 (83) | 147 (172) | 3 (3) | 3 (3) |
| **Germany** | **2020** | 80 (78) | 150 (153) | 3 (2) | 5 (6) | 77 (78) | 153 (153) | 3 (2) | 5 (6) | 76 (77) | 154 (154) | 3 (2) | 5 (6) | 75 (76) | 155 (155) | 3 (2) | 5 (6) |
|  | **2050** | 89 (89) | 142 (142) | 3 (3) | 5 (5) | 86 (86) | 142 (145) | 3 (3) | 5 (5) | 86 (84) | 143 (147) | 2 (3) | 6 (5) | 86 (85) | 142 (146) | 3 (3) | 5 (5) |
|  | **2080** | 90 (84) | 143 (149) | 4 (4) | 4 (4) | 93 (88) | 140 (145) | 4 (4) | 4 (4) | 104 (96) | 129 (137) | 3 (3) | 5 (5) | 101 (92) | 132 (141) | 4 (4) | 4 (4) |
| **Greece** | **2020** | 85 (67) | 138 (173) | 4 (6) | 1 (2) | 87 (71) | 136 (169) | 4 (6) | 1 (2) | 83 (69) | 141 (171) | 4 (6) | 1 (2) | 87 (72) | 137 (168) | 4 (6) | 1 (2) |
|  | **2050** | 91 (75) | 138 (165) | 4 (6) | 1 (2) | 89 (77) | 139 (164) | 4 (6) | 1 (2) | 88 (75) | 140 (165) | 4 (6) | 1 (2) | 89 (76) | 138 (164) | 4 (6) | 1 (2) |
|  | **2080** | 66 (60) | 164 (181) | 4 (5) | 1 (3) | 71 (61) | 159 (180) | 4 (5) | 1 (3) | 72 (53) | 157 (188) | 4 (5) | 1 (3) | 76 (61) | 153 (180) | 4 (5) | 1 (3) |
| **Hungary** | **2020** | 69 (64) | 138 (144) | 6 (5) | 5 (6) | 69 (64) | 137 (144) | 6 (5) | 5 (6) | 68 (64) | 138 (144) | 6 (3) | 5 (8) | 69 (67) | 136 (141) | 6 (3) | 5 (8) |
|  | **2050** | 75 (66) | 132 (142) | 4 (4) | 7 (7) | 78 (69) | 128 (139) | 5 (4) | 6 (7) | 79 (69) | 127 (139) | 4 (4) | 7 (7) | 76 (71) | 128 (137) | 3 (4) | 8 (7) |
|  | **2080** | 57 (57) | 150 (151) | 4 (4) | 7 (7) | 60 (61) | 147 (147) | 4 (4) | 7 (7) | 69 (67) | 138 (141) | 4 (2) | 7 (9) | 68 (70) | 139 (138) | 4 (3) | 7 (8) |
| **Ireland** | **2020** | 43 (48) | 74 (81) | 0 (0) | 2 (3) | 44 (48) | 73 (81) | 0 (0) | 2 (3) | 45 (48) | 73 (81) | 0 (0) | 2 (3) | 43 (48) | 74 (81) | 0 (0) | 2 (3) |
|  | **2050** | 46 (49) | 74 (80) | 0 (0) | 2 (3) | 48 (50) | 72 (79) | 0 (0) | 2 (3) | 46 (50) | 75 (79) | 0 (0) | 2 (3) | 46 (50) | 75 (79) | 0 (0) | 2 (3) |
|  | **2080** | 46 (47) | 78 (82) | 0 (0) | 2 (3) | 47 (49) | 76 (80) | 0 (0) | 2 (3) | 50 (49) | 72 (80) | 0 (0) | 2 (3) | 48 (49) | 72 (80) | 0 (0) | 2 (3) |
| **Italy** | **2020** | 119 (69) | 114 (167) | 4 (1) | 2 (5) | 118 (67) | 114 (169) | 4 (1) | 2 (5) | 120 (67) | 113 (169) | 4 (1) | 2 (5) | 117 (66) | 116 (170) | 4 (1) | 2 (5) |
|  | **2050** | 125 (71) | 108 (165) | 4 (3) | 2 (3) | 124 (71) | 109 (165) | 4 (3) | 2 (3) | 125 (69) | 108 (167) | 4 (3) | 2 (3) | 125 (71) | 107 (165) | 4 (3) | 2 (3) |
|  | **2080** | 105 (62) | 128 (173) | 4 (2) | 2 (4) | 106 (64) | 126 (172) | 4 (2) | 2 (4) | 110 (61) | 123 (174) | 5 (2) | 1 (4) | 108 (59) | 124 (176) | 4 (2) | 2 (4) |
| **Latvia** | **2020** | 77 (82) | 120 (117) | 2 (2) | 8 (8) | 78 (84) | 119 (115) | 2 (3) | 8 (7) | 78 (85) | 119 (114) | 2 (3) | 8 (7) | 77 (84) | 120 (115) | 2 (2) | 8 (8) |
|  | **2050** | 96 (99) | 101 (100) | 4 (4) | 6 (6) | 94 (99) | 103 (100) | 4 (4) | 6 (6) | 99 (101) | 98 (98) | 4 (5) | 6 (5) | 99 (99) | 98 (100) | 4 (4) | 6 (6) |
|  | **2080** | 77 (81) | 120 (118) | 3 (4) | 7 (6) | 81 (83) | 115 (116) | 3 (3) | 7 (7) | 88 (88) | 109 (111) | 4 (4) | 6 (6) | 88 (91) | 109 (108) | 3 (4) | 7 (6) |
| **Liechten-stein** | **2020** | 64 (68) | 59 (60) | 1 (1) | 0 (0) | 64 (68) | 58 (59) | 1 (1) | 0 (0) | 64 (69) | 58 (60) | 1 (1) | 0 (0) | 64 (69) | 58 (59) | 1 (1) | 0 (0) |
|  | **2050** | 73 (79) | 51 (54) | 1 (1) | 0 (0) | 85 (89) | 41 (45) | 1 (1) | 0 (0) | 85 (89) | 40 (45) | 1 (1) | 0 (0) | 85 (88) | 43 (44) | 1 (1) | 0 (0) |
|  | **2080** | 75 (79) | 53 (57) | 0 (0) | 1 (1) | 66 (68) | 65 (66) | 0 (0) | 1 (1) | 81 (88) | 44 (47) | 1 (1) | 0 (0) | 83 (89) | 43 (46) | 1 (1) | 0 (0) |
| **Lithuania** | **2020** | 86 (87) | 99 (105) | 2 (3) | 7 (7) | 86 (87) | 100 (105) | 2 (3) | 7 (7) | 88 (89) | 98 (103) | 2 (3) | 7 (7) | 86 (89) | 100 (103) | 2 (3) | 7 (7) |
|  | **2050** | 68 (67) | 121 (125) | 4 (4) | 6 (6) | 71 (68) | 119 (124) | 4 (4) | 6 (6) | 70 (68) | 119 (124) | 4 (4) | 6 (6) | 68 (67) | 122 (125) | 4 (4) | 6 (6) |
|  | **2080** | 57 (59) | 134 (134) | 3 (3) | 7 (7) | 61 (62) | 130 (131) | 4 (3) | 6 (7) | 68 (71) | 122 (121) | 3 (2) | 7 (8) | 63 (65) | 128 (128) | 4 (3) | 6 (7) |
| **Luxembourg** | **2020** | 58 (67) | 76 (86) | 1 (1) | 1 (2) | 57 (65) | 75 (88) | 1 (1) | 1 (2) | 53 (64) | 78 (88) | 1 (1) | 1 (2) | 54 (63) | 78 (89) | 1 (1) | 1 (2) |
|  | **2050** | 74 (86) | 63 (68) | 0 (1) | 2 (2) | 81 (86) | 54 (68) | 0 (1) | 2 (2) | 73 (83) | 61 (69) | 0 (1) | 2 (2) | 69 (75) | 70 (78) | 0 (1) | 2 (2) |
|  | **2080** | 48 (51) | 93 (103) | 0 (1) | 2 (2) | 55 (57) | 81 (96) | 0 (1) | 2 (2) | 60 (65) | 76 (89) | 0 (1) | 2 (2) | 56 (60) | 79 (94) | 0 (1) | 2 (2) |
| **Macedonia** | **2020** | 128 (114) | 67 (103) | 3 (4) | 0 (1) | 134 (117) | 62 (103) | 3 (4) | 0 (1) | 131 (115) | 64 (104) | 3 (4) | 0 (1) | 132 (116) | 62 (103) | 3 (4) | 0 (1) |
|  | **2050** | 108 (96) | 90 (125) | 3 (4) | 1 (1) | 108 (96) | 90 (124) | 3 (4) | 1 (1) | 111 (96) | 88 (124) | 3 (4) | 1 (1) | 107 (98) | 91 (123) | 3 (4) | 1 (1) |
|  | **2080** | 95 (81) | 106 (140) | 3 (3) | 1 (2) | 97 (83) | 101 (138) | 3 (3) | 1 (2) | 107 (86) | 92 (135) | 3 (3) | 1 (2) | 103 (88) | 96 (133) | 3 (3) | 1 (2) |
| **Malta** | **2020** | 7 (7) | 5 (12) | 1 (1) | 0 (0) | 7 (7) | 6 (12) | 1 (1) | 0 (0) | 7 (7) | 6 (11) | 1 (1) | 0 (0) | 6 (6) | 7 (13) | 1 (1) | 0 (0) |
|  | **2050** | 8 (11) | 8 (8) | 1 (1) | 0 (0) | 8 (11) | 7 (8) | 1 (1) | 0 (0) | 6 (11) | 7 (7) | 1 (1) | 0 (0) | 9 (12) | 7 (8) | 1 (1) | 0 (0) |
|  | **2080** | 8 (9) | 10 (11) | 1 (1) | 0 (0) | 8 (9) | 10 (11) | 1 (1) | 0 (0) | 7 (9) | 10 (11) | 1 (1) | 0 (0) | 8 (8) | 9 (12) | 1 (1) | 0 (0) |
| **Monaco** | **2020** | 3 (33) | 6 (51) | 0 (0) | 0 (0) | 3 (32) | 6 (50) | 0 (0) | 0 (0) | 3 (32) | 6 (52) | 0 (0) | 0 (0) | 3 (32) | 6 (51) | 0 (0) | 0 (0) |
|  | **2050** | 6 (33) | 6 (56) | 0 (0) | 0 (0) | 6 (32) | 6 (59) | 0 (0) | 0 (0) | 5 (32) | 6 (58) | 0 (0) | 0 (0) | 6 (34) | 6 (55) | 0 (0) | 0 (0) |
|  | **2080** | 3 (18) | 24 (71) | 0 (0) | 0 (0) | 2 (29) | 16 (58) | 0 (0) | 0 (0) | 3 (25) | 12 (63) | 0 (0) | 0 (0) | 3 (32) | 9 (56) | 0 (0) | 0 (0) |
| **Montenegro** | **2020** | 84 (90) | 76 (91) | 3 (2) | 0 (2) | 85 (91) | 75 (90) | 3 (2) | 0 (2) | 82 (85) | 75 (94) | 3 (2) | 0 (2) | 83 (85) | 77 (94) | 3 (2) | 0 (2) |
|  | **2050** | 79 (79) | 82 (102) | 2 (2) | 1 (2) | 79 (80) | 81 (101) | 2 (2) | 1 (2) | 76 (77) | 85 (104) | 2 (2) | 1 (2) | 78 (75) | 83 (105) | 2 (2) | 1 (2) |
|  | **2080** | 69 (70) | 93 (112) | 2 (2) | 1 (2) | 70 (71) | 90 (111) | 2 (2) | 1 (2) | 75 (67) | 86 (114) | 2 (2) | 1 (2) | 74 (66) | 87 (115) | 2 (2) | 1 (2) |
| **Netherlands** | **2020** | 37 (37) | 146 (147) | 0 (0) | 4 (4) | 37 (37) | 146 (147) | 0 (0) | 4 (4) | 37 (38) | 146 (146) | 0 (0) | 4 (4) | 37 (37) | 146 (147) | 0 (0) | 4 (4) |
|  | **2050** | 68 (64) | 115 (120) | 0 (0) | 3 (4) | 63 (63) | 119 (121) | 0 (0) | 3 (4) | 63 (64) | 121 (120) | 0 (0) | 4 (4) | 65 (64) | 118 (120) | 0 (1) | 3 (3) |
|  | **2080** | 60 (61) | 124 (123) | 1 (1) | 3 (3) | 62 (63) | 121 (121) | 1 (1) | 3 (3) | 61 (61) | 122 (123) | 0 (0) | 4 (4) | 61 (63) | 121 (121) | 0 (0) | 4 (4) |
| **Norway** | **2020** | 118 (116) | 87 (97) | 2 (2) | 2 (3) | 119 (116) | 87 (97) | 2 (2) | 2 (3) | 120 (120) | 86 (93) | 2 (2) | 2 (3) | 124 (122) | 82 (91) | 2 (2) | 2 (3) |
|  | **2050** | 143 (127) | 65 (86) | 3 (3) | 1 (2) | 142 (125) | 65 (88) | 3 (3) | 1 (2) | 142 (131) | 66 (82) | 2 (3) | 2 (2) | 141 (128) | 66 (85) | 2 (3) | 2 (2) |
|  | **2080** | 136 (122) | 70 (91) | 2 (3) | 2 (2) | 137 (122) | 69 (91) | 2 (3) | 2 (2) | 143 (132) | 65 (81) | 2 (3) | 2 (2) | 142 (125) | 65 (88) | 2 (3) | 2 (2) |
| **Poland** | **2020** | 81 (78) | 138 (148) | 3 (3) | 7 (9) | 81 (77) | 139 (149) | 3 (3) | 7 (9) | 81 (80) | 139 (146) | 3 (3) | 7 (9) | 79 (79) | 140 (147) | 3 (3) | 7 (9) |
|  | **2050** | 77 (74) | 144 (152) | 5 (5) | 6 (7) | 74 (77) | 147 (149) | 3 (4) | 7 (8) | 74 (79) | 147 (147) | 3 (3) | 7 (9) | 74 (73) | 147 (153) | 3 (4) | 7 (8) |
|  | **2080** | 75 (70) | 146 (157) | 5 (6) | 5 (6) | 81 (79) | 141 (148) | 5 (6) | 5 (6) | 80 (84) | 141 (143) | 5 (5) | 5 (7) | 79 (82) | 142 (145) | 5 (5) | 5 (7) |
| **Portugal** | **2020** | 59 (53) | 125 (142) | 1 (0) | 4 (5) | 55 (52) | 130 (142) | 1 (0) | 4 (5) | 55 (53) | 131 (141) | 1 (0) | 4 (5) | 58 (53) | 127 (140) | 1 (0) | 4 (5) |
|  | **2050** | 61 (59) | 127 (136) | 4 (2) | 1 (3) | 65 (59) | 124 (136) | 4 (2) | 1 (3) | 63 (61) | 124 (134) | 4 (2) | 1 (3) | 64 (58) | 124 (136) | 4 (2) | 1 (3) |
|  | **2080** | 56 (58) | 133 (137) | 3 (2) | 2 (3) | 58 (55) | 131 (139) | 3 (2) | 2 (3) | 58 (55) | 131 (140) | 3 (2) | 2 (3) | 57 (53) | 132 (142) | 3 (1) | 2 (4) |
| **Romania** | **2020** | 119 (111) | 124 (136) | 2 (5) | 8 (6) | 122 (109) | 121 (138) | 2 (5) | 8 (6) | 118 (113) | 125 (134) | 2 (5) | 8 (6) | 124 (112) | 119 (135) | 2 (5) | 8 (6) |
|  | **2050** | 92 (86) | 150 (161) | 4 (6) | 5 (5) | 96 (85) | 147 (162) | 4 (6) | 6 (5) | 92 (83) | 151 (163) | 3 (6) | 7 (5) | 95 (84) | 148 (163) | 4 (5) | 6 (6) |
|  | **2080** | 87 (76) | 156 (171) | 3 (5) | 7 (6) | 93 (82) | 150 (165) | 2 (5) | 8 (6) | 98 (86) | 145 (161) | 2 (3) | 8 (8) | 101 (91) | 142 (156) | 2 (4) | 8 (7) |
| **San Marino** | **2020** | 58 (59) | 63 (89) | 0 (1) | 0 (0) | 58 (59) | 63 (89) | 0 (1) | 0 (0) | 58 (59) | 65 (88) | 0 (1) | 0 (0) | 57 (59) | 64 (89) | 0 (1) | 0 (0) |
|  | **2050** | 53 (51) | 76 (97) | 0 (1) | 0 (0) | 56 (52) | 72 (96) | 0 (1) | 0 (0) | 53 (50) | 74 (98) | 0 (1) | 0 (0) | 52 (51) | 75 (97) | 0 (1) | 0 (0) |
|  | **2080** | 36 (38) | 94 (110) | 0 (1) | 0 (0) | 40 (41) | 89 (107) | 0 (1) | 0 (0) | 39 (44) | 90 (104) | 0 (1) | 0 (0) | 43 (45) | 85 (103) | 0 (1) | 0 (0) |
| **Serbia** | **2020** | 113 (100) | 105 (129) | 2 (2) | 8 (8) | 110 (98) | 109 (131) | 2 (2) | 8 (8) | 110 (103) | 109 (126) | 2 (2) | 8 (8) | 112 (101) | 104 (128) | 2 (2) | 8 (8) |
|  | **2050** | 93 (92) | 127 (139) | 3 (3) | 7 (7) | 95 (92) | 125 (139) | 3 (3) | 7 (7) | 93 (88) | 127 (143) | 3 (3) | 7 (7) | 94 (92) | 126 (139) | 3 (3) | 7 (7) |
|  | **2080** | 70 (75) | 150 (156) | 3 (3) | 7 (7) | 80 (80) | 139 (151) | 3 (3) | 7 (7) | 81 (83) | 137 (148) | 1 (2) | 9 (8) | 87 (83) | 133 (148) | 3 (2) | 7 (8) |
| **Slovakia** | **2020** | 128 (90) | 82 (127) | 7 (7) | 3 (3) | 127 (90) | 83 (127) | 7 (7) | 3 (3) | 127 (91) | 84 (126) | 6 (7) | 4 (3) | 125 (92) | 85 (125) | 6 (7) | 4 (3) |
|  | **2050** | 118 (84) | 94 (133) | 7 (6) | 3 (4) | 115 (85) | 97 (132) | 7 (6) | 3 (4) | 114 (81) | 98 (136) | 7 (5) | 3 (5) | 111 (80) | 101 (137) | 7 (5) | 3 (5) |
|  | **2080** | 102 (71) | 111 (146) | 4 (4) | 6 (6) | 107 (81) | 106 (136) | 5 (5) | 5 (5) | 104 (79) | 108 (138) | 4 (3) | 6 (7) | 103 (80) | 110 (137) | 4 (3) | 6 (7) |
| **Slovenia** | **2020** | 113 (99) | 88 (115) | 4 (4) | 1 (1) | 113 (100) | 87 (114) | 4 (4) | 1 (1) | 117 (96) | 85 (118) | 4 (4) | 1 (1) | 113 (96) | 88 (118) | 4 (4) | 1 (1) |
|  | **2050** | 127 (115) | 76 (101) | 5 (4) | 0 (1) | 130 (120) | 73 (96) | 5 (4) | 0 (1) | 122 (114) | 80 (102) | 5 (4) | 0 (1) | 124 (114) | 79 (102) | 5 (4) | 0 (1) |
|  | **2080** | 114 (95) | 90 (121) | 4 (4) | 1 (1) | 117 (102) | 85 (114) | 5 (4) | 0 (1) | 119 (103) | 85 (113) | 5 (4) | 0 (1) | 118 (103) | 86 (113) | 5 (4) | 0 (1) |
| **Spain** | **2020** | 102 (59) | 131 (178) | 5 (3) | 2 (5) | 106 (63) | 127 (174) | 5 (3) | 2 (5) | 104 (59) | 129 (178) | 5 (2) | 2 (6) | 101 (60) | 132 (177) | 5 (3) | 2 (5) |
|  | **2050** | 107 (68) | 125 (170) | 4 (3) | 3 (5) | 110 (68) | 122 (170) | 4 (3) | 3 (5) | 105 (67) | 127 (171) | 4 (3) | 3 (5) | 105 (70) | 127 (168) | 4 (4) | 3 (4) |
|  | **2080** | 80 (58) | 152 (180) | 4 (1) | 3 (7) | 86 (61) | 146 (177) | 4 (1) | 3 (7) | 94 (62) | 138 (176) | 4 (1) | 3 (7) | 91 (63) | 141 (175) | 4 (2) | 3 (6) |
| **Sweden** | **2020** | 134 (111) | 89 (115) | 2 (3) | 4 (3) | 134 (109) | 90 (117) | 2 (3) | 4 (3) | 135 (112) | 88 (114) | 2 (3) | 4 (3) | 140 (111) | 84 (115) | 2 (3) | 4 (3) |
|  | **2050** | 143 (119) | 79 (106) | 3 (4) | 3 (2) | 144 (121) | 79 (104) | 3 (4) | 3 (2) | 145 (121) | 77 (104) | 3 (4) | 3 (2) | 143 (120) | 80 (105) | 3 (4) | 3 (2) |
|  | **2080** | 124 (102) | 100 (124) | 2 (2) | 4 (4) | 130 (109) | 94 (117) | 2 (2) | 4 (4) | 141 (117) | 83 (109) | 3 (4) | 3 (2) | 137 (114) | 87 (112) | 3 (3) | 3 (3) |
| **Switzerland** | **2020** | 127 (121) | 70 (76) | 3 (2) | 0 (2) | 124 (122) | 73 (76) | 2 (2) | 1 (2) | 125 (121) | 73 (77) | 2 (2) | 1 (2) | 125 (120) | 73 (78) | 2 (2) | 1 (2) |
|  | **2050** | 137 (130) | 61 (69) | 2 (3) | 2 (1) | 140 (131) | 57 (67) | 2 (3) | 1 (1) | 144 (132) | 54 (67) | 3 (3) | 1 (1) | 140 (131) | 58 (68) | 2 (2) | 2 (2) |
|  | **2080** | 120 (112) | 80 (89) | 2 (2) | 2 (2) | 122 (118) | 78 (83) | 2 (2) | 2 (2) | 121 (120) | 76 (82) | 1 (2) | 2 (2) | 123 (119) | 75 (83) | 1 (2) | 3 (2) |
| **UK** | **2020** | 72 (70) | 117 (123) | 2 (1) | 2 (3) | 71 (69) | 118 (124) | 2 (1) | 2 (3) | 72 (68) | 117 (125) | 2 (1) | 2 (3) | 71 (70) | 118 (123) | 2 (1) | 2 (3) |
|  | **2050** | 82 (78) | 108 (115) | 1 (1) | 3 (3) | 78 (75) | 112 (116) | 1 (1) | 3 (3) | 79 (77) | 110 (115) | 1 (1) | 3 (3) | 80 (78) | 110 (115) | 1 (1) | 3 (3) |
|  | **2080** | 71 (69) | 120 (124) | 1 (1) | 3 (3) | 73 (72) | 117 (121) | 1 (1) | 3 (3) | 77 (76) | 112 (117) | 1 (1) | 3 (3) | 76 (74) | 113 (119) | 1 (1) | 3 (3) |

| ***Mammals*** | |  | |  | |  | |  | |  | |  | |  |  | |  | |  | |  | |  | |  | |  |  | |  | |
| --- | --- | --- | --- | --- | --- | --- | --- | --- | --- | --- | --- | --- | --- | --- | --- | --- | --- | --- | --- | --- | --- | --- | --- | --- | --- | --- | --- | --- | --- | --- | --- |
| **Albania** | **2020** | | 32 (37) | 39 (36) | 5 (5) | | 2 (2) | | 30 (37) | | 41 (36) | | 4 (5) | | 3 (2) | 30 (36) | | 41 (37) | | 4 (5) | | 3 (2) | | 30 (35) | | 41 (38) | | | 4 (5) | | 3 (2) |
|  | **2050** | | 24 (28) | 47 (45) | 4 (5) | | 3 (2) | | 27 (28) | | 46 (45) | | 4 (4) | | 3 (3) | 26 (27) | | 47 (46) | | 4 (4) | | 3 (3) | | 25 (28) | | 46 (45) | | | 4 (4) | | 3 (3) |
|  | **2080** | | 20 (21) | 52 (52) | 4 (4) | | 3 (3) | | 20 (21) | | 52 (52) | | 4 (4) | | 3 (3) | 20 (23) | | 53 (50) | | 3 (3) | | 4 (4) | | 20 (21) | | 53 (52) | | | 3 (3) | | 4 (4) |
| **Andorra** | **2020** | | 41 (43) | 7 (9) | 5 (6) | | 0 (0) | | 39 (41) | | 8 (11) | | 4 (5) | | 1 (1) | 39 (41) | | 8 (10) | | 4 (5) | | 1 (1) | | 40 (41) | | 8 (11) | | | 4 (5) | | 0 (1) |
|  | **2050** | | 35 (36) | 16 (17) | 5 (4) | | 1 (2) | | 44 (40) | | 6 (13) | | 6 (5) | | 0 (1) | 39 (39) | | 12 (14) | | 4 (5) | | 1 (1) | | 44 (39) | | 7 (13) | | | 6 (5) | | 0 (1) |
|  | **2080** | | 38 (39) | 14 (14) | 5 (5) | | 1 (1) | | 35 (37) | | 17 (16) | | 4 (5) | | 2 (1) | 34 (35) | | 17 (18) | | 3 (4) | | 2 (2) | | 36 (36) | | 16 (17) | | | 4 (5) | | 1 (1) |
| **Austria** | **2020** | | 57 (48) | 29 (39) | 5 (4) | | 3 (4) | | 57 (50) | | 29 (37) | | 5 (4) | | 3 (4) | 58 (50) | | 28 (37) | | 5 (4) | | 3 (4) | | 58 (50) | | 28 (37) | | | 5 (4) | | 3 (4) |
|  | **2050** | | 56 (49) | 31 (37) | 6 (5) | | 2 (3) | | 59 (50) | | 28 (36) | | 7 (5) | | 1 (3) | 59 (49) | | 28 (38) | | 6 (5) | | 2 (3) | | 60 (49) | | 27 (37) | | | 6 (5) | | 2 (3) |
|  | **2080** | | 53 (39) | 33 (48) | 5 (5) | | 3 (3) | | 54 (44) | | 32 (43) | | 5 (5) | | 3 (3) | 55 (45) | | 31 (42) | | 5 (4) | | 3 (4) | | 55 (45) | | 31 (42) | | | 4 (4) | | 4 (4) |
| **Belgium** | **2020** | | 35 (31) | 28 (32) | 4 (3) | | 4 (5) | | 35 (30) | | 28 (33) | | 4 (3) | | 4 (5) | 35 (31) | | 28 (32) | | 5 (3) | | 3 (5) | | 33 (30) | | 29 (33) | | | 4 (3) | | 3 (5) |
|  | **2050** | | 34 (22) | 29 (41) | 5 (3) | | 3 (5) | | 32 (25) | | 31 (38) | | 4 (5) | | 4 (3) | 32 (25) | | 30 (38) | | 4 (5) | | 4 (3) | | 29 (22) | | 34 (41) | | | 4 (4) | | 4 (4) |
|  | **2080** | | 19 (16) | 44 (47) | 4 (5) | | 4 (3) | | 23 (19) | | 40 (44) | | 4 (5) | | 4 (3) | 23 (22) | | 39 (41) | | 4 (5) | | 4 (3) | | 20 (19) | | 43 (44) | | | 4 (4) | | 4 (4) |
| **Bosnia and**  **Herzeg** | **2020** | | 23 (36) | 42 (41) | 4 (8) | | 1 (1) | | 25 (35) | | 41 (42) | | 4 (7) | | 1 (2) | 24 (36) | | 41 (41) | | 4 (8) | | 1 (1) | | 25 (36) | | 42 (41) | | | 4 (8) | | 1 (1) |
|  | **2050** | | 24 (37) | 43 (40) | 3 (7) | | 2 (2) | | 27 (37) | | 42 (40) | | 2 (7) | | 3 (2) | 26 (37) | | 43 (40) | | 3 (7) | | 2 (2) | | 27 (37) | | 42 (40) | | | 3 (7) | | 2 (2) |
|  | **2080** | | 20 (34) | 49 (43) | 2 (7) | | 3 (2) | | 20 (35) | | 48 (42) | | 2 (7) | | 3 (2) | 22 (36) | | 46 (41) | | 3 (7) | | 2 (2) | | 23 (34) | | 46 (43) | | | 3 (7) | | 2 (2) |
| **Bulgaria** | **2020** | | 59 (46) | 26 (40) | 11 (12) | | 1 (0) | | 58 (46) | | 28 (40) | | 10 (12) | | 2 (0) | 59 (46) | | 27 (40) | | 11 (12) | | 1 (0) | | 59 (48) | | 27 (38) | | | 11 (12) | | 1 (0) |
|  | **2050** | | 47 (33) | 39 (53) | 10 (10) | | 2 (2) | | 47 (35) | | 39 (51) | | 10 (10) | | 2 (2) | 50 (35) | | 36 (50) | | 10 (10) | | 2 (2) | | 50 (35) | | 36 (51) | | | 10 (10) | | 2 (2) |
|  | **2080** | | 47 (29) | 39 (57) | 10 (7) | | 2 (5) | | 50 (35) | | 36 (51) | | 10 (8) | | 2 (4) | 48 (33) | | 38 (53) | | 9 (7) | | 3 (5) | | 49 (37) | | 37 (49) | | | 10 (7) | | 2 (5) |
| **Croatia** | **2020** | | 35 (31) | 48 (57) | 7 (5) | | 4 (7) | | 34 (31) | | 49 (57) | | 6 (5) | | 5 (7) | 36 (31) | | 47 (57) | | 8 (5) | | 3 (7) | | 33 (30) | | 50 (58) | | | 6 (5) | | 5 (7) |
|  | **2050** | | 38 (32) | 45 (56) | 8 (7) | | 4 (5) | | 38 (32) | | 45 (55) | | 8 (7) | | 4 (5) | 36 (31) | | 47 (57) | | 8 (6) | | 4 (6) | | 37 (31) | | 47 (57) | | | 8 (7) | | 4 (5) |
|  | **2080** | | 31 (25) | 52 (63) | 7 (5) | | 4 (7) | | 33 (26) | | 50 (62) | | 7 (5) | | 4 (7) | 32 (27) | | 53 (61) | | 6 (6) | | 6 (6) | | 33 (25) | | 50 (63) | | | 7 (5) | | 4 (7) |
| **Czech Republic** | **2020** | | 55 (43) | 25 (36) | 6 (4) | | 3 (5) | | 53 (43) | | 27 (37) | | 6 (4) | | 3 (5) | 53 (44) | | 27 (36) | | 6 (4) | | 3 (5) | | 50 (43) | | 30 (37) | | | 6 (4) | | 3 (5) |
|  | **2050** | | 43 (36) | 37 (44) | 6 (4) | | 3 (5) | | 42 (39) | | 38 (41) | | 6 (4) | | 3 (5) | 40 (37) | | 40 (43) | | 4 (4) | | 5 (5) | | 40 (39) | | 39 (41) | | | 4 (4) | | 5 (5) |
|  | **2080** | | 37 (31) | 43 (49) | 4 (5) | | 5 (4) | | 42 (34) | | 38 (46) | | 4 (4) | | 5 (5) | 48 (44) | | 32 (36) | | 4 (4) | | 5 (5) | | 48 (42) | | 32 (38) | | | 4 (4) | | 5 (5) |
| **Denmark** | **2020** | | 37 (39) | 14 (14) | 3 (4) | | 0 (0) | | 39 (39) | | 14 (14) | | 4 (4) | | 0 (0) | 38 (39) | | 14 (14) | | 3 (4) | | 0 (0) | | 38 (39) | | 14 (14) | | | 3 (4) | | 0 (0) |
|  | **2050** | | 35 (35) | 18 (18) | 4 (3) | | 0 (1) | | 35 (35) | | 18 (18) | | 4 (3) | | 0 (1) | 35 (34) | | 18 (19) | | 4 (3) | | 0 (1) | | 35 (33) | | 18 (20) | | | 4 (3) | | 0 (1) |
|  | **2080** | | 23 (22) | 30 (31) | 3 (2) | | 1 (2) | | 26 (25) | | 27 (28) | | 2 (2) | | 2 (2) | 27 (28) | | 26 (25) | | 2 (2) | | 2 (2) | | 26 (26) | | 26 (27) | | | 2 (2) | | 2 (2) |
| **Estonia** | **2020** | | 26 (26) | 28 (28) | 1 (1) | | 4 (4) | | 26 (26) | | 28 (28) | | 1 (1) | | 4 (4) | 26 (26) | | 28 (28) | | 1 (1) | | 4 (4) | | 26 (26) | | 28 (28) | | | 1 (1) | | 4 (4) |
|  | **2050** | | 27 (27) | 27 (27) | 1 (1) | | 4 (4) | | 26 (27) | | 28 (27) | | 0 (1) | | 5 (4) | 27 (27) | | 27 (27) | | 0 (1) | | 5 (4) | | 27 (26) | | 27 (28) | | | 0 (0) | | 5 (5) |
|  | **2080** | | 19 (20) | 35 (34) | 1 (1) | | 4 (4) | | 21 (22) | | 33 (32) | | 1 (1) | | 4 (4) | 23 (24) | | 31 (30) | | 1 (1) | | 4 (4) | | 22 (22) | | 32 (32) | | | 1 (1) | | 4 (4) |
| **Finland** | **2020** | | 40 (31) | 16 (27) | 3 (2) | | 2 (3) | | 39 (31) | | 17 (27) | | 3 (2) | | 2 (3) | 39 (31) | | 17 (27) | | 3 (2) | | 2 (3) | | 39 (31) | | 17 (27) | | | 3 (2) | | 2 (3) |
|  | **2050** | | 40 (30) | 16 (28) | 2 (2) | | 3 (3) | | 40 (31) | | 16 (27) | | 2 (2) | | 3 (3) | 40 (31) | | 16 (27) | | 2 (2) | | 3 (3) | | 40 (31) | | 16 (27) | | | 2 (2) | | 3 (3) |
|  | **2080** | | 33 (27) | 23 (31) | 2 (2) | | 3 (3) | | 35 (28) | | 21 (30) | | 2 (2) | | 3 (3) | 35 (30) | | 21 (28) | | 2 (2) | | 3 (3) | | 35 (30) | | 21 (28) | | | 2 (2) | | 3 (3) |
| **France** | **2020** | | 34 (25) | 58 (68) | 7 (6) | | 6 (7) | | 34 (28) | | 58 (65) | | 7 (7) | | 6 (6) | 36 (27) | | 56 (66) | | 8 (6) | | 5 (7) | | 35 (28) | | 57 (65) | | | 7 (7) | | 6 (6) |
|  | **2050** | | 36 (20) | 56 (72) | 7 (2) | | 6 (11) | | 36 (21) | | 56 (71) | | 7 (2) | | 6 (11) | 38 (22) | | 54 (70) | | 7 (3) | | 6 (10) | | 38 (22) | | 54 (70) | | | 7 (3) | | 6 (10) |
|  | **2080** | | 30 (18) | 63 (75) | 6 (2) | | 7 (11) | | 34 (19) | | 58 (74) | | 8 (2) | | 5 (11) | 38 (20) | | 54 (72) | | 8 (3) | | 5 (10) | | 38 (21) | | 54 (71) | | | 8 (3) | | 5 (10) |
| **Germany** | **2020** | | 35 (33) | 49 (50) | 4 (4) | | 4 (4) | | 35 (33) | | 49 (50) | | 4 (4) | | 4 (4) | 35 (33) | | 49 (50) | | 4 (4) | | 4 (4) | | 35 (32) | | 49 (51) | | | 4 (4) | | 4 (4) |
|  | **2050** | | 31 (31) | 53 (52) | 4 (4) | | 4 (4) | | 31 (30) | | 53 (53) | | 5 (4) | | 3 (4) | 30 (29) | | 54 (54) | | 5 (4) | | 3 (4) | | 28 (29) | | 56 (54) | | | 4 (4) | | 4 (4) |
|  | **2080** | | 23 (23) | 61 (60) | 3 (3) | | 5 (5) | | 28 (27) | | 55 (55) | | 2 (2) | | 6 (6) | 35 (31) | | 49 (52) | | 3 (2) | | 5 (6) | | 30 (30) | | 54 (53) | | | 2 (2) | | 6 (6) |
| **Greece** | **2020** | | 21 (8) | 54 (70) | 5 (2) | | 5 (9) | | 24 (7) | | 52 (71) | | 5 (2) | | 5 (9) | 22 (8) | | 53 (70) | | 5 (2) | | 5 (9) | | 25 (9) | | 49 (69) | | | 6 (2) | | 4 (9) |
|  | **2050** | | 25 (22) | 52 (56) | 7 (4) | | 4 (7) | | 26 (24) | | 51 (54) | | 7 (5) | | 4 (6) | 27 (23) | | 50 (55) | | 7 (5) | | 4 (6) | | 26 (29) | | 51 (49) | | | 7 (7) | | 4 (4) |
|  | **2080** | | 19 (16) | 58 (62) | 5 (2) | | 6 (9) | | 22 (17) | | 55 (61) | | 5 (3) | | 6 (8) | 22 (17) | | 55 (61) | | 5 (3) | | 6 (8) | | 25 (19) | | 52 (59) | | | 5 (4) | | 6 (7) |
| **Hungary** | **2020** | | 29 (24) | 54 (62) | 5 (5) | | 7 (7) | | 29 (23) | | 54 (63) | | 5 (4) | | 7 (8) | 29 (26) | | 54 (60) | | 5 (4) | | 7 (8) | | 30 (28) | | 53 (58) | | | 5 (5) | | 7 (7) |
|  | **2050** | | 33 (31) | 50 (55) | 6 (5) | | 6 (7) | | 34 (34) | | 51 (52) | | 6 (5) | | 6 (7) | 37 (37) | | 48 (49) | | 7 (5) | | 5 (7) | | 38 (39) | | 47 (47) | | | 7 (5) | | 5 (7) |
|  | **2080** | | 27 (26) | 57 (60) | 7 (5) | | 5 (7) | | 28 (30) | | 55 (56) | | 7 (6) | | 5 (6) | 33 (35) | | 52 (51) | | 6 (6) | | 6 (6) | | 36 (36) | | 49 (50) | | | 7 (6) | | 5 (6) |
| **Ireland** | **2020** | | 14 (19) | 13 (9) | 1 (2) | | 1 (0) | | 14 (19) | | 13 (9) | | 1 (2) | | 1 (0) | 13 (18) | | 14 (10) | | 1 (2) | | 1 (0) | | 13 (18) | | 13 (10) | | | 1 (2) | | 1 (0) |
|  | **2050** | | 14 (15) | 13 (13) | 1 (2) | | 1 (0) | | 15 (15) | | 13 (13) | | 1 (2) | | 1 (0) | 13 (15) | | 15 (13) | | 1 (2) | | 1 (0) | | 13 (15) | | 14 (13) | | | 1 (2) | | 1 (0) |
|  | **2080** | | 11 (11) | 17 (17) | 1 (2) | | 1 (0) | | 13 (12) | | 14 (16) | | 1 (2) | | 1 (0) | 15 (14) | | 13 (14) | | 1 (2) | | 1 (0) | | 15 (14) | | 12 (14) | | | 1 (2) | | 1 (0) |
| **Italy** | **2020** | | 58 (35) | 32 (56) | 5 (4) | | 5 (6) | | 59 (35) | | 31 (55) | | 5 (4) | | 5 (6) | 57 (35) | | 33 (56) | | 5 (4) | | 5 (6) | | 59 (35) | | 31 (56) | | | 5 (4) | | 5 (6) |
|  | **2050** | | 50 (29) | 40 (62) | 3 (3) | | 7 (7) | | 52 (28) | | 38 (63) | | 3 (3) | | 7 (7) | 52 (27) | | 38 (64) | | 3 (3) | | 7 (7) | | 53 (31) | | 37 (60) | | | 3 (3) | | 7 (7) |
|  | **2080** | | 42 (21) | 48 (70) | 3 (2) | | 7 (8) | | 42 (23) | | 48 (68) | | 3 (2) | | 7 (8) | 44 (24) | | 46 (67) | | 3 (3) | | 7 (7) | | 46 (25) | | 44 (66) | | | 4 (3) | | 6 (7) |
| **Latvia** | **2020** | | 30 (29) | 30 (32) | 2 (2) | | 3 (4) | | 30 (29) | | 30 (32) | | 2 (2) | | 3 (4) | 30 (29) | | 30 (32) | | 2 (2) | | 3 (4) | | 30 (30) | | 30 (31) | | | 2 (2) | | 3 (4) |
|  | **2050** | | 33 (33) | 27 (28) | 2 (2) | | 3 (4) | | 32 (33) | | 28 (28) | | 2 (2) | | 3 (4) | 32 (33) | | 28 (28) | | 2 (2) | | 3 (4) | | 32 (34) | | 28 (27) | | | 2 (2) | | 3 (4) |
|  | **2080** | | 24 (24) | 36 (37) | 2 (2) | | 3 (4) | | 24 (23) | | 36 (38) | | 2 (2) | | 3 (4) | 29 (25) | | 31 (36) | | 2 (2) | | 3 (4) | | 26 (24) | | 34 (37) | | | 2 (2) | | 3 (4) |
| **Liechten-stein** | **2020** | | 28 (29) | 22 (23) | 3 (3) | | 0 (0) | | 28 (28) | | 22 (23) | | 3 (3) | | 0 (0) | 28 (28) | | 22 (22) | | 3 (3) | | 0 (0) | | 28 (28) | | 23 (23) | | | 3 (3) | | 0 (0) |
|  | **2050** | | 34 (34) | 19 (19) | 1 (1) | | 2 (2) | | 42 (43) | | 10 (10) | | 2 (3) | | 0 (0) | 41 (41) | | 11 (11) | | 2 (2) | | 0 (0) | | 42 (42) | | 11 (11) | | | 3 (3) | | 0 (0) |
|  | **2080** | | 31 (31) | 19 (20) | 2 (2) | | 1 (1) | | 38 (38) | | 15 (15) | | 2 (2) | | 1 (1) | 40 (40) | | 11 (13) | | 3 (3) | | 0 (0) | | 40 (40) | | 12 (12) | | | 3 (3) | | 0 (0) |
| **Lithuania** | **2020** | | 35 (33) | 24 (26) | 2 (2) | | 3 (3) | | 35 (33) | | 24 (26) | | 2 (2) | | 3 (3) | 36 (34) | | 23 (25) | | 2 (2) | | 3 (3) | | 36 (34) | | 23 (25) | | | 2 (2) | | 3 (3) |
|  | **2050** | | 28 (26) | 31 (33) | 3 (2) | | 2 (3) | | 30 (28) | | 29 (31) | | 3 (3) | | 2 (2) | 30 (29) | | 29 (30) | | 3 (3) | | 2 (2) | | 30 (25) | | 29 (34) | | | 3 (2) | | 2 (3) |
|  | **2080** | | 20 (20) | 39 (39) | 2 (2) | | 3 (3) | | 21 (21) | | 38 (38) | | 2 (2) | | 3 (3) | 22 (22) | | 35 (37) | | 2 (2) | | 3 (3) | | 21 (21) | | 37 (38) | | | 2 (2) | | 3 (3) |
| **Luxembourg** | **2020** | | 41 (37) | 18 (24) | 6 (4) | | 1 (3) | | 39 (36) | | 20 (25) | | 5 (4) | | 2 (3) | 40 (37) | | 19 (24) | | 6 (4) | | 1 (3) | | 39 (35) | | 20 (26) | | | 5 (4) | | 2 (3) |
|  | **2050** | | 37 (32) | 21 (29) | 5 (5) | | 2 (2) | | 38 (37) | | 21 (24) | | 5 (5) | | 2 (2) | 37 (37) | | 21 (24) | | 5 (5) | | 2 (2) | | 35 (33) | | 24 (28) | | | 5 (5) | | 2 (2) |
|  | **2080** | | 16 (17) | 42 (44) | 3 (4) | | 4 (3) | | 24 (22) | | 35 (39) | | 3 (4) | | 4 (3) | 31 (27) | | 28 (34) | | 4 (4) | | 3 (3) | | 29 (26) | | 30 (35) | | | 5 (4) | | 2 (3) |
| **Macedonia** | **2020** | | 45 (29) | 30 (48) | 6 (7) | | 3 (2) | | 46 (28) | | 30 (49) | | 7 (7) | | 2 (2) | 45 (30) | | 30 (47) | | 6 (6) | | 3 (3) | | 46 (33) | | 30 (44) | | | 6 (7) | | 3 (2) |
|  | **2050** | | 41 (27) | 35 (50) | 7 (6) | | 2 (3) | | 41 (29) | | 35 (48) | | 7 (6) | | 2 (3) | 43 (30) | | 33 (47) | | 8 (6) | | 1 (3) | | 43 (31) | | 33 (46) | | | 8 (6) | | 1 (3) |
|  | **2080** | | 36 (22) | 40 (55) | 7 (4) | | 2 (5) | | 38 (23) | | 38 (54) | | 7 (5) | | 2 (4) | 42 (25) | | 34 (52) | | 7 (5) | | 2 (4) | | 42 (27) | | 34 (50) | | | 7 (6) | | 2 (3) |
| **Malta** | **2020** | | 4 (7) | 7 (8) | 0 (0) | | 0 (0) | | 4 (6) | | 7 (8) | | 0 (0) | | 0 (0) | 4 (7) | | 7 (8) | | 0 (0) | | 0 (0) | | 4 (6) | | 7 (8) | | | 0 (0) | | 0 (0) |
|  | **2050** | | 8 (10) | 5 (5) | 0 (0) | | 0 (0) | | 8 (10) | | 5 (5) | | 0 (0) | | 0 (0) | 8 (10) | | 5 (5) | | 0 (0) | | 0 (0) | | 8 (10) | | 5 (5) | | | 0 (0) | | 0 (0) |
|  | **2080** | | 8 (10) | 5 (5) | 0 (0) | | 0 (0) | | 6 (8) | | 6 (6) | | 0 (0) | | 0 (0) | 8 (9) | | 5 (5) | | 0 (0) | | 0 (0) | | 9 (9) | | 5 (6) | | | 0 (0) | | 0 (0) |
| **Monaco** | **2020** | | 3 (14) | 0 (6) | 1 (1) | | 0 (0) | | 3 (14) | | 1 (6) | | 1 (1) | | 0 (0) | 3 (14) | | 1 (7) | | 1 (1) | | 0 (0) | | 3 (14) | | 1 (6) | | | 1 (1) | | 0 (0) |
|  | **2050** | | 1 (4) | 2 (16) | 1 (1) | | 0 (0) | | 1 (4) | | 2 (17) | | 1 (1) | | 0 (0) | 1 (4) | | 2 (17) | | 1 (1) | | 0 (0) | | 1 (5) | | 2 (14) | | | 1 (1) | | 0 (0) |
|  | **2080** | | 0 (5) | 5 (15) | 0 (0) | | 0 (1) | | 1 (9) | | 4 (12) | | 1 (1) | | 0 (0) | 1 (9) | | 3 (12) | | 1 (1) | | 0 (0) | | 1 (9) | | 3 (11) | | | 1 (1) | | 0 (0) |
| **Montenegro** | **2020** | | 36 (38) | 25 (26) | 3 (5) | | 2 (0) | | 35 (37) | | 26 (27) | | 3 (4) | | 2 (1) | 35 (35) | | 25 (29) | | 3 (3) | | 2 (2) | | 35 (38) | | 26 (26) | | | 3 (5) | | 2 (0) |
|  | **2050** | | 40 (39) | 22 (25) | 4 (5) | | 1 (0) | | 40 (40) | | 21 (24) | | 5 (5) | | 0 (0) | 40 (39) | | 22 (25) | | 5 (5) | | 0 (0) | | 39 (39) | | 23 (25) | | | 5 (5) | | 0 (0) |
|  | **2080** | | 33 (33) | 29 (31) | 4 (5) | | 1 (0) | | 35 (36) | | 27 (28) | | 4 (5) | | 1 (0) | 36 (36) | | 26 (28) | | 4 (4) | | 1 (1) | | 36 (35) | | 26 (29) | | | 4 (5) | | 1 (0) |
| **Netherlands** | **2020** | | 19 (19) | 41 (41) | 2 (2) | | 5 (5) | | 20 (18) | | 40 (41) | | 3 (2) | | 4 (5) | 20 (19) | | 40 (41) | | 3 (2) | | 4 (5) | | 20 (19) | | 40 (41) | | | 3 (2) | | 4 (5) |
|  | **2050** | | 23 (20) | 37 (40) | 4 (4) | | 3 (3) | | 22 (22) | | 38 (38) | | 5 (5) | | 2 (2) | 23 (23) | | 37 (37) | | 5 (5) | | 2 (2) | | 21 (21) | | 39 (39) | | | 4 (5) | | 3 (2) |
|  | **2080** | | 17 (16) | 43 (44) | 5 (4) | | 2 (3) | | 18 (17) | | 42 (43) | | 5 (4) | | 2 (3) | 22 (21) | | 38 (39) | | 5 (5) | | 2 (2) | | 20 (19) | | 39 (40) | | | 4 (4) | | 2 (2) |
| **Norway** | **2020** | | 23 (27) | 29 (26) | 1 (1) | | 3 (3) | | 23 (27) | | 29 (26) | | 1 (1) | | 3 (3) | 23 (27) | | 29 (26) | | 1 (1) | | 3 (3) | | 23 (29) | | 29 (24) | | | 1 (1) | | 3 (3) |
|  | **2050** | | 35 (34) | 17 (19) | 2 (1) | | 2 (3) | | 35 (34) | | 17 (19) | | 2 (1) | | 2 (3) | 35 (35) | | 17 (18) | | 2 (1) | | 2 (3) | | 35 (35) | | 17 (18) | | | 2 (1) | | 2 (3) |
|  | **2080** | | 33 (31) | 19 (22) | 2 (1) | | 2 (3) | | 33 (32) | | 19 (21) | | 2 (1) | | 2 (3) | 35 (33) | | 17 (20) | | 2 (2) | | 2 (2) | | 35 (34) | | 17 (19) | | | 2 (2) | | 2 (2) |
| **Poland** | **2020** | | 46 (43) | 34 (37) | 5 (5) | | 3 (3) | | 46 (44) | | 34 (36) | | 5 (5) | | 3 (3) | 47 (45) | | 33 (35) | | 5 (5) | | 3 (3) | | 46 (44) | | 34 (36) | | | 5 (5) | | 3 (3) |
|  | **2050** | | 35 (31) | 45 (49) | 4 (4) | | 4 (4) | | 37 (34) | | 43 (46) | | 4 (4) | | 4 (4) | 37 (34) | | 43 (46) | | 4 (4) | | 4 (4) | | 37 (33) | | 43 (47) | | | 4 (4) | | 4 (4) |
|  | **2080** | | 30 (23) | 50 (57) | 4 (4) | | 4 (4) | | 34 (25) | | 46 (55) | | 4 (4) | | 4 (4) | 34 (31) | | 46 (49) | | 4 (4) | | 4 (4) | | 34 (30) | | 46 (50) | | | 4 (4) | | 4 (4) |
| **Portugal** | **2020** | | 20 (12) | 39 (48) | 3 (1) | | 6 (8) | | 19 (12) | | 40 (48) | | 3 (1) | | 6 (8) | 19 (12) | | 40 (48) | | 3 (1) | | 6 (8) | | 20 (13) | | 39 (47) | | | 3 (1) | | 6 (8) |
|  | **2050** | | 9 (5) | 50 (55) | 0 (0) | | 9 (9) | | 8 (6) | | 51 (54) | | 0 (0) | | 9 (9) | 9 (5) | | 50 (55) | | 0 (0) | | 9 (9) | | 9 (7) | | 50 (53) | | | 0 (0) | | 9 (9) |
|  | **2080** | | 8 (8) | 51 (52) | 1 (1) | | 8 (8) | | 8 (8) | | 51 (52) | | 0 (1) | | 9 (8) | 8 (8) | | 51 (52) | | 0 (0) | | 9 (9) | | 8 (10) | | 51 (50) | | | 0 (1) | | 9 (8) |
| **Romania** | **2020** | | 48 (41) | 36 (44) | 8 (11) | | 7 (4) | | 46 (41) | | 38 (44) | | 7 (11) | | 8 (4) | 48 (43) | | 36 (42) | | 9 (11) | | 6 (4) | | 47 (43) | | 37 (42) | | | 8 (11) | | 7 (4) |
|  | **2050** | | 41 (25) | 43 (60) | 10 (8) | | 5 (7) | | 42 (25) | | 42 (60) | | 10 (8) | | 5 (7) | 42 (32) | | 42 (53) | | 10 (9) | | 5 (6) | | 44 (30) | | 40 (55) | | | 10 (9) | | 5 (6) |
|  | **2080** | | 33 (26) | 52 (59) | 8 (9) | | 7 (6) | | 38 (27) | | 47 (58) | | 8 (9) | | 7 (6) | 44 (35) | | 39 (50) | | 8 (10) | | 7 (5) | | 44 (36) | | 40 (49) | | | 9 (10) | | 6 (5) |
| **San Marino** | **2020** | | 16 (27) | 34 (35) | 1 (3) | | 2 (3) | | 16 (28) | | 33 (34) | | 1 (3) | | 1 (3) | 16 (26) | | 33 (36) | | 1 (3) | | 1 (3) | | 18 (28) | | 33 (34) | | | 1 (3) | | 1 (3) |
|  | **2050** | | 15 (23) | 37 (39) | 1 (2) | | 2 (4) | | 15 (23) | | 37 (39) | | 1 (2) | | 2 (4) | 15 (23) | | 37 (39) | | 1 (2) | | 2 (4) | | 16 (23) | | 36 (39) | | | 1 (2) | | 2 (4) |
|  | **2080** | | 14 (15) | 37 (47) | 1 (2) | | 2 (4) | | 14 (17) | | 38 (45) | | 1 (2) | | 2 (4) | 13 (20) | | 38 (42) | | 1 (2) | | 2 (4) | | 14 (21) | | 37 (41) | | | 1 (2) | | 2 (4) |
| **Serbia** | **2020** | | 41 (35) | 45 (52) | 7 (7) | | 3 (3) | | 41 (37) | | 45 (50) | | 7 (7) | | 3 (3) | 40 (37) | | 46 (50) | | 7 (7) | | 3 (3) | | 42 (36) | | 44 (51) | | | 7 (7) | | 3 (3) |
|  | **2050** | | 42 (35) | 44 (52) | 7 (7) | | 3 (3) | | 43 (35) | | 43 (52) | | 7 (7) | | 3 (3) | 42 (37) | | 44 (50) | | 7 (7) | | 3 (3) | | 42 (38) | | 44 (49) | | | 7 (7) | | 3 (3) |
|  | **2080** | | 30 (31) | 56 (56) | 5 (6) | | 5 (4) | | 31 (34) | | 54 (53) | | 5 (7) | | 5 (3) | 37 (34) | | 49 (52) | | 5 (5) | | 5 (5) | | 36 (35) | | 49 (52) | | | 5 (5) | | 5 (5) |
| **Slovakia** | **2020** | | 68 (44) | 16 (47) | 9 (7) | | 2 (5) | | 67 (44) | | 17 (47) | | 9 (7) | | 2 (5) | 66 (46) | | 18 (45) | | 9 (7) | | 2 (5) | | 66 (46) | | 18 (45) | | | 9 (7) | | 2 (5) |
|  | **2050** | | 63 (35) | 23 (56) | 10 (7) | | 2 (5) | | 65 (37) | | 23 (54) | | 10 (7) | | 2 (5) | 65 (39) | | 21 (52) | | 10 (7) | | 2 (5) | | 64 (39) | | 23 (52) | | | 10 (7) | | 2 (5) |
|  | **2080** | | 56 (31) | 31 (60) | 10 (8) | | 2 (4) | | 56 (36) | | 30 (55) | | 10 (8) | | 2 (4) | 62 (37) | | 24 (54) | | 10 (7) | | 2 (5) | | 62 (39) | | 25 (52) | | | 10 (8) | | 2 (4) |
| **Slovenia** | **2020** | | 50 (32) | 26 (47) | 6 (5) | | 0 (2) | | 51 (32) | | 25 (47) | | 6 (5) | | 0 (2) | 47 (32) | | 28 (47) | | 6 (5) | | 0 (2) | | 48 (31) | | 27 (48) | | | 6 (5) | | 0 (2) |
|  | **2050** | | 57 (34) | 20 (45) | 6 (4) | | 0 (3) | | 57 (35) | | 20 (44) | | 6 (4) | | 0 (3) | 56 (35) | | 21 (44) | | 6 (4) | | 0 (3) | | 56 (35) | | 21 (44) | | | 6 (4) | | 0 (3) |
|  | **2080** | | 48 (30) | 30 (49) | 6 (4) | | 1 (3) | | 54 (32) | | 24 (47) | | 6 (4) | | 1 (3) | 54 (33) | | 22 (46) | | 6 (4) | | 0 (3) | | 54 (33) | | 24 (46) | | | 6 (4) | | 1 (3) |
| **Spain** | **2020** | | 37 (13) | 42 (66) | 9 (4) | | 3 (8) | | 37 (13) | | 42 (66) | | 9 (4) | | 3 (8) | 35 (13) | | 44 (66) | | 9 (4) | | 3 (8) | | 37 (13) | | 42 (66) | | | 9 (4) | | 3 (8) |
|  | **2050** | | 29 (13) | 50 (66) | 7 (3) | | 5 (9) | | 33 (12) | | 46 (67) | | 7 (2) | | 5 (10) | 32 (11) | | 47 (68) | | 7 (2) | | 5 (10) | | 33 (13) | | 46 (66) | | | 7 (3) | | 5 (9) |
|  | **2080** | | 20 (12) | 59 (67) | 3 (2) | | 9 (10) | | 22 (11) | | 57 (68) | | 5 (2) | | 7 (10) | 27 (12) | | 52 (67) | | 7 (2) | | 5 (10) | | 28 (14) | | 51 (65) | | | 7 (2) | | 5 (10) |
| **Sweden** | **2020** | | 39 (36) | 25 (28) | 4 (4) | | 3 (3) | | 39 (36) | | 25 (28) | | 4 (4) | | 3 (3) | 40 (36) | | 24 (28) | | 4 (4) | | 3 (3) | | 40 (36) | | 24 (28) | | | 4 (4) | | 3 (3) |
|  | **2050** | | 43 (39) | 21 (25) | 5 (4) | | 2 (3) | | 43 (40) | | 21 (24) | | 5 (4) | | 2 (3) | 43 (42) | | 21 (22) | | 5 (5) | | 2 (2) | | 43 (39) | | 21 (25) | | | 5 (4) | | 2 (3) |
|  | **2080** | | 40 (34) | 24 (30) | 5 (2) | | 2 (5) | | 41 (37) | | 23 (27) | | 5 (4) | | 2 (3) | 44 (38) | | 20 (26) | | 6 (4) | | 1 (3) | | 42 (38) | | 22 (26) | | | 5 (4) | | 2 (3) |
| **Switzerland** | **2020** | | 55 (50) | 24 (28) | 6 (5) | | 2 (2) | | 55 (50) | | 24 (29) | | 6 (6) | | 2 (2) | 55 (51) | | 24 (28) | | 6 (6) | | 2 (2) | | 54 (51) | | 25 (28) | | | 6 (6) | | 2 (2) |
|  | **2050** | | 58 (50) | 21 (29) | 6 (5) | | 2 (3) | | 61 (55) | | 18 (24) | | 6 (6) | | 2 (2) | 61 (57) | | 18 (22) | | 6 (7) | | 2 (1) | | 62 (54) | | 17 (25) | | | 6 (5) | | 2 (3) |
|  | **2080** | | 50 (40) | 28 (39) | 5 (5) | | 3 (3) | | 52 (43) | | 27 (36) | | 5 (5) | | 3 (3) | 52 (46) | | 27 (33) | | 5 (5) | | 3 (3) | | 51 (45) | | 28 (34) | | | 5 (5) | | 3 (3) |
| **UK** | **2020** | | 21 (18) | 26 (30) | 2 (2) | | 1 (1) | | 20 (18) | | 27 (29) | | 2 (2) | | 1 (1) | 20 (19) | | 27 (29) | | 2 (2) | | 1 (1) | | 21 (19) | | 26 (29) | | | 2 (2) | | 1 (1) |
|  | **2050** | | 21 (19) | 27 (29) | 2 (2) | | 1 (1) | | 21 (20) | | 27 (28) | | 2 (2) | | 1 (1) | 21 (20) | | 27 (28) | | 2 (2) | | 1 (1) | | 21 (20) | | 27 (28) | | | 2 (2) | | 1 (1) |
|  | **2080** | | 15 (14) | 33 (34) | 1 (1) | | 2 (2) | | 18 (15) | | 30 (33) | | 3 (1) | | 0 (2) | 21 (19) | | 27 (29) | | 3 (3) | | 0 (0) | | 20 (19) | | 28 (29) | | | 3 (3) | | 0 (0) |

| ***Plants*** | |  |  |  |  |  |  |  |  |  |  |  |  |  |  |  |  |
| --- | --- | --- | --- | --- | --- | --- | --- | --- | --- | --- | --- | --- | --- | --- | --- | --- | --- |
| **Albania** | **2020** | 265 (269) | 355 (381) | 1 (1) | 1 (2) | 266 (273) | 356 (376) | 1 (1) | 1 (2) | 270 (262) | 356 (387) | 1 (1) | 1 (2) | 267 (272) | 357 (377) | 1 (1) | 1 (2) |
|  | **2050** | 329 (300) | 297 (349) | 3 (2) | 0 (1) | 332 (313) | 295 (337) | 3 (2) | 0 (1) | 328 (307) | 297 (342) | 3 (2) | 0 (1) | 330 (312) | 298 (338) | 3 (2) | 0 (1) |
|  | **2080** | 229 (204) | 399 (445) | 1 (1) | 1 (2) | 248 (223) | 380 (426) | 1 (1) | 1 (2) | 244 (214) | 385 (436) | 1 (1) | 1 (2) | 260 (225) | 368 (425) | 1 (1) | 1 (2) |
| **Andorra** | **2020** | 198 (223) | 118 (127) | 0 (0) | 0 (0) | 197 (222) | 122 (129) | 0 (0) | 0 (0) | 200 (227) | 118 (121) | 0 (0) | 0 (0) | 198 (222) | 120 (124) | 0 (0) | 0 (0) |
|  | **2050** | 186 (192) | 144 (162) | 0 (0) | 0 (0) | 216 (214) | 111 (135) | 0 (0) | 0 (0) | 209 (214) | 117 (136) | 0 (0) | 0 (0) | 213 (213) | 116 (139) | 0 (0) | 0 (0) |
|  | **2080** | 208 (206) | 140 (151) | 0 (0) | 0 (0) | 200 (205) | 144 (152) | 0 (0) | 0 (0) | 187 (194) | 153 (159) | 0 (0) | 0 (0) | 191 (196) | 151 (158) | 0 (0) | 0 (0) |
| **Austria** | **2020** | 350 (302) | 300 (356) | 0 (0) | 0 (0) | 348 (302) | 303 (356) | 0 (0) | 0 (0) | 348 (300) | 301 (358) | 0 (0) | 0 (0) | 347 (297) | 304 (360) | 0 (0) | 0 (0) |
|  | **2050** | 392 (318) | 261 (341) | 0 (0) | 0 (0) | 396 (327) | 257 (331) | 0 (0) | 0 (0) | 394 (318) | 257 (339) | 0 (0) | 0 (0) | 390 (321) | 264 (336) | 0 (0) | 0 (0) |
|  | **2080** | 303 (242) | 352 (419) | 0 (0) | 0 (0) | 331 (272) | 326 (388) | 0 (0) | 0 (0) | 340 (285) | 313 (375) | 0 (0) | 0 (0) | 333 (282) | 320 (378) | 0 (0) | 0 (0) |
| **Belgium** | **2020** | 131 (118) | 240 (271) | 0 (0) | 0 (0) | 130 (116) | 240 (273) | 0 (0) | 0 (0) | 131 (117) | 245 (272) | 0 (0) | 0 (0) | 129 (114) | 243 (275) | 0 (0) | 0 (0) |
|  | **2050** | 147 (139) | 227 (252) | 0 (0) | 0 (0) | 137 (133) | 235 (258) | 0 (0) | 0 (0) | 145 (132) | 227 (258) | 0 (0) | 0 (0) | 140 (125) | 235 (266) | 0 (0) | 0 (0) |
|  | **2080** | 127 (125) | 253 (266) | 0 (0) | 0 (0) | 141 (133) | 240 (258) | 0 (0) | 0 (0) | 146 (129) | 234 (261) | 0 (0) | 0 (0) | 138 (130) | 240 (261) | 0 (0) | 0 (0) |
| **Bosnia and**  **Herzeg** | **2020** | 232 (337) | 296 (293) | 1 (1) | 0 (0) | 237 (343) | 292 (288) | 1 (1) | 0 (0) | 233 (330) | 299 (301) | 1 (1) | 0 (0) | 237 (340) | 296 (291) | 1 (1) | 0 (0) |
|  | **2050** | 271 (335) | 276 (296) | 1 (1) | 0 (0) | 274 (344) | 266 (286) | 1 (1) | 0 (0) | 268 (334) | 275 (297) | 1 (1) | 0 (0) | 275 (336) | 269 (294) | 1 (1) | 0 (0) |
|  | **2080** | 194 (275) | 359 (356) | 1 (1) | 0 (0) | 215 (293) | 335 (338) | 1 (1) | 0 (0) | 211 (304) | 333 (327) | 1 (1) | 0 (0) | 222 (305) | 323 (326) | 1 (1) | 0 (0) |
| **Bulgaria** | **2020** | 385 (276) | 262 (384) | 1 (1) | 0 (0) | 381 (276) | 269 (384) | 1 (1) | 0 (0) | 372 (265) | 275 (394) | 1 (1) | 0 (0) | 377 (279) | 269 (381) | 1 (1) | 0 (0) |
|  | **2050** | 336 (229) | 316 (431) | 1 (1) | 0 (0) | 335 (234) | 315 (426) | 1 (1) | 0 (0) | 337 (236) | 312 (424) | 1 (1) | 0 (0) | 340 (243) | 311 (417) | 1 (1) | 0 (0) |
|  | **2080** | 320 (170) | 334 (490) | 1 (0) | 0 (1) | 341 (195) | 313 (465) | 1 (0) | 0 (1) | 310 (190) | 344 (470) | 1 (0) | 0 (1) | 334 (206) | 320 (454) | 1 (0) | 0 (1) |
| **Croatia** | **2020** | 292 (265) | 343 (406) | 1 (1) | 0 (0) | 298 (269) | 338 (401) | 1 (1) | 0 (0) | 285 (257) | 350 (414) | 1 (1) | 0 (0) | 287 (261) | 347 (410) | 1 (1) | 0 (0) |
|  | **2050** | 317 (287) | 325 (384) | 1 (1) | 0 (0) | 329 (291) | 317 (379) | 1 (1) | 0 (0) | 319 (284) | 323 (386) | 1 (1) | 0 (0) | 318 (287) | 327 (384) | 1 (1) | 0 (0) |
|  | **2080** | 252 (191) | 398 (480) | 1 (1) | 0 (0) | 267 (212) | 380 (458) | 1 (1) | 0 (0) | 266 (211) | 380 (460) | 1 (1) | 0 (0) | 264 (214) | 381 (457) | 1 (1) | 0 (0) |
| **Czech Republic** | **2020** | 304 (263) | 214 (258) | 0 (0) | 0 (0) | 304 (262) | 214 (259) | 0 (0) | 0 (0) | 302 (270) | 215 (251) | 0 (0) | 0 (0) | 299 (269) | 219 (252) | 0 (0) | 0 (0) |
|  | **2050** | 272 (241) | 247 (279) | 0 (0) | 0 (0) | 283 (251) | 235 (269) | 0 (0) | 0 (0) | 275 (247) | 243 (273) | 0 (0) | 0 (0) | 272 (246) | 247 (273) | 0 (0) | 0 (0) |
|  | **2080** | 221 (200) | 298 (320) | 0 (0) | 0 (0) | 232 (217) | 286 (303) | 0 (0) | 0 (0) | 252 (235) | 266 (286) | 0 (0) | 0 (0) | 247 (232) | 272 (289) | 0 (0) | 0 (0) |
| **Denmark** | **2020** | 186 (186) | 133 (141) | 0 (0) | 0 (0) | 183 (185) | 139 (142) | 0 (0) | 0 (0) | 182 (184) | 138 (143) | 0 (0) | 0 (0) | 183 (185) | 138 (143) | 0 (0) | 0 (0) |
|  | **2050** | 135 (139) | 190 (189) | 0 (0) | 0 (0) | 134 (145) | 189 (183) | 0 (0) | 0 (0) | 132 (141) | 191 (187) | 0 (0) | 0 (0) | 123 (130) | 201 (198) | 0 (0) | 0 (0) |
|  | **2080** | 78 (83) | 247 (245) | 0 (0) | 0 (0) | 84 (89) | 240 (239) | 0 (0) | 0 (0) | 96 (100) | 230 (228) | 0 (0) | 0 (0) | 91 (94) | 235 (234) | 0 (0) | 0 (0) |
| **Estonia** | **2020** | 216 (216) | 97 (98) | 0 (0) | 0 (0) | 213 (213) | 101 (101) | 0 (0) | 0 (0) | 208 (210) | 106 (104) | 0 (0) | 0 (0) | 211 (212) | 103 (102) | 0 (0) | 0 (0) |
|  | **2050** | 181 (181) | 132 (133) | 0 (0) | 0 (0) | 182 (181) | 132 (133) | 0 (0) | 0 (0) | 177 (178) | 137 (135) | 0 (0) | 0 (0) | 180 (182) | 134 (132) | 0 (0) | 0 (0) |
|  | **2080** | 136 (137) | 178 (177) | 0 (0) | 0 (0) | 147 (148) | 167 (166) | 0 (0) | 0 (0) | 159 (158) | 155 (156) | 0 (0) | 0 (0) | 151 (154) | 163 (160) | 0 (0) | 0 (0) |
| **Finland** | **2020** | 249 (223) | 69 (108) | 0 (0) | 0 (0) | 249 (223) | 69 (108) | 0 (0) | 0 (0) | 246 (221) | 72 (110) | 0 (0) | 0 (0) | 248 (224) | 70 (107) | 0 (0) | 0 (0) |
|  | **2050** | 228 (196) | 91 (135) | 0 (0) | 0 (0) | 228 (198) | 91 (133) | 0 (0) | 0 (0) | 226 (195) | 94 (136) | 0 (0) | 0 (0) | 225 (195) | 95 (136) | 0 (0) | 0 (0) |
|  | **2080** | 209 (171) | 110 (160) | 0 (0) | 0 (0) | 212 (179) | 107 (152) | 0 (0) | 0 (0) | 219 (185) | 101 (146) | 0 (0) | 0 (0) | 214 (183) | 105 (148) | 0 (0) | 0 (0) |
| **France** | **2020** | 372 (310) | 511 (578) | 0 (0) | 0 (0) | 378 (309) | 504 (580) | 0 (0) | 0 (0) | 379 (299) | 505 (589) | 0 (0) | 0 (0) | 370 (303) | 513 (586) | 0 (0) | 0 (0) |
|  | **2050** | 344 (270) | 542 (618) | 0 (0) | 0 (0) | 349 (275) | 535 (612) | 0 (0) | 0 (0) | 342 (268) | 540 (618) | 0 (0) | 0 (0) | 348 (266) | 534 (621) | 0 (0) | 0 (0) |
|  | **2080** | 335 (244) | 555 (649) | 0 (0) | 0 (0) | 350 (261) | 537 (630) | 0 (0) | 0 (0) | 360 (274) | 522 (616) | 0 (0) | 0 (0) | 358 (273) | 528 (617) | 0 (0) | 0 (0) |
| **Germany** | **2020** | 197 (189) | 418 (426) | 0 (0) | 0 (0) | 195 (189) | 420 (426) | 0 (0) | 0 (0) | 195 (187) | 420 (428) | 0 (0) | 0 (0) | 192 (189) | 423 (426) | 0 (0) | 0 (0) |
|  | **2050** | 190 (183) | 424 (433) | 0 (0) | 0 (0) | 193 (183) | 422 (433) | 0 (0) | 0 (0) | 193 (180) | 422 (434) | 0 (0) | 0 (0) | 192 (183) | 424 (433) | 0 (0) | 0 (0) |
|  | **2080** | 183 (176) | 432 (440) | 0 (0) | 0 (0) | 189 (182) | 425 (433) | 0 (0) | 0 (0) | 199 (187) | 417 (428) | 0 (0) | 0 (0) | 191 (189) | 423 (426) | 0 (0) | 0 (0) |
| **Greece** | **2020** | 187 (175) | 474 (547) | 0 (0) | 3 (3) | 191 (178) | 472 (544) | 0 (0) | 3 (3) | 180 (178) | 475 (544) | 0 (0) | 3 (3) | 189 (182) | 470 (540) | 0 (0) | 3 (3) |
|  | **2050** | 272 (247) | 398 (475) | 2 (2) | 1 (1) | 275 (256) | 395 (465) | 2 (2) | 1 (1) | 270 (248) | 399 (474) | 2 (2) | 1 (1) | 283 (266) | 388 (455) | 2 (2) | 1 (1) |
|  | **2080** | 205 (207) | 473 (515) | 2 (1) | 1 (2) | 215 (204) | 463 (518) | 2 (1) | 1 (2) | 223 (203) | 451 (519) | 2 (1) | 1 (2) | 242 (212) | 433 (510) | 2 (1) | 1 (2) |
| **Hungary** | **2020** | 174 (159) | 333 (370) | 0 (0) | 0 (0) | 178 (159) | 331 (370) | 0 (0) | 0 (0) | 180 (165) | 328 (363) | 0 (0) | 0 (0) | 186 (176) | 323 (352) | 0 (0) | 0 (0) |
|  | **2050** | 143 (137) | 367 (392) | 0 (0) | 0 (0) | 149 (140) | 361 (388) | 0 (0) | 0 (0) | 170 (162) | 341 (367) | 0 (0) | 0 (0) | 175 (168) | 334 (361) | 0 (0) | 0 (0) |
|  | **2080** | 94 (93) | 420 (436) | 0 (0) | 0 (0) | 111 (108) | 402 (421) | 0 (0) | 0 (0) | 133 (137) | 377 (392) | 0 (0) | 0 (0) | 136 (141) | 374 (388) | 0 (0) | 0 (0) |
| **Ireland** | **2020** | 132 (111) | 133 (166) | 0 (0) | 0 (0) | 131 (111) | 134 (166) | 0 (0) | 0 (0) | 130 (110) | 137 (167) | 0 (0) | 0 (0) | 129 (111) | 139 (166) | 0 (0) | 0 (0) |
|  | **2050** | 106 (76) | 164 (201) | 0 (0) | 0 (0) | 110 (79) | 160 (198) | 0 (0) | 0 (0) | 107 (77) | 162 (200) | 0 (0) | 0 (0) | 107 (77) | 164 (200) | 0 (0) | 0 (0) |
|  | **2080** | 87 (62) | 181 (215) | 0 (0) | 0 (0) | 94 (69) | 177 (208) | 0 (0) | 0 (0) | 101 (75) | 170 (202) | 0 (0) | 0 (0) | 102 (73) | 169 (204) | 0 (0) | 0 (0) |
| **Italy** | **2020** | 569 (317) | 324 (609) | 0 (1) | 0 (0) | 564 (318) | 325 (608) | 0 (1) | 0 (0) | 564 (303) | 325 (623) | 0 (1) | 0 (0) | 566 (317) | 323 (609) | 0 (1) | 0 (0) |
|  | **2050** | 482 (293) | 421 (633) | 0 (1) | 0 (0) | 487 (298) | 413 (629) | 0 (1) | 0 (0) | 483 (293) | 420 (634) | 0 (1) | 0 (0) | 490 (297) | 411 (629) | 0 (1) | 0 (0) |
|  | **2080** | 430 (267) | 470 (659) | 0 (1) | 0 (0) | 449 (272) | 450 (655) | 0 (1) | 0 (0) | 461 (263) | 440 (664) | 0 (1) | 0 (0) | 478 (276) | 424 (651) | 0 (1) | 0 (0) |
| **Latvia** | **2020** | 204 (214) | 125 (117) | 0 (0) | 0 (0) | 206 (215) | 124 (116) | 0 (0) | 0 (0) | 207 (214) | 123 (117) | 0 (0) | 0 (0) | 206 (215) | 124 (116) | 0 (0) | 0 (0) |
|  | **2050** | 185 (189) | 146 (142) | 0 (0) | 0 (0) | 188 (192) | 143 (139) | 0 (0) | 0 (0) | 182 (182) | 149 (149) | 0 (0) | 0 (0) | 187 (188) | 144 (143) | 0 (0) | 0 (0) |
|  | **2080** | 139 (141) | 192 (190) | 0 (0) | 0 (0) | 153 (149) | 178 (182) | 0 (0) | 0 (0) | 166 (163) | 163 (168) | 0 (0) | 0 (0) | 164 (167) | 167 (164) | 0 (0) | 0 (0) |
| **Liechten-stein** | **2020** | 192 (198) | 181 (183) | 0 (0) | 0 (0) | 191 (200) | 181 (181) | 0 (0) | 0 (0) | 194 (199) | 180 (182) | 0 (0) | 0 (0) | 189 (198) | 181 (182) | 0 (0) | 0 (0) |
|  | **2050** | 220 (225) | 152 (161) | 0 (0) | 0 (0) | 241 (245) | 132 (138) | 0 (0) | 0 (0) | 240 (242) | 134 (140) | 0 (0) | 0 (0) | 237 (242) | 135 (141) | 0 (0) | 0 (0) |
|  | **2080** | 191 (193) | 187 (191) | 0 (0) | 0 (0) | 211 (216) | 170 (172) | 0 (0) | 0 (0) | 230 (233) | 143 (150) | 0 (0) | 0 (0) | 223 (226) | 156 (159) | 0 (0) | 0 (0) |
| **Lithuania** | **2020** | 225 (219) | 107 (118) | 0 (0) | 0 (0) | 224 (221) | 108 (116) | 0 (0) | 0 (0) | 222 (220) | 109 (117) | 0 (0) | 0 (0) | 224 (221) | 108 (116) | 0 (0) | 0 (0) |
|  | **2050** | 173 (168) | 160 (169) | 0 (0) | 0 (0) | 179 (178) | 153 (159) | 0 (0) | 0 (0) | 177 (174) | 155 (163) | 0 (0) | 0 (0) | 173 (171) | 159 (166) | 0 (0) | 0 (0) |
|  | **2080** | 133 (136) | 203 (201) | 0 (0) | 0 (0) | 142 (142) | 192 (195) | 0 (0) | 0 (0) | 161 (158) | 173 (179) | 0 (0) | 0 (0) | 152 (153) | 182 (184) | 0 (0) | 0 (0) |
| **Luxembourg** | **2020** | 128 (138) | 169 (191) | 0 (0) | 0 (0) | 122 (133) | 173 (196) | 0 (0) | 0 (0) | 120 (133) | 181 (196) | 0 (0) | 0 (0) | 120 (131) | 179 (197) | 0 (0) | 0 (0) |
|  | **2050** | 115 (120) | 191 (209) | 0 (0) | 0 (0) | 127 (128) | 181 (200) | 0 (0) | 0 (0) | 124 (131) | 181 (198) | 0 (0) | 0 (0) | 124 (123) | 186 (206) | 0 (0) | 0 (0) |
|  | **2080** | 81 (79) | 229 (250) | 0 (0) | 0 (0) | 95 (95) | 216 (234) | 0 (0) | 0 (0) | 99 (105) | 208 (224) | 0 (0) | 0 (0) | 98 (98) | 211 (231) | 0 (0) | 0 (0) |
| **Macedonia** | **2020** | 281 (203) | 283 (415) | 0 (0) | 1 (1) | 285 (216) | 278 (400) | 0 (0) | 1 (1) | 284 (216) | 280 (401) | 0 (0) | 1 (1) | 289 (230) | 280 (387) | 0 (0) | 1 (1) |
|  | **2050** | 317 (247) | 268 (371) | 1 (0) | 0 (1) | 319 (252) | 264 (367) | 1 (0) | 0 (1) | 324 (250) | 257 (369) | 1 (0) | 0 (1) | 326 (259) | 261 (359) | 1 (0) | 0 (1) |
|  | **2080** | 220 (173) | 371 (446) | 0 (0) | 1 (1) | 254 (201) | 333 (418) | 0 (0) | 1 (1) | 271 (201) | 315 (418) | 0 (0) | 1 (1) | 280 (214) | 306 (405) | 0 (0) | 1 (1) |
| **Malta** | **2020** | 97 (125) | 44 (56) | 0 (0) | 0 (0) | 98 (124) | 45 (56) | 0 (0) | 0 (0) | 99 (125) | 43 (56) | 0 (0) | 0 (0) | 94 (123) | 44 (57) | 0 (0) | 0 (0) |
|  | **2050** | 120 (136) | 45 (46) | 0 (0) | 0 (0) | 119 (135) | 46 (47) | 0 (0) | 0 (0) | 122 (135) | 43 (47) | 0 (0) | 0 (0) | 120 (134) | 45 (48) | 0 (0) | 0 (0) |
|  | **2080** | 117 (122) | 52 (59) | 0 (0) | 0 (0) | 117 (121) | 51 (62) | 0 (0) | 0 (0) | 119 (125) | 46 (54) | 0 (0) | 0 (0) | 124 (131) | 43 (51) | 0 (0) | 0 (0) |
| **Monaco** | **2020** | 19 (139) | 23 (138) | 0 (0) | 0 (0) | 19 (144) | 26 (134) | 0 (0) | 0 (0) | 20 (135) | 26 (143) | 0 (0) | 0 (0) | 20 (144) | 26 (134) | 0 (0) | 0 (0) |
|  | **2050** | 17 (96) | 49 (184) | 0 (0) | 0 (0) | 17 (98) | 49 (185) | 0 (0) | 0 (0) | 17 (98) | 45 (183) | 0 (0) | 0 (0) | 17 (100) | 49 (182) | 0 (0) | 0 (0) |
|  | **2080** | 27 (89) | 81 (192) | 0 (0) | 0 (0) | 27 (96) | 70 (186) | 0 (0) | 0 (0) | 24 (102) | 56 (178) | 0 (0) | 0 (0) | 28 (108) | 55 (175) | 0 (0) | 0 (0) |
| **Montenegro** | **2020** | 336 (331) | 199 (227) | 2 (1) | 0 (1) | 337 (331) | 201 (230) | 2 (1) | 0 (1) | 328 (324) | 209 (237) | 2 (1) | 0 (1) | 331 (329) | 207 (231) | 2 (1) | 0 (1) |
|  | **2050** | 355 (343) | 194 (218) | 2 (2) | 0 (0) | 355 (347) | 193 (214) | 2 (2) | 0 (0) | 348 (346) | 200 (215) | 2 (2) | 0 (0) | 353 (344) | 195 (217) | 2 (2) | 0 (0) |
|  | **2080** | 295 (267) | 252 (294) | 2 (2) | 0 (0) | 315 (294) | 233 (267) | 2 (2) | 0 (0) | 315 (295) | 232 (266) | 2 (2) | 0 (0) | 318 (298) | 229 (263) | 2 (2) | 0 (0) |
| **Netherlands** | **2020** | 102 (98) | 255 (259) | 0 (0) | 0 (0) | 101 (97) | 255 (261) | 0 (0) | 0 (0) | 99 (100) | 258 (258) | 0 (0) | 0 (0) | 98 (98) | 259 (260) | 0 (0) | 0 (0) |
|  | **2050** | 103 (105) | 251 (253) | 0 (0) | 0 (0) | 101 (99) | 256 (259) | 0 (0) | 0 (0) | 105 (105) | 252 (253) | 0 (0) | 0 (0) | 102 (102) | 255 (256) | 0 (0) | 0 (0) |
|  | **2080** | 113 (112) | 245 (246) | 0 (0) | 0 (0) | 114 (111) | 244 (247) | 0 (0) | 0 (0) | 106 (107) | 249 (251) | 0 (0) | 0 (0) | 107 (107) | 247 (251) | 0 (0) | 0 (0) |
| **Norway** | **2020** | 258 (249) | 91 (119) | 0 (0) | 0 (0) | 256 (249) | 93 (119) | 0 (0) | 0 (0) | 256 (251) | 94 (117) | 0 (0) | 0 (0) | 255 (252) | 95 (116) | 0 (0) | 0 (0) |
|  | **2050** | 256 (244) | 95 (124) | 0 (0) | 0 (0) | 258 (245) | 93 (123) | 0 (0) | 0 (0) | 255 (244) | 94 (124) | 0 (0) | 0 (0) | 256 (243) | 95 (125) | 0 (0) | 0 (0) |
|  | **2080** | 243 (212) | 109 (156) | 0 (0) | 0 (0) | 245 (226) | 107 (142) | 0 (0) | 0 (0) | 253 (234) | 98 (134) | 0 (0) | 0 (0) | 250 (233) | 101 (135) | 0 (0) | 0 (0) |
| **Poland** | **2020** | 290 (253) | 232 (276) | 0 (0) | 0 (0) | 283 (253) | 240 (276) | 0 (0) | 0 (0) | 287 (256) | 236 (273) | 0 (0) | 0 (0) | 285 (255) | 238 (274) | 0 (0) | 0 (0) |
|  | **2050** | 236 (206) | 288 (323) | 0 (0) | 0 (0) | 238 (209) | 285 (320) | 0 (0) | 0 (0) | 249 (218) | 273 (311) | 0 (0) | 0 (0) | 246 (218) | 277 (311) | 0 (0) | 0 (0) |
|  | **2080** | 209 (190) | 317 (339) | 0 (0) | 0 (0) | 226 (205) | 299 (324) | 0 (0) | 0 (0) | 248 (221) | 275 (308) | 0 (0) | 0 (0) | 242 (214) | 283 (314) | 0 (0) | 0 (0) |
| **Portugal** | **2020** | 223 (200) | 253 (289) | 0 (0) | 0 (0) | 224 (201) | 253 (290) | 0 (0) | 0 (0) | 229 (208) | 244 (282) | 0 (0) | 0 (0) | 232 (211) | 245 (280) | 0 (0) | 0 (0) |
|  | **2050** | 153 (141) | 322 (349) | 0 (0) | 0 (0) | 158 (147) | 315 (343) | 0 (0) | 0 (0) | 160 (149) | 314 (341) | 0 (0) | 0 (0) | 167 (151) | 308 (339) | 0 (0) | 0 (0) |
|  | **2080** | 161 (151) | 319 (340) | 0 (0) | 0 (0) | 163 (152) | 318 (337) | 0 (0) | 0 (0) | 171 (153) | 309 (337) | 0 (0) | 0 (0) | 171 (166) | 309 (324) | 0 (0) | 0 (0) |
| **Romania** | **2020** | 373 (332) | 278 (330) | 0 (0) | 0 (0) | 373 (330) | 278 (332) | 0 (0) | 0 (0) | 370 (343) | 280 (319) | 0 (0) | 0 (0) | 374 (347) | 276 (315) | 0 (0) | 0 (0) |
|  | **2050** | 272 (212) | 377 (450) | 0 (0) | 0 (0) | 281 (216) | 368 (446) | 0 (0) | 0 (0) | 286 (231) | 369 (431) | 0 (0) | 0 (0) | 285 (234) | 367 (428) | 0 (0) | 0 (0) |
|  | **2080** | 219 (170) | 438 (492) | 0 (0) | 0 (0) | 238 (197) | 418 (464) | 0 (0) | 0 (0) | 259 (221) | 397 (441) | 0 (0) | 0 (0) | 265 (227) | 390 (435) | 0 (0) | 0 (0) |
| **San Marino** | **2020** | 153 (184) | 196 (252) | 0 (0) | 0 (0) | 150 (181) | 197 (254) | 0 (0) | 0 (0) | 152 (178) | 198 (257) | 0 (0) | 0 (0) | 152 (181) | 198 (254) | 0 (0) | 0 (0) |
|  | **2050** | 152 (141) | 206 (295) | 0 (0) | 0 (0) | 157 (143) | 201 (293) | 0 (0) | 0 (0) | 152 (137) | 207 (299) | 0 (0) | 0 (0) | 157 (144) | 200 (292) | 0 (0) | 0 (0) |
|  | **2080** | 126 (116) | 233 (320) | 0 (0) | 0 (0) | 134 (123) | 224 (313) | 0 (0) | 0 (0) | 141 (123) | 218 (313) | 0 (0) | 0 (0) | 147 (131) | 212 (305) | 0 (0) | 0 (0) |
| **Serbia** | **2020** | 319 (298) | 310 (384) | 1 (0) | 0 (1) | 320 (297) | 312 (385) | 1 (0) | 0 (1) | 315 (301) | 315 (381) | 1 (0) | 0 (1) | 313 (307) | 317 (374) | 1 (0) | 0 (1) |
|  | **2050** | 284 (281) | 357 (401) | 1 (1) | 0 (0) | 295 (292) | 345 (390) | 1 (1) | 0 (0) | 293 (289) | 348 (393) | 1 (1) | 0 (0) | 295 (296) | 345 (386) | 1 (1) | 0 (0) |
|  | **2080** | 215 (211) | 429 (471) | 1 (0) | 0 (1) | 229 (231) | 412 (451) | 1 (1) | 0 (0) | 245 (242) | 399 (440) | 1 (0) | 0 (1) | 250 (252) | 392 (430) | 1 (0) | 0 (1) |
| **Slovakia** | **2020** | 379 (278) | 162 (285) | 0 (0) | 0 (0) | 381 (277) | 161 (286) | 0 (0) | 0 (0) | 379 (276) | 163 (287) | 0 (0) | 0 (0) | 376 (277) | 164 (286) | 0 (0) | 0 (0) |
|  | **2050** | 356 (249) | 194 (314) | 0 (0) | 0 (0) | 361 (252) | 190 (311) | 0 (0) | 0 (0) | 357 (258) | 193 (304) | 0 (0) | 0 (0) | 355 (252) | 194 (311) | 0 (0) | 0 (0) |
|  | **2080** | 263 (161) | 286 (401) | 0 (0) | 0 (0) | 300 (189) | 250 (374) | 0 (0) | 0 (0) | 323 (217) | 223 (346) | 0 (0) | 0 (0) | 322 (215) | 227 (348) | 0 (0) | 0 (0) |
| **Slovenia** | **2020** | 277 (236) | 281 (382) | 1 (1) | 0 (0) | 278 (236) | 279 (381) | 1 (1) | 0 (0) | 274 (234) | 285 (384) | 1 (1) | 0 (0) | 274 (233) | 286 (385) | 1 (1) | 0 (0) |
|  | **2050** | 354 (299) | 220 (319) | 1 (1) | 0 (0) | 357 (305) | 214 (312) | 1 (1) | 0 (0) | 343 (295) | 230 (323) | 1 (1) | 0 (0) | 343 (298) | 232 (320) | 1 (1) | 0 (0) |
|  | **2080** | 284 (227) | 298 (391) | 1 (1) | 0 (0) | 312 (259) | 266 (359) | 1 (1) | 0 (0) | 306 (253) | 268 (365) | 1 (1) | 0 (0) | 306 (252) | 273 (366) | 1 (1) | 0 (0) |
| **Spain** | **2020** | 381 (271) | 441 (561) | 0 (0) | 0 (0) | 387 (272) | 436 (560) | 0 (0) | 0 (0) | 390 (274) | 434 (558) | 0 (0) | 0 (0) | 384 (275) | 439 (557) | 0 (0) | 0 (0) |
|  | **2050** | 318 (223) | 505 (608) | 0 (0) | 0 (0) | 340 (221) | 485 (610) | 0 (0) | 0 (0) | 331 (222) | 494 (610) | 0 (0) | 0 (0) | 335 (228) | 491 (604) | 0 (0) | 0 (0) |
|  | **2080** | 284 (235) | 543 (598) | 0 (0) | 0 (0) | 299 (238) | 527 (594) | 0 (0) | 0 (0) | 315 (250) | 510 (581) | 0 (0) | 0 (0) | 322 (245) | 504 (586) | 0 (0) | 0 (0) |
| **Sweden** | **2020** | 278 (270) | 144 (159) | 0 (0) | 0 (0) | 279 (270) | 144 (159) | 0 (0) | 0 (0) | 281 (269) | 142 (159) | 0 (0) | 0 (0) | 280 (268) | 144 (161) | 0 (0) | 0 (0) |
|  | **2050** | 272 (247) | 152 (181) | 0 (0) | 0 (0) | 270 (247) | 153 (181) | 0 (0) | 0 (0) | 274 (250) | 149 (179) | 0 (0) | 0 (0) | 268 (248) | 155 (181) | 0 (0) | 0 (0) |
|  | **2080** | 232 (210) | 194 (219) | 0 (0) | 0 (0) | 242 (225) | 184 (204) | 0 (0) | 0 (0) | 257 (230) | 168 (199) | 0 (0) | 0 (0) | 247 (229) | 178 (200) | 0 (0) | 0 (0) |
| **Switzerland** | **2020** | 311 (286) | 286 (316) | 0 (0) | 0 (0) | 309 (283) | 286 (319) | 0 (0) | 0 (0) | 310 (287) | 287 (315) | 0 (0) | 0 (0) | 307 (289) | 289 (314) | 0 (0) | 0 (0) |
|  | **2050** | 364 (311) | 239 (291) | 0 (0) | 0 (0) | 373 (322) | 230 (282) | 0 (0) | 0 (0) | 367 (321) | 235 (282) | 0 (0) | 0 (0) | 369 (315) | 234 (289) | 0 (0) | 0 (0) |
|  | **2080** | 305 (264) | 300 (341) | 0 (0) | 0 (0) | 313 (278) | 290 (329) | 0 (0) | 0 (0) | 313 (274) | 290 (329) | 0 (0) | 0 (0) | 310 (269) | 293 (337) | 0 (0) | 0 (0) |
| **UK** | **2020** | 176 (168) | 236 (246) | 0 (0) | 0 (0) | 175 (167) | 237 (247) | 0 (0) | 0 (0) | 175 (168) | 238 (246) | 0 (0) | 0 (0) | 169 (168) | 242 (246) | 0 (0) | 0 (0) |
|  | **2050** | 157 (148) | 256 (267) | 0 (0) | 0 (0) | 158 (150) | 255 (265) | 0 (0) | 0 (0) | 157 (149) | 256 (266) | 0 (0) | 0 (0) | 157 (148) | 256 (267) | 0 (0) | 0 (0) |
|  | **2080** | 143 (137) | 270 (278) | 0 (0) | 0 (0) | 153 (146) | 261 (269) | 0 (0) | 0 (0) | 155 (146) | 258 (269) | 0 (0) | 0 (0) | 155 (143) | 259 (272) | 0 (0) | 0 (0) |

**Table S3b -** Numbers of species projected to gain (win) and lose (los) climatic suitability in Natura 2000 areas (in parenthesis is expected value outside the Natura 2000), for different combinations of countries, emission scenarios and time periods. Projections are provided for Habitat Directive species (HD species), and for the complete pool of vertebrate and plant species considered (all).

|  |  | **A1FI** | | | | **A2** | | | | **B1** | | | | **B2** | | | |
| --- | --- | --- | --- | --- | --- | --- | --- | --- | --- | --- | --- | --- | --- | --- | --- | --- | --- |
|  |  | **all species** | | **HD species** | | **all species** | | **HD species** | | **all species** | | **HD species** | | **all species** | | **HD species** | |
|  |  | **win** | **los** | **win** | **los** | **win** | **los** | **win** | **los** | **win** | **los** | **win** | **los** | **win** | **los** | **win** | **los** |

| ***Amphibians*** | |  |  |  |  |  |  |  |  |  |  |  |  |  |  |  |  |
| --- | --- | --- | --- | --- | --- | --- | --- | --- | --- | --- | --- | --- | --- | --- | --- | --- | --- |
| **Austria** | **2020** | 12 (12) | 8 (8) | 9 (9) | 5 (5) | 12 (12) | 8 (8) | 9 (9) | 5 (5) | 12 (11) | 8 (9) | 9 (8) | 5 (6) | 12 (12) | 8 (8) | 9 (9) | 5 (5) |
|  | **2050** | 14 (13) | 6 (7) | 10 (9) | 4 (5) | 14 (14) | 5 (6) | 10 (9) | 4 (5) | 13 (13) | 6 (6) | 9 (9) | 5 (5) | 13 (15) | 6 (5) | 9 (10) | 5 (4) |
|  | **2080** | 14 (12) | 6 (8) | 9 (8) | 5 (6) | 14 (12) | 6 (8) | 9 (8) | 5 (6) | 14 (15) | 4 (5) | 9 (10) | 4 (4) | 13 (14) | 5 (5) | 9 (10) | 4 (4) |
| **Belgium** | **2020** | 13 (13) | 6 (6) | 8 (8) | 4 (4) | 13 (13) | 6 (6) | 8 (8) | 4 (4) | 13 (13) | 6 (6) | 8 (8) | 4 (4) | 12 (12) | 7 (7) | 7 (7) | 5 (5) |
|  | **2050** | 7 (5) | 12 (14) | 4 (3) | 8 (9) | 6 (5) | 13 (14) | 3 (3) | 9 (9) | 7 (5) | 12 (14) | 4 (3) | 8 (9) | 5 (5) | 14 (14) | 3 (3) | 9 (9) |
|  | **2080** | 5 (3) | 14 (16) | 3 (1) | 9 (11) | 7 (4) | 12 (15) | 4 (2) | 8 (10) | 8 (6) | 11 (13) | 4 (4) | 8 (8) | 7 (6) | 12 (13) | 4 (4) | 8 (8) |
| **Bulgaria** | **2020** | 8 (6) | 9 (13) | 5 (6) | 6 (8) | 8 (7) | 9 (12) | 5 (6) | 6 (8) | 8 (6) | 9 (13) | 5 (6) | 6 (8) | 8 (6) | 9 (13) | 5 (6) | 6 (8) |
|  | **2050** | 12 (9) | 5 (10) | 9 (8) | 2 (6) | 12 (9) | 5 (10) | 9 (8) | 2 (6) | 12 (8) | 5 (11) | 9 (7) | 2 (7) | 12 (9) | 5 (10) | 9 (8) | 2 (6) |
|  | **2080** | 8 (7) | 9 (12) | 6 (6) | 5 (8) | 8 (8) | 9 (11) | 6 (7) | 5 (7) | 10 (8) | 7 (11) | 6 (7) | 5 (7) | 11 (8) | 6 (11) | 7 (7) | 4 (7) |
| **Czech Republic** | **2020** | 16 (7) | 3 (10) | 10 (4) | 3 (7) | 17 (7) | 2 (10) | 11 (4) | 2 (7) | 17 (7) | 2 (10) | 11 (4) | 2 (7) | 17 (8) | 2 (9) | 11 (5) | 2 (6) |
|  | **2050** | 14 (10) | 5 (7) | 10 (7) | 3 (4) | 14 (11) | 5 (6) | 10 (8) | 3 (3) | 15 (10) | 4 (7) | 10 (7) | 3 (4) | 14 (11) | 5 (6) | 10 (8) | 3 (3) |
|  | **2080** | 13 (9) | 6 (8) | 8 (5) | 5 (6) | 14 (9) | 5 (8) | 9 (6) | 4 (5) | 14 (9) | 5 (8) | 9 (5) | 4 (6) | 14 (10) | 5 (7) | 9 (6) | 4 (5) |
| **Denmark** | **2020** | 9 (11) | 5 (8) | 7 (8) | 3 (5) | 9 (12) | 5 (7) | 7 (8) | 3 (5) | 9 (13) | 5 (6) | 7 (8) | 3 (5) | 9 (13) | 5 (6) | 7 (8) | 3 (5) |
|  | **2050** | 9 (11) | 5 (8) | 6 (9) | 4 (4) | 9 (13) | 5 (6) | 6 (9) | 4 (4) | 9 (13) | 5 (6) | 6 (9) | 4 (4) | 9 (13) | 5 (6) | 6 (9) | 4 (4) |
|  | **2080** | 7 (7) | 8 (12) | 4 (5) | 6 (8) | 7 (10) | 7 (9) | 5 (7) | 5 (6) | 7 (12) | 7 (7) | 5 (8) | 5 (5) | 7 (12) | 7 (7) | 5 (8) | 5 (5) |
| **Estonia** | **2020** | 10 (10) | 1 (4) | 8 (7) | 0 (3) | 10 (10) | 1 (4) | 8 (7) | 0 (3) | 11 (10) | 0 (4) | 8 (7) | 0 (3) | 11 (10) | 0 (4) | 8 (7) | 0 (3) |
|  | **2050** | 11 (10) | 0 (4) | 8 (7) | 0 (3) | 11 (10) | 0 (4) | 8 (7) | 0 (3) | 11 (10) | 0 (4) | 8 (7) | 0 (3) | 11 (9) | 0 (5) | 8 (6) | 0 (4) |
|  | **2080** | 9 (8) | 2 (7) | 6 (5) | 2 (5) | 11 (7) | 0 (7) | 8 (5) | 0 (5) | 11 (8) | 0 (6) | 8 (6) | 0 (4) | 11 (7) | 0 (7) | 8 (5) | 0 (5) |
| **Finland** | **2020** | 4 (11) | 1 (0) | 2 (8) | 1 (0) | 4 (11) | 1 (0) | 2 (8) | 1 (0) | 5 (11) | 0 (0) | 3 (8) | 0 (0) | 5 (11) | 0 (0) | 3 (8) | 0 (0) |
|  | **2050** | 5 (11) | 0 (0) | 3 (8) | 0 (0) | 5 (11) | 0 (0) | 3 (8) | 0 (0) | 5 (11) | 0 (0) | 3 (8) | 0 (0) | 5 (11) | 0 (0) | 3 (8) | 0 (0) |
|  | **2080** | 5 (11) | 0 (0) | 3 (8) | 0 (0) | 5 (11) | 0 (0) | 3 (8) | 0 (0) | 5 (11) | 0 (0) | 3 (8) | 0 (0) | 5 (11) | 0 (0) | 3 (8) | 0 (0) |
| **France** | **2020** | 10 (4) | 17 (1) | 8 (2) | 11 (1) | 11 (4) | 16 (1) | 9 (2) | 10 (1) | 10 (4) | 17 (1) | 8 (2) | 11 (1) | 10 (4) | 17 (1) | 8 (2) | 11 (1) |
|  | **2050** | 8 (4) | 19 (1) | 6 (2) | 13 (1) | 9 (4) | 18 (1) | 7 (2) | 12 (1) | 9 (4) | 18 (1) | 7 (2) | 12 (1) | 9 (4) | 18 (1) | 7 (2) | 12 (1) |
|  | **2080** | 8 (4) | 20 (1) | 6 (2) | 14 (1) | 8 (4) | 20 (1) | 6 (2) | 14 (1) | 9 (4) | 19 (1) | 7 (2) | 13 (1) | 9 (4) | 19 (1) | 7 (2) | 13 (1) |
| **Germany** | **2020** | 11 (9) | 9 (18) | 7 (8) | 7 (11) | 11 (10) | 9 (17) | 7 (9) | 7 (10) | 13 (9) | 7 (18) | 9 (8) | 5 (11) | 13 (10) | 7 (17) | 9 (9) | 5 (10) |
|  | **2050** | 10 (6) | 10 (21) | 8 (4) | 6 (15) | 12 (6) | 8 (21) | 8 (4) | 6 (15) | 12 (4) | 8 (23) | 8 (3) | 6 (16) | 12 (6) | 8 (21) | 8 (4) | 6 (15) |
|  | **2080** | 4 (8) | 16 (19) | 3 (6) | 11 (13) | 5 (7) | 15 (20) | 3 (5) | 11 (14) | 7 (6) | 13 (21) | 5 (5) | 9 (14) | 5 (7) | 15 (20) | 3 (5) | 11 (14) |
| **Greece** | **2020** | 2 (10) | 13 (10) | 1 (6) | 8 (8) | 2 (11) | 13 (9) | 1 (7) | 8 (7) | 2 (11) | 13 (9) | 1 (7) | 8 (7) | 2 (11) | 13 (9) | 1 (7) | 8 (7) |
|  | **2050** | 10 (9) | 5 (11) | 7 (7) | 2 (7) | 10 (10) | 5 (10) | 7 (8) | 2 (6) | 10 (11) | 5 (9) | 7 (8) | 2 (6) | 10 (10) | 5 (10) | 7 (8) | 2 (6) |
|  | **2080** | 2 (3) | 13 (17) | 1 (2) | 8 (12) | 3 (5) | 12 (15) | 1 (3) | 8 (11) | 2 (6) | 13 (14) | 0 (4) | 9 (10) | 4 (6) | 11 (14) | 2 (4) | 7 (10) |
| **Hungary** | **2020** | 1 (2) | 15 (13) | 1 (1) | 10 (8) | 1 (2) | 15 (13) | 1 (1) | 10 (8) | 1 (2) | 15 (13) | 1 (1) | 10 (8) | 1 (2) | 15 (13) | 1 (1) | 10 (8) |
|  | **2050** | 5 (7) | 11 (8) | 3 (5) | 8 (4) | 5 (8) | 11 (7) | 3 (5) | 8 (4) | 6 (7) | 10 (8) | 4 (5) | 7 (4) | 6 (9) | 10 (6) | 4 (6) | 7 (3) |
|  | **2080** | 7 (2) | 9 (13) | 4 (1) | 7 (8) | 7 (2) | 9 (13) | 4 (1) | 7 (8) | 7 (1) | 9 (14) | 4 (0) | 7 (9) | 7 (1) | 8 (14) | 4 (0) | 6 (9) |
| **Ireland** | **2020** | 2 (2) | 1 (14) | 2 (1) | 0 (10) | 2 (2) | 1 (14) | 2 (1) | 0 (10) | 2 (2) | 1 (14) | 2 (2) | 0 (9) | 2 (2) | 1 (14) | 2 (2) | 0 (9) |
|  | **2050** | 1 (4) | 2 (12) | 1 (2) | 1 (9) | 1 (4) | 2 (12) | 1 (2) | 1 (9) | 1 (6) | 2 (10) | 1 (4) | 1 (7) | 1 (6) | 2 (10) | 1 (4) | 1 (7) |
|  | **2080** | 1 (7) | 2 (9) | 1 (4) | 1 (7) | 1 (8) | 2 (8) | 1 (5) | 1 (6) | 1 (8) | 2 (8) | 1 (5) | 1 (6) | 1 (8) | 2 (8) | 1 (5) | 1 (6) |
| **Italy** | **2020** | 20 (2) | 7 (1) | 16 (2) | 4 (0) | 20 (2) | 7 (1) | 16 (2) | 4 (0) | 20 (2) | 7 (1) | 16 (2) | 4 (0) | 20 (2) | 7 (1) | 16 (2) | 4 (0) |
|  | **2050** | 20 (1) | 8 (2) | 16 (1) | 5 (1) | 21 (1) | 7 (2) | 16 (1) | 5 (1) | 21 (1) | 7 (2) | 17 (1) | 4 (1) | 23 (1) | 5 (2) | 18 (1) | 3 (1) |
|  | **2080** | 18 (1) | 10 (2) | 15 (1) | 6 (1) | 19 (1) | 9 (2) | 16 (1) | 5 (1) | 18 (1) | 10 (2) | 15 (1) | 6 (1) | 19 (1) | 9 (2) | 16 (1) | 5 (1) |
| **Latvia** | **2020** | 12 (16) | 0 (12) | 9 (13) | 0 (8) | 12 (15) | 0 (13) | 9 (13) | 0 (8) | 12 (15) | 0 (12) | 9 (13) | 0 (7) | 12 (15) | 0 (13) | 9 (13) | 0 (8) |
|  | **2050** | 12 (17) | 0 (11) | 9 (14) | 0 (7) | 12 (17) | 0 (11) | 9 (14) | 0 (7) | 12 (17) | 0 (11) | 9 (14) | 0 (7) | 12 (17) | 0 (11) | 9 (14) | 0 (7) |
|  | **2080** | 10 (14) | 2 (14) | 7 (12) | 2 (9) | 11 (14) | 1 (14) | 8 (12) | 1 (9) | 11 (15) | 1 (13) | 8 (12) | 1 (9) | 11 (15) | 1 (13) | 8 (13) | 1 (8) |
| **Lithuania** | **2020** | 12 (12) | 1 (0) | 9 (9) | 1 (0) | 12 (12) | 1 (0) | 9 (9) | 1 (0) | 12 (12) | 1 (0) | 9 (9) | 1 (0) | 12 (12) | 1 (0) | 9 (9) | 1 (0) |
|  | **2050** | 12 (12) | 1 (0) | 9 (9) | 1 (0) | 12 (12) | 1 (0) | 9 (9) | 1 (0) | 12 (12) | 1 (0) | 9 (9) | 1 (0) | 12 (12) | 1 (0) | 9 (9) | 1 (0) |
|  | **2080** | 7 (9) | 6 (3) | 5 (6) | 5 (3) | 10 (11) | 3 (1) | 7 (8) | 3 (1) | 12 (11) | 1 (1) | 9 (8) | 1 (1) | 11 (11) | 2 (1) | 8 (8) | 2 (1) |
| **Luxembourg** | **2020** | 15 (12) | 4 (1) | 9 (9) | 3 (1) | 15 (12) | 4 (1) | 9 (9) | 3 (1) | 15 (12) | 4 (1) | 9 (9) | 3 (1) | 15 (12) | 4 (1) | 9 (9) | 3 (1) |
|  | **2050** | 11 (12) | 8 (1) | 7 (9) | 5 (1) | 11 (12) | 8 (1) | 7 (9) | 5 (1) | 11 (12) | 8 (1) | 7 (9) | 5 (1) | 9 (12) | 10 (1) | 5 (9) | 7 (1) |
|  | **2080** | 3 (8) | 16 (5) | 2 (5) | 10 (5) | 8 (10) | 11 (3) | 4 (7) | 8 (3) | 8 (12) | 11 (1) | 4 (9) | 8 (1) | 8 (11) | 11 (2) | 4 (8) | 8 (2) |
| **Malta** | **2020** | 2 (15) | 0 (4) | 1 (9) | 0 (3) | 2 (15) | 0 (4) | 1 (9) | 0 (3) | 2 (15) | 0 (4) | 1 (9) | 0 (3) | 2 (15) | 0 (4) | 1 (9) | 0 (3) |
|  | **2050** | 2 (11) | 0 (8) | 1 (7) | 0 (5) | 2 (11) | 0 (8) | 1 (7) | 0 (5) | 2 (11) | 0 (8) | 1 (7) | 0 (5) | 2 (9) | 0 (10) | 1 (5) | 0 (7) |
|  | **2080** | 2 (3) | 0 (16) | 1 (2) | 0 (10) | 2 (7) | 0 (12) | 1 (3) | 0 (9) | 2 (8) | 0 (11) | 1 (4) | 0 (8) | 2 (8) | 0 (11) | 1 (4) | 0 (8) |
| **Netherlands** | **2020** | 10 (2) | 8 (0) | 6 (1) | 6 (0) | 9 (2) | 9 (0) | 6 (1) | 6 (0) | 9 (2) | 9 (0) | 6 (1) | 6 (0) | 8 (2) | 10 (0) | 5 (1) | 7 (0) |
|  | **2050** | 8 (2) | 10 (0) | 5 (1) | 7 (0) | 8 (2) | 10 (0) | 5 (1) | 7 (0) | 8 (2) | 10 (0) | 5 (1) | 7 (0) | 8 (2) | 10 (0) | 5 (1) | 7 (0) |
|  | **2080** | 5 (2) | 13 (0) | 3 (1) | 9 (0) | 5 (2) | 13 (0) | 3 (1) | 9 (0) | 7 (2) | 11 (0) | 4 (1) | 8 (0) | 5 (2) | 13 (0) | 3 (1) | 9 (0) |
| **Poland** | **2020** | 16 (9) | 2 (9) | 11 (6) | 2 (6) | 16 (8) | 2 (10) | 11 (5) | 2 (7) | 16 (9) | 2 (9) | 11 (6) | 2 (6) | 16 (7) | 2 (11) | 11 (4) | 2 (8) |
|  | **2050** | 11 (7) | 7 (11) | 7 (4) | 6 (8) | 12 (8) | 6 (10) | 8 (5) | 5 (7) | 12 (9) | 6 (9) | 8 (6) | 5 (6) | 12 (8) | 6 (10) | 8 (5) | 5 (7) |
|  | **2080** | 8 (5) | 10 (13) | 5 (3) | 8 (9) | 8 (6) | 10 (12) | 5 (3) | 8 (9) | 10 (6) | 8 (12) | 6 (4) | 7 (8) | 10 (6) | 8 (12) | 6 (4) | 7 (8) |
| **Portugal** | **2020** | 5 (15) | 14 (3) | 4 (10) | 8 (3) | 6 (15) | 13 (3) | 4 (10) | 8 (3) | 7 (15) | 12 (3) | 5 (10) | 7 (3) | 7 (15) | 12 (3) | 5 (10) | 7 (3) |
|  | **2050** | 1 (9) | 18 (9) | 0 (6) | 12 (7) | 1 (10) | 18 (8) | 0 (7) | 12 (6) | 1 (11) | 18 (7) | 0 (7) | 12 (6) | 3 (11) | 16 (7) | 1 (7) | 11 (6) |
|  | **2080** | 1 (7) | 18 (11) | 1 (5) | 11 (8) | 1 (8) | 18 (10) | 1 (5) | 11 (8) | 2 (9) | 17 (9) | 1 (6) | 11 (7) | 2 (9) | 17 (9) | 1 (6) | 11 (7) |
| **Romania** | **2020** | 11 (5) | 7 (14) | 6 (3) | 7 (9) | 11 (4) | 7 (15) | 6 (2) | 7 (10) | 11 (6) | 7 (13) | 6 (4) | 7 (8) | 11 (6) | 7 (13) | 6 (4) | 7 (8) |
|  | **2050** | 13 (2) | 5 (17) | 11 (1) | 2 (11) | 13 (2) | 5 (17) | 11 (1) | 2 (11) | 14 (2) | 4 (17) | 11 (1) | 2 (11) | 14 (3) | 4 (16) | 11 (1) | 2 (11) |
|  | **2080** | 10 (2) | 8 (17) | 7 (1) | 6 (11) | 11 (2) | 7 (17) | 7 (1) | 6 (11) | 12 (3) | 6 (16) | 7 (1) | 6 (11) | 11 (3) | 7 (16) | 7 (1) | 6 (11) |
| **Slovakia** | **2020** | 12 (9) | 6 (9) | 8 (6) | 5 (7) | 12 (9) | 6 (9) | 8 (6) | 5 (7) | 12 (9) | 6 (9) | 8 (6) | 5 (7) | 12 (9) | 6 (9) | 8 (6) | 5 (7) |
|  | **2050** | 11 (7) | 7 (11) | 8 (5) | 5 (8) | 12 (8) | 6 (10) | 8 (6) | 5 (7) | 12 (9) | 6 (9) | 8 (7) | 5 (6) | 12 (9) | 6 (9) | 8 (7) | 5 (6) |
|  | **2080** | 8 (7) | 10 (11) | 6 (5) | 7 (8) | 9 (8) | 9 (10) | 6 (6) | 7 (7) | 11 (8) | 7 (10) | 7 (6) | 6 (7) | 10 (8) | 8 (10) | 6 (6) | 7 (7) |
| **Slovenia** | **2020** | 5 (7) | 14 (11) | 5 (6) | 9 (7) | 5 (7) | 14 (11) | 5 (6) | 9 (7) | 5 (7) | 14 (11) | 5 (5) | 9 (8) | 5 (7) | 14 (11) | 5 (5) | 9 (8) |
|  | **2050** | 8 (7) | 11 (11) | 7 (5) | 7 (8) | 9 (8) | 10 (10) | 8 (6) | 6 (7) | 8 (7) | 11 (11) | 7 (5) | 7 (8) | 9 (7) | 10 (11) | 8 (5) | 6 (8) |
|  | **2080** | 6 (6) | 13 (12) | 5 (5) | 9 (8) | 6 (6) | 13 (12) | 5 (5) | 9 (8) | 5 (8) | 14 (10) | 5 (6) | 9 (7) | 5 (9) | 14 (9) | 5 (6) | 9 (7) |
| **Spain** | **2020** | 17 (5) | 8 (14) | 11 (5) | 6 (9) | 17 (5) | 8 (14) | 11 (5) | 6 (9) | 19 (4) | 6 (15) | 13 (4) | 4 (10) | 20 (4) | 5 (15) | 14 (4) | 3 (10) |
|  | **2050** | 6 (7) | 19 (12) | 4 (6) | 13 (8) | 8 (7) | 17 (12) | 6 (6) | 11 (8) | 7 (7) | 18 (12) | 5 (6) | 12 (8) | 9 (7) | 16 (12) | 7 (6) | 10 (8) |
|  | **2080** | 8 (5) | 17 (14) | 6 (4) | 11 (10) | 8 (5) | 17 (14) | 6 (4) | 11 (10) | 9 (5) | 16 (14) | 6 (5) | 11 (9) | 9 (5) | 16 (14) | 6 (5) | 11 (9) |
| **Sweden** | **2020** | 10 (6) | 2 (19) | 7 (5) | 2 (12) | 10 (6) | 2 (19) | 7 (5) | 2 (12) | 10 (6) | 2 (19) | 7 (5) | 2 (12) | 10 (8) | 2 (17) | 7 (6) | 2 (11) |
|  | **2050** | 10 (3) | 2 (22) | 7 (2) | 2 (15) | 10 (3) | 2 (22) | 7 (2) | 2 (15) | 10 (3) | 2 (22) | 7 (2) | 2 (15) | 10 (4) | 2 (21) | 7 (3) | 2 (14) |
|  | **2080** | 8 (5) | 4 (20) | 6 (3) | 3 (14) | 8 (6) | 4 (19) | 6 (4) | 3 (13) | 10 (7) | 2 (18) | 7 (5) | 2 (12) | 10 (7) | 2 (18) | 7 (5) | 2 (12) |
| **UK** | **2020** | 5 (10) | 3 (2) | 3 (7) | 2 (2) | 5 (10) | 3 (2) | 3 (7) | 2 (2) | 5 (11) | 3 (1) | 3 (8) | 2 (1) | 5 (11) | 3 (1) | 3 (8) | 2 (1) |
|  | **2050** | 4 (11) | 4 (1) | 2 (8) | 3 (1) | 4 (11) | 4 (1) | 2 (8) | 3 (1) | 4 (11) | 4 (1) | 2 (8) | 3 (1) | 5 (11) | 3 (1) | 3 (8) | 2 (1) |
|  | **2080** | 3 (7) | 5 (5) | 1 (5) | 4 (4) | 4 (8) | 4 (4) | 2 (6) | 3 (3) | 4 (10) | 4 (2) | 2 (7) | 3 (2) | 4 (10) | 4 (2) | 2 (7) | 3 (2) |

| ***Reptiles*** | |  |  |  |  |  |  |  |  |  |  |  |  |  |  |  |  |
| --- | --- | --- | --- | --- | --- | --- | --- | --- | --- | --- | --- | --- | --- | --- | --- | --- | --- |
| **Austria** | **2020** | 10 (10) | 3 (3) | 6 (6) | 1 (1) | 10 (10) | 3 (3) | 6 (6) | 1 (1) | 10 (10) | 3 (3) | 6 (6) | 1 (1) | 10 (10) | 3 (3) | 6 (6) | 1 (1) |
|  | **2050** | 9 (10) | 4 (3) | 6 (7) | 1 (0) | 10 (10) | 3 (3) | 7 (7) | 0 (0) | 10 (10) | 3 (3) | 7 (7) | 0 (0) | 10 (10) | 3 (3) | 7 (7) | 0 (0) |
|  | **2080** | 11 (10) | 2 (3) | 6 (6) | 1 (1) | 10 (10) | 3 (3) | 6 (6) | 1 (1) | 10 (10) | 3 (3) | 6 (6) | 1 (1) | 10 (10) | 3 (3) | 6 (6) | 1 (1) |
| **Belgium** | **2020** | 3 (2) | 4 (5) | 1 (0) | 2 (3) | 3 (2) | 4 (5) | 1 (0) | 2 (3) | 3 (2) | 4 (5) | 1 (0) | 2 (3) | 2 (2) | 5 (5) | 0 (0) | 3 (3) |
|  | **2050** | 2 (2) | 5 (5) | 1 (1) | 2 (2) | 2 (2) | 5 (5) | 1 (1) | 2 (2) | 2 (2) | 5 (5) | 1 (1) | 2 (2) | 3 (3) | 4 (4) | 2 (2) | 1 (1) |
|  | **2080** | 3 (3) | 4 (4) | 1 (1) | 2 (2) | 3 (2) | 4 (5) | 1 (1) | 2 (2) | 2 (2) | 5 (5) | 1 (1) | 2 (2) | 3 (2) | 4 (5) | 1 (1) | 2 (2) |
| **Bulgaria** | **2020** | 24 (23) | 5 (2) | 14 (14) | 2 (1) | 24 (23) | 5 (2) | 14 (14) | 2 (1) | 22 (23) | 7 (2) | 11 (14) | 5 (1) | 25 (23) | 4 (2) | 14 (14) | 2 (1) |
|  | **2050** | 20 (21) | 9 (5) | 15 (13) | 1 (2) | 21 (20) | 8 (6) | 15 (13) | 1 (2) | 20 (21) | 9 (5) | 15 (13) | 1 (2) | 21 (21) | 8 (5) | 15 (13) | 1 (2) |
|  | **2080** | 24 (22) | 5 (4) | 14 (13) | 2 (2) | 24 (22) | 5 (4) | 14 (13) | 2 (2) | 24 (22) | 5 (4) | 14 (13) | 2 (2) | 24 (22) | 5 (4) | 14 (13) | 2 (2) |
| **Czech Republic** | **2020** | 10 (25) | 0 (4) | 6 (14) | 0 (2) | 10 (25) | 0 (4) | 6 (14) | 0 (2) | 10 (26) | 0 (3) | 6 (14) | 0 (2) | 10 (25) | 0 (4) | 6 (14) | 0 (2) |
|  | **2050** | 7 (20) | 3 (9) | 6 (15) | 0 (1) | 7 (20) | 3 (9) | 6 (15) | 0 (1) | 7 (20) | 3 (9) | 6 (15) | 0 (1) | 7 (21) | 3 (8) | 6 (15) | 0 (1) |
|  | **2080** | 7 (24) | 3 (5) | 5 (14) | 1 (2) | 7 (24) | 3 (5) | 5 (14) | 1 (2) | 8 (24) | 2 (5) | 6 (14) | 0 (2) | 8 (24) | 2 (5) | 6 (14) | 0 (2) |
| **Denmark** | **2020** | 5 (10) | 2 (0) | 1 (6) | 2 (0) | 5 (10) | 2 (0) | 1 (6) | 2 (0) | 5 (10) | 2 (0) | 1 (6) | 2 (0) | 5 (10) | 2 (0) | 1 (6) | 2 (0) |
|  | **2050** | 6 (6) | 1 (4) | 3 (5) | 0 (1) | 6 (7) | 1 (3) | 3 (6) | 0 (0) | 6 (7) | 1 (3) | 3 (6) | 0 (0) | 6 (7) | 1 (3) | 3 (6) | 0 (0) |
|  | **2080** | 4 (7) | 3 (3) | 2 (5) | 1 (1) | 5 (7) | 2 (3) | 2 (5) | 1 (1) | 5 (8) | 2 (2) | 2 (6) | 1 (0) | 5 (6) | 2 (4) | 2 (5) | 1 (1) |
| **Estonia** | **2020** | 5 (6) | 0 (1) | 2 (2) | 0 (1) | 5 (6) | 0 (1) | 2 (2) | 0 (1) | 5 (6) | 0 (1) | 2 (2) | 0 (1) | 5 (6) | 0 (1) | 2 (2) | 0 (1) |
|  | **2050** | 4 (5) | 0 (2) | 2 (2) | 0 (1) | 3 (6) | 1 (1) | 2 (2) | 0 (1) | 3 (6) | 1 (1) | 2 (2) | 0 (1) | 3 (5) | 1 (2) | 2 (2) | 0 (1) |
|  | **2080** | 4 (5) | 0 (2) | 2 (2) | 0 (1) | 4 (5) | 0 (2) | 2 (2) | 0 (1) | 4 (5) | 0 (2) | 2 (2) | 0 (1) | 4 (5) | 0 (2) | 2 (2) | 0 (1) |
| **Finland** | **2020** | 2 (4) | 2 (1) | 1 (2) | 0 (0) | 2 (5) | 2 (0) | 1 (2) | 0 (0) | 2 (4) | 2 (1) | 1 (2) | 0 (0) | 2 (5) | 2 (0) | 1 (2) | 0 (0) |
|  | **2050** | 3 (3) | 1 (1) | 1 (2) | 0 (0) | 3 (3) | 1 (1) | 1 (2) | 0 (0) | 3 (3) | 1 (1) | 1 (2) | 0 (0) | 3 (3) | 1 (1) | 1 (2) | 0 (0) |
|  | **2080** | 5 (4) | 0 (0) | 1 (2) | 0 (0) | 4 (4) | 1 (0) | 1 (2) | 0 (0) | 3 (4) | 1 (0) | 1 (2) | 0 (0) | 3 (4) | 1 (0) | 1 (2) | 0 (0) |
| **France** | **2020** | 21 (4) | 11 (1) | 10 (1) | 7 (0) | 21 (4) | 11 (1) | 10 (1) | 7 (0) | 21 (4) | 11 (1) | 10 (1) | 7 (0) | 21 (4) | 11 (1) | 10 (1) | 7 (0) |
|  | **2050** | 21 (4) | 13 (1) | 12 (1) | 6 (0) | 20 (4) | 13 (1) | 12 (1) | 6 (0) | 19 (3) | 14 (2) | 12 (1) | 6 (0) | 19 (3) | 14 (1) | 12 (1) | 6 (0) |
|  | **2080** | 21 (4) | 14 (1) | 11 (1) | 8 (0) | 21 (4) | 13 (1) | 11 (1) | 7 (0) | 21 (4) | 13 (1) | 11 (1) | 7 (0) | 21 (4) | 13 (1) | 11 (1) | 7 (0) |
| **Germany** | **2020** | 6 (20) | 6 (13) | 2 (11) | 4 (7) | 5 (20) | 6 (14) | 2 (11) | 4 (8) | 5 (20) | 6 (13) | 2 (11) | 4 (7) | 5 (20) | 6 (13) | 2 (11) | 4 (7) |
|  | **2050** | 8 (22) | 4 (12) | 5 (13) | 1 (5) | 8 (20) | 4 (13) | 5 (13) | 1 (5) | 8 (20) | 4 (13) | 5 (13) | 1 (5) | 7 (19) | 5 (14) | 5 (12) | 1 (6) |
|  | **2080** | 8 (20) | 4 (15) | 5 (10) | 1 (9) | 8 (21) | 4 (14) | 5 (11) | 1 (8) | 9 (21) | 3 (14) | 5 (11) | 1 (8) | 8 (21) | 4 (14) | 5 (11) | 1 (8) |
| **Greece** | **2020** | 12 (6) | 25 (6) | 6 (2) | 15 (4) | 14 (5) | 23 (7) | 8 (2) | 13 (4) | 15 (6) | 22 (6) | 7 (3) | 14 (3) | 16 (6) | 21 (6) | 10 (3) | 11 (3) |
|  | **2050** | 23 (8) | 14 (4) | 16 (5) | 5 (1) | 23 (8) | 14 (4) | 16 (5) | 5 (1) | 23 (8) | 14 (4) | 16 (5) | 5 (1) | 23 (7) | 14 (5) | 16 (5) | 5 (1) |
|  | **2080** | 27 (8) | 10 (4) | 16 (5) | 5 (1) | 27 (8) | 10 (4) | 17 (5) | 4 (1) | 27 (9) | 10 (3) | 16 (5) | 5 (1) | 26 (8) | 11 (4) | 17 (5) | 4 (1) |
| **Hungary** | **2020** | 11 (12) | 3 (25) | 7 (7) | 1 (14) | 11 (12) | 3 (25) | 7 (7) | 1 (14) | 11 (12) | 3 (25) | 7 (7) | 1 (14) | 10 (16) | 4 (21) | 7 (11) | 1 (10) |
|  | **2050** | 9 (22) | 5 (15) | 7 (16) | 1 (5) | 9 (22) | 5 (15) | 7 (16) | 1 (5) | 10 (22) | 4 (15) | 7 (16) | 1 (5) | 10 (22) | 4 (15) | 7 (16) | 1 (5) |
|  | **2080** | 11 (22) | 3 (15) | 7 (13) | 1 (8) | 12 (23) | 2 (14) | 7 (14) | 1 (7) | 12 (23) | 2 (14) | 7 (14) | 1 (7) | 12 (25) | 2 (12) | 7 (16) | 1 (5) |
| **Ireland** | **2020** | 2 (11) | 0 (3) | 1 (7) | 0 (1) | 2 (11) | 0 (3) | 1 (7) | 0 (1) | 2 (11) | 0 (3) | 1 (7) | 0 (1) | 2 (11) | 0 (3) | 1 (7) | 0 (1) |
|  | **2050** | 1 (10) | 1 (4) | 1 (7) | 0 (1) | 1 (10) | 1 (4) | 1 (7) | 0 (1) | 1 (9) | 1 (5) | 1 (7) | 0 (1) | 1 (9) | 1 (5) | 1 (7) | 0 (1) |
|  | **2080** | 1 (11) | 1 (3) | 1 (7) | 0 (1) | 1 (12) | 1 (2) | 1 (7) | 0 (1) | 1 (12) | 1 (2) | 1 (7) | 0 (1) | 1 (12) | 1 (2) | 1 (7) | 0 (1) |
| **Italy** | **2020** | 26 (2) | 14 (0) | 14 (1) | 9 (0) | 26 (2) | 14 (0) | 14 (1) | 9 (0) | 28 (2) | 12 (0) | 14 (1) | 9 (0) | 27 (2) | 13 (0) | 14 (1) | 9 (0) |
|  | **2050** | 29 (1) | 12 (1) | 19 (1) | 5 (0) | 29 (1) | 12 (1) | 18 (1) | 6 (0) | 30 (1) | 11 (1) | 19 (1) | 5 (0) | 30 (1) | 11 (1) | 19 (1) | 5 (0) |
|  | **2080** | 28 (1) | 13 (1) | 18 (1) | 6 (0) | 28 (1) | 13 (1) | 18 (1) | 6 (0) | 28 (1) | 13 (1) | 18 (1) | 6 (0) | 28 (1) | 13 (1) | 18 (1) | 6 (0) |
| **Latvia** | **2020** | 5 (21) | 0 (19) | 3 (10) | 0 (13) | 5 (22) | 0 (18) | 3 (11) | 0 (12) | 5 (22) | 0 (19) | 3 (11) | 0 (13) | 5 (23) | 0 (17) | 3 (12) | 0 (11) |
|  | **2050** | 5 (27) | 0 (14) | 3 (19) | 0 (5) | 5 (26) | 0 (15) | 3 (18) | 0 (6) | 5 (26) | 0 (15) | 3 (18) | 0 (6) | 5 (26) | 0 (15) | 3 (18) | 0 (6) |
|  | **2080** | 4 (26) | 1 (15) | 3 (16) | 0 (8) | 5 (26) | 0 (15) | 3 (16) | 0 (8) | 5 (25) | 0 (16) | 3 (15) | 0 (9) | 5 (26) | 0 (15) | 3 (16) | 0 (8) |
| **Lithuania** | **2020** | 5 (6) | 0 (0) | 3 (3) | 0 (0) | 5 (6) | 0 (0) | 3 (3) | 0 (0) | 5 (6) | 0 (0) | 3 (3) | 0 (0) | 5 (6) | 0 (0) | 3 (3) | 0 (0) |
|  | **2050** | 5 (5) | 0 (0) | 3 (3) | 0 (0) | 5 (5) | 0 (0) | 3 (3) | 0 (0) | 5 (5) | 0 (0) | 3 (3) | 0 (0) | 5 (5) | 0 (0) | 3 (3) | 0 (0) |
|  | **2080** | 5 (4) | 0 (1) | 3 (3) | 0 (0) | 5 (6) | 0 (0) | 3 (3) | 0 (0) | 5 (5) | 0 (0) | 3 (3) | 0 (0) | 5 (5) | 0 (0) | 3 (3) | 0 (0) |
| **Luxembourg** | **2020** | 5 (5) | 3 (0) | 2 (3) | 1 (0) | 5 (5) | 3 (0) | 2 (3) | 1 (0) | 4 (5) | 3 (0) | 2 (3) | 1 (0) | 4 (6) | 3 (0) | 2 (3) | 1 (0) |
|  | **2050** | 4 (5) | 3 (0) | 2 (3) | 1 (0) | 5 (5) | 2 (0) | 2 (3) | 1 (0) | 5 (5) | 2 (0) | 2 (3) | 1 (0) | 5 (5) | 2 (0) | 2 (3) | 1 (0) |
|  | **2080** | 5 (4) | 3 (1) | 2 (3) | 1 (0) | 5 (6) | 3 (0) | 2 (3) | 1 (0) | 5 (5) | 3 (0) | 2 (3) | 1 (0) | 5 (6) | 3 (0) | 2 (3) | 1 (0) |
| **Malta** | **2020** | 8 (5) | 0 (3) | 3 (2) | 0 (1) | 8 (5) | 0 (3) | 3 (2) | 0 (1) | 8 (5) | 0 (3) | 3 (2) | 0 (1) | 8 (5) | 0 (3) | 3 (2) | 0 (1) |
|  | **2050** | 8 (5) | 1 (3) | 3 (2) | 0 (1) | 8 (6) | 1 (2) | 3 (2) | 0 (1) | 8 (6) | 1 (2) | 3 (2) | 0 (1) | 7 (6) | 1 (2) | 3 (2) | 0 (1) |
|  | **2080** | 8 (5) | 1 (3) | 2 (2) | 1 (1) | 8 (5) | 1 (3) | 2 (2) | 1 (1) | 9 (5) | 0 (3) | 3 (2) | 0 (1) | 8 (5) | 0 (3) | 2 (2) | 0 (1) |
| **Netherlands** | **2020** | 3 (9) | 4 (0) | 1 (3) | 2 (0) | 2 (9) | 5 (0) | 1 (3) | 2 (0) | 2 (9) | 5 (0) | 1 (3) | 2 (0) | 2 (9) | 5 (0) | 1 (3) | 2 (0) |
|  | **2050** | 3 (8) | 4 (1) | 2 (3) | 1 (0) | 3 (8) | 4 (1) | 2 (3) | 1 (0) | 3 (8) | 4 (1) | 2 (3) | 1 (0) | 3 (8) | 4 (1) | 2 (3) | 1 (0) |
|  | **2080** | 3 (9) | 4 (0) | 2 (3) | 1 (0) | 3 (9) | 4 (0) | 2 (3) | 1 (0) | 3 (9) | 4 (0) | 2 (3) | 1 (0) | 3 (9) | 4 (0) | 2 (3) | 1 (0) |
| **Poland** | **2020** | 8 (3) | 1 (4) | 4 (1) | 1 (2) | 8 (3) | 1 (4) | 4 (1) | 1 (2) | 8 (3) | 1 (4) | 4 (1) | 1 (2) | 8 (3) | 1 (4) | 4 (1) | 1 (2) |
|  | **2050** | 8 (3) | 2 (4) | 6 (2) | 0 (1) | 7 (3) | 2 (4) | 6 (2) | 0 (1) | 7 (3) | 3 (4) | 6 (2) | 0 (1) | 7 (3) | 3 (4) | 6 (2) | 0 (1) |
|  | **2080** | 7 (3) | 3 (4) | 5 (2) | 1 (1) | 7 (3) | 3 (4) | 5 (2) | 1 (1) | 6 (3) | 4 (4) | 5 (2) | 1 (1) | 7 (3) | 3 (4) | 5 (2) | 1 (1) |
| **Portugal** | **2020** | 10 (8) | 18 (1) | 7 (4) | 8 (1) | 9 (8) | 19 (1) | 7 (4) | 8 (1) | 11 (8) | 17 (1) | 8 (4) | 7 (1) | 12 (8) | 16 (1) | 8 (4) | 7 (1) |
|  | **2050** | 10 (6) | 18 (4) | 8 (5) | 7 (1) | 10 (7) | 18 (3) | 8 (6) | 7 (0) | 12 (6) | 16 (4) | 9 (6) | 6 (0) | 12 (5) | 16 (5) | 9 (5) | 6 (1) |
|  | **2080** | 8 (7) | 20 (3) | 7 (5) | 8 (1) | 9 (7) | 19 (3) | 7 (5) | 8 (1) | 9 (5) | 19 (4) | 7 (4) | 8 (1) | 10 (7) | 18 (3) | 8 (5) | 7 (1) |
| **Romania** | **2020** | 19 (10) | 3 (18) | 12 (7) | 1 (8) | 19 (10) | 3 (18) | 12 (7) | 1 (8) | 19 (10) | 3 (18) | 12 (7) | 1 (8) | 19 (10) | 3 (18) | 12 (7) | 1 (8) |
|  | **2050** | 17 (10) | 5 (18) | 12 (7) | 1 (8) | 17 (11) | 5 (17) | 12 (8) | 1 (7) | 18 (12) | 4 (16) | 12 (9) | 1 (6) | 17 (11) | 5 (17) | 12 (8) | 1 (7) |
|  | **2080** | 20 (9) | 2 (19) | 12 (7) | 1 (8) | 20 (10) | 2 (18) | 12 (7) | 1 (8) | 19 (10) | 3 (18) | 12 (7) | 1 (8) | 19 (10) | 3 (18) | 12 (7) | 1 (8) |
| **Slovakia** | **2020** | 10 (19) | 1 (3) | 6 (12) | 0 (1) | 10 (19) | 1 (3) | 6 (12) | 0 (1) | 10 (19) | 1 (3) | 6 (12) | 0 (1) | 10 (19) | 1 (3) | 6 (12) | 0 (1) |
|  | **2050** | 8 (17) | 3 (5) | 5 (12) | 1 (1) | 8 (17) | 3 (5) | 5 (12) | 1 (1) | 8 (17) | 3 (5) | 5 (12) | 1 (1) | 7 (17) | 4 (5) | 5 (12) | 1 (1) |
|  | **2080** | 9 (18) | 2 (4) | 5 (12) | 1 (1) | 9 (18) | 2 (4) | 5 (12) | 1 (1) | 9 (18) | 2 (4) | 5 (12) | 1 (1) | 9 (18) | 2 (4) | 5 (12) | 1 (1) |
| **Slovenia** | **2020** | 23 (8) | 3 (3) | 12 (5) | 1 (1) | 23 (8) | 3 (3) | 12 (5) | 1 (1) | 22 (9) | 3 (2) | 12 (5) | 1 (1) | 23 (9) | 3 (2) | 12 (5) | 1 (1) |
|  | **2050** | 22 (8) | 4 (3) | 12 (5) | 1 (1) | 20 (7) | 5 (3) | 12 (5) | 1 (1) | 22 (8) | 4 (3) | 12 (5) | 1 (1) | 21 (6) | 5 (5) | 12 (5) | 1 (1) |
|  | **2080** | 23 (8) | 3 (3) | 12 (5) | 1 (1) | 23 (9) | 3 (2) | 12 (5) | 1 (1) | 23 (9) | 3 (2) | 12 (5) | 1 (1) | 23 (9) | 3 (2) | 12 (5) | 1 (1) |
| **Spain** | **2020** | 20 (22) | 16 (4) | 13 (12) | 7 (1) | 22 (22) | 14 (4) | 14 (12) | 6 (1) | 23 (22) | 13 (4) | 14 (12) | 6 (1) | 22 (21) | 14 (4) | 14 (12) | 6 (1) |
|  | **2050** | 15 (22) | 21 (4) | 13 (12) | 7 (1) | 15 (22) | 20 (4) | 13 (12) | 6 (1) | 14 (22) | 22 (4) | 12 (12) | 8 (1) | 15 (22) | 21 (4) | 13 (12) | 7 (1) |
|  | **2080** | 17 (22) | 19 (4) | 14 (12) | 6 (1) | 18 (22) | 18 (4) | 15 (12) | 5 (1) | 20 (22) | 16 (4) | 16 (12) | 4 (1) | 21 (22) | 15 (4) | 16 (12) | 4 (1) |
| **Sweden** | **2020** | 5 (17) | 1 (19) | 2 (12) | 0 (8) | 5 (17) | 1 (19) | 2 (12) | 0 (8) | 5 (17) | 1 (19) | 2 (12) | 0 (8) | 5 (17) | 1 (19) | 2 (12) | 0 (8) |
|  | **2050** | 5 (15) | 1 (21) | 2 (13) | 0 (7) | 5 (15) | 1 (21) | 2 (13) | 0 (7) | 5 (15) | 1 (21) | 2 (13) | 0 (7) | 5 (15) | 1 (21) | 2 (13) | 0 (7) |
|  | **2080** | 6 (16) | 0 (20) | 2 (13) | 0 (7) | 6 (16) | 0 (20) | 2 (13) | 0 (7) | 6 (16) | 0 (20) | 2 (13) | 0 (7) | 6 (17) | 0 (19) | 2 (14) | 0 (6) |
| **UK** | **2020** | 3 (6) | 3 (0) | 1 (2) | 1 (0) | 3 (6) | 3 (0) | 1 (2) | 1 (0) | 3 (6) | 3 (0) | 1 (2) | 1 (0) | 3 (6) | 3 (0) | 1 (2) | 1 (0) |
|  | **2050** | 3 (5) | 3 (1) | 1 (2) | 1 (0) | 3 (5) | 3 (1) | 1 (2) | 1 (0) | 3 (5) | 3 (1) | 1 (2) | 1 (0) | 3 (5) | 3 (1) | 1 (2) | 1 (0) |
|  | **2080** | 3 (5) | 3 (1) | 1 (2) | 1 (0) | 3 (6) | 3 (0) | 1 (2) | 1 (0) | 3 (6) | 3 (0) | 1 (2) | 1 (0) | 3 (6) | 3 (0) | 1 (2) | 1 (0) |

| ***Birds*** | |  |  |  |  |  |  |  |  |  |  |  |  |  |  |  |  |
| --- | --- | --- | --- | --- | --- | --- | --- | --- | --- | --- | --- | --- | --- | --- | --- | --- | --- |
| **Austria** | **2020** | 114 (113) | 109 (114) | 50 (51) | 62 (62) | 115 (116) | 110 (110) | 52 (53) | 61 (60) | 117 (112) | 107 (115) | 51 (50) | 61 (63) | 118 (113) | 106 (114) | 53 (51) | 60 (62) |
|  | **2050** | 130 (129) | 96 (98) | 64 (65) | 48 (48) | 134 (128) | 91 (98) | 67 (65) | 45 (48) | 132 (130) | 93 (97) | 64 (64) | 48 (49) | 131 (129) | 95 (98) | 63 (63) | 49 (50) |
|  | **2080** | 118 (118) | 110 (109) | 53 (53) | 60 (60) | 125 (122) | 103 (105) | 57 (56) | 56 (57) | 125 (121) | 101 (106) | 57 (56) | 55 (57) | 120 (116) | 105 (111) | 55 (53) | 57 (60) |
| **Belgium** | **2020** | 59 (58) | 121 (124) | 23 (23) | 61 (61) | 57 (51) | 122 (133) | 22 (20) | 62 (64) | 57 (51) | 123 (131) | 21 (20) | 63 (64) | 56 (48) | 123 (136) | 20 (18) | 63 (66) |
|  | **2050** | 77 (74) | 103 (110) | 34 (34) | 50 (50) | 74 (72) | 106 (112) | 33 (33) | 51 (51) | 72 (73) | 108 (111) | 31 (33) | 53 (51) | 66 (69) | 113 (115) | 30 (33) | 54 (51) |
|  | **2080** | 58 (57) | 124 (127) | 26 (25) | 58 (59) | 60 (59) | 121 (125) | 27 (26) | 57 (58) | 64 (60) | 116 (124) | 27 (28) | 56 (56) | 61 (59) | 120 (125) | 28 (27) | 56 (57) |
| **Bulgaria** | **2020** | 119 (115) | 125 (128) | 57 (56) | 58 (59) | 122 (118) | 123 (126) | 60 (59) | 55 (56) | 114 (114) | 131 (130) | 54 (55) | 61 (60) | 119 (116) | 125 (128) | 57 (55) | 58 (60) |
|  | **2050** | 115 (107) | 130 (137) | 62 (59) | 53 (56) | 114 (108) | 131 (136) | 59 (60) | 56 (55) | 112 (111) | 133 (133) | 60 (60) | 55 (55) | 110 (107) | 135 (137) | 59 (58) | 56 (57) |
|  | **2080** | 97 (91) | 148 (153) | 54 (49) | 61 (66) | 102 (96) | 143 (148) | 54 (52) | 61 (63) | 103 (100) | 141 (144) | 51 (48) | 64 (67) | 102 (100) | 143 (144) | 51 (48) | 64 (67) |
| **Czech**  **Republic** | **2020** | 108 (101) | 108 (117) | 53 (49) | 56 (62) | 109 (98) | 107 (120) | 53 (48) | 56 (63) | 104 (98) | 110 (120) | 52 (47) | 56 (64) | 106 (98) | 109 (120) | 53 (47) | 56 (64) |
|  | **2050** | 102 (91) | 115 (127) | 54 (46) | 56 (65) | 99 (95) | 118 (123) | 50 (46) | 60 (65) | 96 (87) | 121 (131) | 47 (43) | 63 (68) | 94 (86) | 122 (132) | 46 (43) | 63 (68) |
|  | **2080** | 92 (88) | 126 (130) | 48 (43) | 63 (68) | 96 (90) | 122 (128) | 48 (44) | 63 (67) | 101 (90) | 117 (128) | 49 (42) | 62 (69) | 98 (84) | 120 (134) | 47 (41) | 64 (70) |
| **Denmark** | **2020** | 105 (106) | 72 (71) | 47 (48) | 35 (34) | 106 (106) | 71 (71) | 49 (48) | 33 (34) | 106 (105) | 71 (72) | 49 (47) | 33 (35) | 106 (104) | 70 (73) | 49 (47) | 33 (35) |
|  | **2050** | 107 (111) | 71 (67) | 49 (52) | 34 (31) | 106 (110) | 70 (67) | 50 (52) | 33 (31) | 106 (110) | 72 (68) | 49 (52) | 34 (31) | 101 (106) | 76 (72) | 46 (49) | 37 (34) |
|  | **2080** | 65 (72) | 113 (106) | 23 (27) | 60 (56) | 76 (77) | 102 (101) | 30 (31) | 53 (52) | 78 (79) | 99 (99) | 32 (32) | 51 (51) | 77 (78) | 101 (100) | 31 (32) | 52 (51) |
| **Estonia** | **2020** | 77 (78) | 122 (120) | 34 (35) | 68 (67) | 76 (80) | 122 (119) | 33 (36) | 69 (66) | 75 (78) | 124 (121) | 33 (35) | 69 (67) | 79 (80) | 120 (119) | 34 (36) | 68 (66) |
|  | **2050** | 93 (95) | 106 (104) | 39 (41) | 63 (61) | 93 (95) | 106 (104) | 39 (41) | 63 (61) | 90 (96) | 109 (103) | 37 (42) | 65 (60) | 95 (96) | 104 (103) | 40 (41) | 62 (61) |
|  | **2080** | 78 (79) | 121 (119) | 32 (33) | 70 (68) | 83 (83) | 116 (116) | 36 (36) | 66 (66) | 87 (93) | 112 (106) | 38 (40) | 64 (62) | 90 (93) | 109 (106) | 39 (41) | 63 (61) |
| **Finland** | **2020** | 152 (129) | 67 (91) | 73 (60) | 37 (50) | 152 (128) | 67 (92) | 73 (59) | 37 (51) | 151 (127) | 68 (93) | 72 (59) | 38 (51) | 152 (126) | 67 (94) | 73 (58) | 37 (52) |
|  | **2050** | 141 (119) | 78 (101) | 65 (55) | 45 (55) | 140 (119) | 79 (101) | 65 (55) | 45 (55) | 139 (122) | 80 (98) | 64 (55) | 46 (55) | 138 (120) | 81 (100) | 63 (55) | 47 (55) |
|  | **2080** | 129 (110) | 90 (110) | 56 (49) | 54 (61) | 133 (118) | 86 (102) | 58 (53) | 52 (57) | 137 (120) | 82 (100) | 62 (54) | 48 (56) | 136 (121) | 83 (99) | 61 (55) | 49 (55) |
| **France** | **2020** | 79 (79) | 174 (175) | 34 (38) | 90 (87) | 81 (78) | 172 (177) | 35 (37) | 89 (88) | 78 (71) | 174 (183) | 32 (33) | 92 (92) | 77 (71) | 176 (184) | 32 (33) | 92 (92) |
|  | **2050** | 102 (98) | 152 (157) | 51 (51) | 74 (74) | 104 (100) | 150 (155) | 52 (52) | 73 (73) | 102 (98) | 151 (157) | 51 (51) | 73 (74) | 105 (99) | 148 (156) | 52 (51) | 72 (74) |
|  | **2080** | 86 (80) | 169 (175) | 49 (43) | 76 (82) | 92 (86) | 163 (169) | 50 (44) | 75 (81) | 89 (87) | 164 (168) | 46 (44) | 78 (81) | 88 (86) | 165 (169) | 45 (44) | 79 (81) |
| **Germany** | **2020** | 84 (77) | 146 (154) | 40 (34) | 72 (79) | 79 (74) | 150 (157) | 37 (33) | 75 (80) | 81 (76) | 148 (155) | 39 (34) | 73 (79) | 79 (76) | 150 (155) | 37 (34) | 75 (79) |
|  | **2050** | 88 (91) | 142 (141) | 40 (42) | 72 (72) | 91 (87) | 139 (144) | 40 (38) | 72 (75) | 90 (87) | 140 (144) | 40 (37) | 72 (76) | 90 (86) | 140 (145) | 41 (37) | 71 (76) |
|  | **2080** | 84 (87) | 149 (146) | 36 (38) | 79 (77) | 91 (93) | 142 (140) | 40 (42) | 75 (73) | 100 (100) | 131 (133) | 42 (44) | 71 (71) | 96 (96) | 135 (137) | 39 (43) | 74 (72) |
| **Greece** | **2020** | 84 (67) | 155 (173) | 42 (38) | 74 (78) | 82 (70) | 157 (170) | 42 (39) | 74 (77) | 82 (67) | 157 (173) | 40 (36) | 76 (80) | 85 (71) | 154 (168) | 43 (40) | 73 (76) |
|  | **2050** | 86 (74) | 153 (166) | 51 (47) | 65 (69) | 88 (76) | 151 (165) | 53 (49) | 63 (68) | 87 (76) | 152 (164) | 51 (48) | 65 (68) | 86 (75) | 153 (165) | 52 (48) | 64 (68) |
|  | **2080** | 61 (56) | 179 (185) | 37 (35) | 80 (82) | 65 (59) | 175 (182) | 40 (35) | 77 (82) | 62 (52) | 178 (189) | 35 (29) | 82 (88) | 68 (61) | 172 (180) | 40 (36) | 77 (81) |
| **Hungary** | **2020** | 65 (64) | 141 (143) | 39 (38) | 71 (73) | 67 (65) | 140 (142) | 40 (39) | 71 (72) | 69 (66) | 139 (141) | 39 (37) | 72 (74) | 67 (68) | 141 (139) | 39 (38) | 72 (73) |
|  | **2050** | 67 (68) | 140 (139) | 42 (42) | 69 (69) | 72 (69) | 136 (138) | 44 (43) | 67 (68) | 74 (70) | 134 (137) | 43 (41) | 68 (70) | 77 (71) | 131 (136) | 45 (43) | 66 (68) |
|  | **2080** | 57 (57) | 151 (150) | 34 (34) | 77 (77) | 62 (61) | 146 (146) | 38 (38) | 73 (73) | 66 (69) | 142 (138) | 39 (41) | 72 (70) | 68 (70) | 140 (137) | 39 (40) | 72 (71) |
| **Ireland** | **2020** | 44 (50) | 80 (79) | 17 (16) | 44 (45) | 45 (49) | 79 (80) | 17 (16) | 44 (45) | 44 (49) | 79 (80) | 16 (16) | 44 (45) | 45 (50) | 79 (79) | 17 (16) | 44 (45) |
|  | **2050** | 49 (50) | 77 (79) | 16 (15) | 45 (46) | 50 (50) | 76 (79) | 17 (15) | 44 (46) | 48 (50) | 78 (79) | 15 (15) | 46 (46) | 47 (50) | 79 (79) | 14 (15) | 47 (46) |
|  | **2080** | 48 (47) | 79 (82) | 15 (14) | 46 (47) | 50 (49) | 76 (80) | 15 (15) | 46 (46) | 50 (49) | 76 (80) | 16 (15) | 45 (46) | 47 (50) | 77 (79) | 14 (14) | 46 (47) |
| **Italy** | **2020** | 95 (64) | 139 (172) | 46 (27) | 64 (83) | 95 (66) | 140 (170) | 46 (27) | 64 (83) | 95 (66) | 140 (170) | 45 (28) | 65 (82) | 95 (66) | 138 (170) | 45 (29) | 64 (81) |
|  | **2050** | 87 (68) | 149 (168) | 45 (35) | 65 (75) | 90 (71) | 145 (165) | 48 (38) | 61 (72) | 88 (68) | 148 (168) | 46 (35) | 64 (75) | 88 (70) | 148 (166) | 46 (37) | 64 (73) |
|  | **2080** | 74 (62) | 160 (173) | 38 (36) | 72 (74) | 77 (62) | 158 (174) | 40 (34) | 70 (76) | 79 (60) | 156 (175) | 37 (30) | 73 (80) | 78 (60) | 156 (175) | 36 (31) | 73 (79) |
| **Latvia** | **2020** | 80 (83) | 118 (116) | 37 (36) | 67 (69) | 81 (83) | 116 (116) | 37 (36) | 66 (69) | 84 (84) | 114 (115) | 38 (37) | 66 (68) | 85 (83) | 112 (115) | 38 (36) | 65 (68) |
|  | **2050** | 99 (102) | 99 (97) | 46 (46) | 58 (59) | 98 (99) | 100 (100) | 46 (44) | 58 (61) | 96 (99) | 101 (100) | 45 (45) | 58 (60) | 97 (99) | 100 (100) | 45 (44) | 59 (61) |
|  | **2080** | 78 (82) | 121 (117) | 35 (36) | 70 (69) | 82 (83) | 117 (116) | 38 (37) | 67 (68) | 88 (88) | 111 (111) | 41 (40) | 64 (65) | 87 (89) | 112 (110) | 40 (41) | 65 (64) |
| **Lithuania** | **2020** | 83 (87) | 107 (105) | 36 (40) | 64 (61) | 83 (87) | 107 (104) | 37 (40) | 63 (60) | 84 (89) | 106 (103) | 37 (42) | 63 (59) | 85 (89) | 103 (103) | 37 (42) | 62 (59) |
|  | **2050** | 68 (66) | 123 (126) | 32 (31) | 68 (70) | 70 (68) | 121 (124) | 33 (31) | 67 (70) | 69 (68) | 122 (124) | 33 (32) | 67 (69) | 68 (66) | 123 (126) | 32 (30) | 68 (71) |
|  | **2080** | 60 (60) | 132 (133) | 26 (26) | 75 (76) | 61 (62) | 131 (131) | 25 (26) | 76 (76) | 68 (71) | 123 (121) | 29 (31) | 71 (70) | 61 (66) | 131 (127) | 26 (30) | 75 (72) |
| **Luxembourg** | **2020** | 62 (68) | 85 (85) | 25 (29) | 40 (37) | 62 (68) | 85 (85) | 25 (29) | 40 (37) | 60 (64) | 86 (89) | 23 (27) | 40 (39) | 59 (63) | 87 (89) | 23 (26) | 41 (39) |
|  | **2050** | 84 (87) | 62 (67) | 34 (35) | 28 (31) | 85 (88) | 63 (66) | 36 (37) | 28 (29) | 82 (85) | 64 (67) | 34 (35) | 29 (30) | 74 (78) | 72 (76) | 31 (32) | 34 (34) |
|  | **2080** | 49 (53) | 101 (101) | 18 (18) | 47 (48) | 57 (57) | 89 (97) | 20 (20) | 45 (46) | 64 (65) | 82 (89) | 20 (19) | 43 (47) | 59 (60) | 86 (94) | 19 (19) | 44 (47) |
| **Malta** | **2020** | 8 (7) | 6 (12) | 2 (1) | 3 (5) | 8 (7) | 6 (12) | 2 (1) | 3 (5) | 8 (7) | 6 (11) | 2 (1) | 3 (5) | 7 (6) | 6 (13) | 2 (1) | 3 (5) |
|  | **2050** | 10 (11) | 7 (8) | 4 (4) | 3 (3) | 10 (11) | 7 (8) | 4 (4) | 3 (3) | 10 (12) | 7 (7) | 4 (4) | 3 (3) | 10 (12) | 7 (8) | 4 (4) | 3 (3) |
|  | **2080** | 8 (9) | 10 (11) | 2 (2) | 5 (5) | 8 (9) | 10 (11) | 2 (2) | 5 (5) | 8 (9) | 9 (11) | 2 (3) | 3 (4) | 8 (8) | 8 (12) | 2 (2) | 4 (5) |
| **Netherlands** | **2020** | 40 (38) | 141 (146) | 17 (14) | 72 (76) | 40 (38) | 142 (146) | 17 (14) | 73 (76) | 39 (37) | 143 (147) | 16 (14) | 74 (76) | 39 (37) | 143 (147) | 16 (14) | 74 (76) |
|  | **2050** | 70 (64) | 113 (119) | 32 (28) | 58 (61) | 65 (62) | 117 (122) | 29 (28) | 61 (62) | 67 (64) | 116 (120) | 29 (28) | 61 (62) | 67 (63) | 115 (120) | 29 (28) | 60 (62) |
|  | **2080** | 60 (60) | 124 (124) | 28 (28) | 62 (62) | 61 (63) | 122 (121) | 28 (29) | 62 (61) | 61 (61) | 122 (123) | 25 (25) | 65 (65) | 61 (63) | 121 (121) | 28 (28) | 62 (62) |
| **Poland** | **2020** | 80 (78) | 143 (148) | 36 (35) | 79 (82) | 81 (77) | 143 (149) | 37 (35) | 78 (82) | 83 (79) | 141 (147) | 37 (37) | 78 (80) | 80 (79) | 144 (147) | 36 (36) | 79 (81) |
|  | **2050** | 71 (75) | 154 (151) | 35 (38) | 81 (79) | 71 (79) | 153 (147) | 35 (38) | 80 (79) | 72 (79) | 153 (147) | 36 (37) | 81 (80) | 72 (75) | 154 (151) | 35 (35) | 82 (82) |
|  | **2080** | 73 (71) | 153 (156) | 33 (34) | 85 (84) | 79 (78) | 147 (149) | 36 (35) | 81 (83) | 79 (86) | 147 (141) | 35 (40) | 82 (78) | 82 (85) | 145 (142) | 38 (39) | 80 (79) |
| **Portugal** | **2020** | 52 (54) | 139 (141) | 31 (34) | 61 (60) | 52 (53) | 139 (141) | 32 (33) | 60 (60) | 51 (54) | 140 (140) | 31 (34) | 61 (60) | 53 (52) | 138 (141) | 32 (32) | 60 (61) |
|  | **2050** | 58 (61) | 135 (134) | 36 (38) | 57 (56) | 58 (60) | 135 (135) | 36 (38) | 57 (56) | 58 (60) | 135 (134) | 35 (36) | 58 (57) | 55 (57) | 138 (137) | 33 (35) | 60 (58) |
|  | **2080** | 54 (58) | 139 (137) | 33 (35) | 60 (59) | 53 (55) | 140 (140) | 33 (34) | 60 (60) | 55 (55) | 138 (140) | 34 (33) | 59 (61) | 52 (54) | 141 (141) | 30 (31) | 63 (63) |
| **Romania** | **2020** | 123 (111) | 123 (134) | 63 (57) | 62 (68) | 123 (108) | 123 (137) | 63 (53) | 62 (72) | 121 (111) | 125 (134) | 60 (55) | 65 (70) | 123 (111) | 123 (134) | 62 (55) | 63 (70) |
|  | **2050** | 100 (85) | 146 (160) | 55 (48) | 70 (77) | 104 (86) | 142 (159) | 57 (48) | 68 (77) | 105 (82) | 141 (163) | 55 (45) | 70 (80) | 103 (84) | 142 (161) | 55 (47) | 69 (78) |
|  | **2080** | 93 (75) | 154 (170) | 47 (40) | 78 (85) | 100 (78) | 147 (167) | 51 (42) | 74 (83) | 105 (84) | 139 (161) | 51 (43) | 72 (82) | 102 (89) | 143 (156) | 50 (43) | 74 (82) |
| **Slovakia** | **2020** | 105 (86) | 111 (131) | 51 (44) | 62 (69) | 104 (87) | 113 (130) | 50 (44) | 63 (69) | 106 (90) | 111 (127) | 50 (44) | 63 (69) | 108 (88) | 109 (129) | 52 (42) | 61 (71) |
|  | **2050** | 93 (83) | 124 (134) | 51 (46) | 62 (67) | 94 (85) | 123 (132) | 52 (48) | 61 (65) | 91 (81) | 126 (136) | 50 (44) | 63 (69) | 90 (81) | 127 (136) | 50 (45) | 63 (68) |
|  | **2080** | 86 (70) | 131 (147) | 45 (37) | 68 (76) | 92 (80) | 125 (137) | 47 (41) | 66 (72) | 91 (78) | 126 (139) | 44 (36) | 69 (77) | 91 (79) | 126 (138) | 45 (38) | 68 (75) |
| **Slovenia** | **2020** | 105 (94) | 107 (118) | 49 (44) | 48 (52) | 107 (94) | 105 (118) | 51 (44) | 46 (52) | 104 (94) | 108 (118) | 48 (45) | 49 (51) | 102 (93) | 110 (119) | 47 (45) | 50 (51) |
|  | **2050** | 119 (115) | 94 (101) | 55 (54) | 42 (45) | 123 (117) | 90 (99) | 56 (55) | 41 (44) | 119 (114) | 94 (102) | 54 (53) | 43 (46) | 119 (113) | 94 (103) | 54 (52) | 43 (47) |
|  | **2080** | 101 (96) | 114 (120) | 46 (45) | 52 (54) | 110 (103) | 104 (113) | 50 (48) | 48 (51) | 111 (102) | 103 (113) | 49 (47) | 49 (51) | 113 (104) | 101 (110) | 49 (49) | 49 (49) |
| **Spain** | **2020** | 83 (60) | 153 (177) | 43 (33) | 73 (84) | 87 (63) | 149 (174) | 45 (35) | 71 (82) | 82 (59) | 154 (178) | 41 (32) | 75 (85) | 81 (59) | 154 (178) | 43 (32) | 72 (85) |
|  | **2050** | 85 (65) | 153 (172) | 48 (39) | 69 (78) | 88 (65) | 150 (172) | 50 (39) | 67 (78) | 85 (65) | 153 (172) | 48 (40) | 69 (77) | 86 (63) | 152 (174) | 48 (38) | 69 (79) |
|  | **2080** | 71 (58) | 167 (179) | 40 (34) | 77 (83) | 76 (60) | 161 (177) | 45 (35) | 72 (82) | 76 (59) | 161 (178) | 43 (35) | 74 (82) | 76 (61) | 162 (176) | 41 (36) | 76 (81) |
| **Sweden** | **2020** | 130 (110) | 93 (116) | 56 (47) | 56 (65) | 129 (109) | 93 (117) | 55 (47) | 56 (65) | 134 (112) | 90 (114) | 59 (48) | 53 (64) | 134 (111) | 90 (115) | 59 (47) | 53 (65) |
|  | **2050** | 139 (118) | 84 (107) | 61 (51) | 51 (61) | 138 (120) | 84 (105) | 60 (51) | 51 (61) | 140 (120) | 82 (105) | 62 (52) | 49 (60) | 138 (117) | 85 (108) | 60 (51) | 51 (61) |
|  | **2080** | 123 (102) | 103 (124) | 51 (44) | 62 (69) | 128 (109) | 98 (117) | 54 (46) | 59 (67) | 137 (117) | 88 (109) | 59 (52) | 53 (61) | 135 (113) | 90 (113) | 57 (50) | 55 (63) |
| **UK** | **2020** | 80 (69) | 109 (124) | 38 (30) | 50 (62) | 77 (68) | 112 (125) | 37 (29) | 51 (63) | 80 (67) | 109 (126) | 37 (28) | 51 (64) | 78 (70) | 110 (123) | 36 (30) | 52 (62) |
|  | **2050** | 89 (76) | 102 (117) | 37 (30) | 53 (62) | 87 (75) | 103 (117) | 37 (30) | 53 (61) | 86 (74) | 106 (118) | 35 (29) | 56 (62) | 88 (75) | 104 (118) | 36 (30) | 55 (62) |
|  | **2080** | 76 (69) | 117 (124) | 30 (27) | 62 (65) | 83 (72) | 109 (121) | 34 (26) | 57 (66) | 86 (76) | 106 (117) | 36 (28) | 55 (64) | 84 (74) | 108 (119) | 35 (26) | 56 (66) |

| ***Mammals*** |  |  |  |  |  |  |  |  |  |  |  |  |  |  |  |  |
| --- | --- | --- | --- | --- | --- | --- | --- | --- | --- | --- | --- | --- | --- | --- | --- | --- |

| **Austria** | **2020** | 49 (50) | 37 (37) | 21 (21) | 17 (17) | 50 (51) | 36 (36) | 21 (21) | 17 (17) | 49 (51) | 36 (36) | 21 (21) | 17 (17) | 49 (50) | 37 (37) | 21 (21) | 17 (17) |
| --- | --- | --- | --- | --- | --- | --- | --- | --- | --- | --- | --- | --- | --- | --- | --- | --- | --- |
|  | **2050** | 50 (51) | 36 (36) | 20 (22) | 18 (16) | 53 (51) | 33 (36) | 22 (23) | 16 (15) | 53 (49) | 33 (37) | 23 (22) | 15 (16) | 53 (51) | 33 (36) | 23 (22) | 15 (16) |
|  | **2080** | 44 (42) | 42 (45) | 18 (16) | 20 (22) | 51 (47) | 35 (40) | 22 (19) | 16 (19) | 55 (48) | 31 (39) | 23 (21) | 15 (17) | 53 (49) | 33 (38) | 22 (21) | 16 (17) |
| **Belgium** | **2020** | 39 (31) | 24 (31) | 19 (15) | 7 (11) | 38 (30) | 25 (33) | 19 (15) | 7 (11) | 38 (30) | 25 (33) | 18 (15) | 8 (11) | 36 (29) | 27 (34) | 18 (15) | 8 (11) |
|  | **2050** | 26 (23) | 37 (40) | 9 (9) | 17 (17) | 29 (25) | 34 (38) | 10 (10) | 16 (16) | 29 (25) | 34 (38) | 10 (10) | 16 (16) | 26 (21) | 37 (42) | 10 (9) | 16 (17) |
|  | **2080** | 16 (16) | 47 (47) | 8 (8) | 18 (18) | 20 (19) | 43 (44) | 9 (9) | 17 (17) | 21 (19) | 42 (44) | 8 (9) | 18 (17) | 20 (19) | 43 (44) | 8 (8) | 18 (18) |
| **Bulgaria** | **2020** | 53 (46) | 33 (40) | 27 (20) | 13 (20) | 54 (46) | 32 (40) | 27 (20) | 13 (20) | 55 (46) | 31 (39) | 28 (20) | 12 (20) | 54 (48) | 32 (38) | 28 (22) | 12 (18) |
|  | **2050** | 40 (33) | 46 (53) | 22 (18) | 18 (22) | 40 (35) | 46 (51) | 21 (18) | 19 (22) | 43 (36) | 43 (50) | 23 (18) | 17 (22) | 45 (35) | 41 (51) | 23 (18) | 17 (22) |
|  | **2080** | 32 (29) | 54 (57) | 15 (13) | 25 (27) | 39 (35) | 47 (51) | 19 (16) | 21 (24) | 38 (32) | 48 (54) | 18 (14) | 22 (26) | 40 (36) | 46 (50) | 19 (15) | 21 (25) |
| **Czech Republic** | **2020** | 52 (43) | 28 (37) | 24 (19) | 12 (17) | 50 (43) | 30 (37) | 24 (19) | 12 (17) | 51 (44) | 29 (36) | 24 (19) | 12 (17) | 50 (44) | 30 (36) | 24 (19) | 12 (17) |
|  | **2050** | 44 (36) | 36 (44) | 21 (17) | 15 (19) | 43 (39) | 37 (41) | 22 (18) | 14 (18) | 41 (38) | 39 (42) | 20 (18) | 16 (18) | 41 (39) | 39 (41) | 21 (19) | 15 (17) |
|  | **2080** | 41 (31) | 39 (49) | 18 (13) | 18 (23) | 44 (34) | 36 (46) | 18 (13) | 18 (23) | 48 (44) | 32 (36) | 22 (20) | 14 (16) | 46 (42) | 34 (38) | 20 (19) | 16 (17) |
| **Denmark** | **2020** | 36 (39) | 17 (14) | 13 (14) | 6 (5) | 37 (39) | 16 (14) | 14 (14) | 5 (5) | 36 (39) | 17 (14) | 13 (14) | 6 (5) | 36 (39) | 16 (14) | 13 (14) | 5 (5) |
|  | **2050** | 32 (35) | 21 (18) | 12 (13) | 7 (6) | 33 (35) | 20 (18) | 13 (13) | 6 (6) | 34 (35) | 19 (18) | 14 (13) | 5 (6) | 33 (34) | 20 (19) | 13 (13) | 6 (6) |
|  | **2080** | 21 (22) | 32 (31) | 6 (6) | 13 (13) | 26 (25) | 27 (28) | 8 (8) | 11 (11) | 27 (28) | 26 (25) | 8 (8) | 11 (11) | 26 (26) | 27 (27) | 8 (8) | 11 (11) |
| **Estonia** | **2020** | 25 (26) | 29 (28) | 10 (10) | 11 (11) | 25 (26) | 29 (28) | 10 (10) | 11 (11) | 25 (26) | 29 (28) | 10 (10) | 11 (11) | 25 (26) | 29 (28) | 10 (10) | 11 (11) |
|  | **2050** | 27 (27) | 27 (27) | 9 (9) | 12 (12) | 27 (27) | 27 (27) | 9 (9) | 12 (12) | 27 (27) | 26 (27) | 9 (9) | 12 (12) | 26 (26) | 28 (28) | 9 (9) | 12 (12) |
|  | **2080** | 20 (20) | 34 (34) | 7 (7) | 14 (14) | 22 (21) | 32 (33) | 8 (8) | 13 (13) | 22 (24) | 32 (30) | 8 (8) | 13 (13) | 22 (22) | 32 (32) | 8 (8) | 13 (13) |
| **Finland** | **2020** | 38 (31) | 20 (27) | 13 (11) | 6 (8) | 38 (31) | 20 (27) | 13 (11) | 6 (8) | 39 (31) | 19 (27) | 13 (11) | 6 (8) | 39 (31) | 19 (27) | 13 (11) | 6 (8) |
|  | **2050** | 38 (30) | 20 (28) | 12 (11) | 7 (8) | 38 (31) | 20 (27) | 12 (11) | 7 (8) | 37 (31) | 21 (27) | 12 (11) | 7 (8) | 37 (31) | 21 (27) | 12 (11) | 7 (8) |
|  | **2080** | 34 (27) | 24 (31) | 12 (10) | 7 (9) | 34 (28) | 24 (30) | 12 (10) | 7 (9) | 34 (30) | 24 (28) | 12 (11) | 7 (8) | 34 (30) | 24 (28) | 12 (11) | 7 (8) |
| **France** | **2020** | 30 (27) | 63 (65) | 13 (13) | 30 (29) | 33 (27) | 60 (65) | 14 (13) | 29 (29) | 31 (27) | 62 (65) | 13 (13) | 30 (29) | 33 (27) | 60 (65) | 15 (13) | 28 (29) |
|  | **2050** | 26 (21) | 66 (71) | 15 (12) | 27 (30) | 26 (22) | 66 (70) | 15 (12) | 27 (30) | 24 (23) | 68 (69) | 14 (13) | 28 (29) | 25 (24) | 67 (68) | 14 (13) | 28 (29) |
|  | **2080** | 20 (20) | 73 (73) | 13 (13) | 30 (30) | 22 (22) | 71 (70) | 14 (13) | 29 (29) | 24 (24) | 68 (68) | 14 (13) | 28 (29) | 25 (23) | 67 (69) | 14 (13) | 28 (29) |
| **Germany** | **2020** | 35 (35) | 48 (49) | 15 (15) | 21 (21) | 35 (35) | 48 (49) | 15 (15) | 21 (21) | 35 (36) | 48 (48) | 15 (16) | 21 (20) | 35 (35) | 48 (49) | 15 (16) | 21 (20) |
|  | **2050** | 31 (31) | 52 (53) | 12 (11) | 24 (25) | 30 (30) | 52 (54) | 12 (11) | 24 (25) | 29 (29) | 53 (55) | 12 (11) | 24 (25) | 30 (28) | 53 (56) | 12 (11) | 24 (25) |
|  | **2080** | 26 (24) | 57 (60) | 9 (10) | 27 (26) | 28 (27) | 54 (56) | 8 (8) | 28 (28) | 33 (32) | 50 (52) | 10 (9) | 26 (27) | 31 (30) | 52 (54) | 9 (9) | 27 (27) |
| **Greece** | **2020** | 13 (7) | 65 (71) | 7 (3) | 31 (35) | 16 (7) | 62 (71) | 8 (3) | 30 (35) | 14 (8) | 64 (70) | 7 (3) | 31 (35) | 16 (7) | 62 (71) | 8 (3) | 30 (35) |
|  | **2050** | 29 (22) | 49 (56) | 11 (6) | 27 (32) | 29 (23) | 49 (55) | 11 (6) | 27 (32) | 29 (23) | 49 (55) | 11 (6) | 27 (32) | 29 (28) | 49 (50) | 11 (9) | 27 (29) |
|  | **2080** | 19 (16) | 59 (62) | 8 (6) | 30 (32) | 21 (16) | 57 (62) | 9 (6) | 29 (32) | 19 (16) | 59 (62) | 10 (7) | 28 (31) | 22 (18) | 56 (60) | 10 (8) | 28 (30) |
| **Hungary** | **2020** | 28 (24) | 57 (61) | 10 (6) | 30 (33) | 28 (24) | 56 (61) | 10 (6) | 30 (33) | 28 (25) | 57 (60) | 10 (7) | 30 (32) | 29 (26) | 56 (59) | 10 (7) | 30 (32) |
|  | **2050** | 33 (31) | 52 (54) | 13 (13) | 27 (26) | 35 (33) | 51 (52) | 14 (12) | 26 (27) | 36 (38) | 50 (48) | 13 (16) | 27 (24) | 40 (39) | 45 (46) | 17 (16) | 22 (23) |
|  | **2080** | 26 (26) | 60 (59) | 11 (11) | 29 (28) | 30 (29) | 56 (56) | 12 (13) | 28 (26) | 36 (36) | 50 (50) | 15 (16) | 25 (24) | 37 (37) | 49 (49) | 16 (16) | 24 (24) |
| **Ireland** | **2020** | 12 (19) | 16 (9) | 6 (9) | 7 (4) | 11 (19) | 17 (9) | 6 (9) | 7 (4) | 11 (19) | 17 (9) | 6 (9) | 7 (4) | 11 (19) | 17 (9) | 6 (9) | 7 (4) |
|  | **2050** | 13 (15) | 15 (13) | 6 (8) | 7 (5) | 13 (15) | 15 (13) | 6 (8) | 7 (5) | 13 (15) | 15 (13) | 6 (8) | 7 (5) | 13 (15) | 15 (13) | 6 (8) | 7 (5) |
|  | **2080** | 10 (11) | 18 (17) | 4 (5) | 9 (8) | 11 (12) | 17 (16) | 5 (6) | 8 (7) | 13 (14) | 15 (14) | 6 (7) | 7 (6) | 12 (14) | 16 (14) | 6 (7) | 7 (6) |
| **Italy** | **2020** | 49 (34) | 41 (57) | 25 (16) | 15 (25) | 52 (33) | 39 (58) | 26 (16) | 15 (25) | 49 (31) | 42 (60) | 25 (16) | 16 (25) | 51 (34) | 40 (57) | 26 (16) | 15 (25) |
|  | **2050** | 40 (26) | 51 (65) | 20 (12) | 21 (29) | 40 (26) | 51 (65) | 19 (11) | 22 (30) | 39 (26) | 52 (65) | 20 (12) | 21 (29) | 44 (28) | 47 (63) | 22 (12) | 19 (29) |
|  | **2080** | 30 (19) | 61 (72) | 14 (7) | 27 (34) | 33 (19) | 58 (72) | 17 (7) | 24 (34) | 34 (24) | 57 (67) | 18 (12) | 23 (29) | 35 (23) | 56 (68) | 19 (11) | 22 (30) |
| **Latvia** | **2020** | 29 (29) | 32 (32) | 13 (13) | 12 (12) | 29 (29) | 32 (32) | 13 (13) | 12 (12) | 29 (29) | 32 (32) | 13 (13) | 12 (12) | 29 (29) | 32 (32) | 13 (13) | 12 (12) |
|  | **2050** | 33 (33) | 28 (28) | 12 (12) | 13 (13) | 33 (33) | 28 (28) | 12 (12) | 13 (13) | 33 (33) | 28 (28) | 12 (12) | 13 (13) | 33 (34) | 28 (27) | 12 (12) | 13 (13) |
|  | **2080** | 24 (24) | 37 (37) | 10 (10) | 15 (15) | 23 (23) | 38 (38) | 10 (10) | 15 (15) | 28 (24) | 33 (37) | 11 (10) | 14 (15) | 25 (24) | 36 (37) | 11 (10) | 14 (15) |
| **Lithuania** | **2020** | 34 (33) | 25 (26) | 15 (14) | 8 (9) | 35 (33) | 24 (26) | 16 (14) | 7 (9) | 34 (33) | 25 (26) | 15 (14) | 8 (9) | 36 (33) | 23 (26) | 16 (14) | 7 (9) |
|  | **2050** | 26 (26) | 32 (33) | 13 (13) | 10 (10) | 29 (28) | 29 (31) | 14 (14) | 9 (9) | 29 (28) | 29 (31) | 13 (14) | 10 (9) | 26 (25) | 32 (34) | 12 (12) | 11 (11) |
|  | **2080** | 19 (20) | 40 (39) | 7 (8) | 16 (15) | 21 (21) | 38 (38) | 8 (8) | 15 (15) | 21 (22) | 37 (37) | 8 (9) | 15 (14) | 22 (21) | 37 (38) | 8 (8) | 15 (15) |
| **Luxembourg** | **2020** | 38 (37) | 23 (24) | 20 (19) | 5 (6) | 37 (36) | 23 (25) | 19 (19) | 5 (6) | 38 (38) | 23 (23) | 20 (19) | 5 (6) | 37 (36) | 24 (25) | 19 (19) | 6 (6) |
|  | **2050** | 34 (32) | 27 (29) | 12 (11) | 13 (14) | 38 (37) | 23 (24) | 16 (15) | 9 (10) | 37 (37) | 24 (24) | 16 (16) | 9 (9) | 35 (34) | 25 (27) | 14 (13) | 10 (12) |
|  | **2080** | 17 (17) | 44 (44) | 8 (8) | 17 (17) | 22 (22) | 39 (39) | 9 (9) | 16 (16) | 28 (27) | 33 (34) | 10 (9) | 15 (16) | 27 (26) | 34 (35) | 9 (9) | 16 (16) |
| **Malta** | **2020** | 5 (7) | 5 (8) | 2 (3) | 3 (5) | 5 (6) | 5 (9) | 2 (3) | 3 (5) | 5 (7) | 5 (8) | 2 (3) | 3 (5) | 5 (6) | 5 (9) | 2 (3) | 3 (5) |
|  | **2050** | 7 (10) | 4 (5) | 3 (6) | 2 (2) | 7 (10) | 4 (5) | 3 (6) | 2 (2) | 9 (10) | 4 (5) | 5 (6) | 2 (2) | 8 (10) | 5 (5) | 4 (6) | 2 (2) |
|  | **2080** | 8 (10) | 4 (5) | 3 (4) | 3 (4) | 6 (8) | 4 (6) | 2 (3) | 3 (4) | 8 (10) | 3 (5) | 3 (4) | 2 (4) | 8 (9) | 5 (6) | 3 (4) | 3 (4) |
| **Netherlands** | **2020** | 18 (19) | 42 (41) | 10 (10) | 15 (15) | 19 (19) | 41 (41) | 11 (10) | 14 (15) | 19 (19) | 41 (41) | 11 (10) | 14 (15) | 19 (19) | 41 (41) | 11 (10) | 14 (15) |
|  | **2050** | 23 (20) | 37 (40) | 10 (9) | 15 (16) | 21 (22) | 38 (38) | 10 (11) | 14 (14) | 22 (23) | 38 (37) | 11 (11) | 14 (14) | 22 (21) | 38 (39) | 11 (11) | 14 (14) |
|  | **2080** | 17 (16) | 43 (44) | 8 (7) | 17 (18) | 17 (17) | 43 (43) | 8 (7) | 17 (18) | 21 (21) | 39 (39) | 9 (9) | 16 (16) | 20 (19) | 39 (40) | 8 (8) | 16 (16) |
| **Poland** | **2020** | 44 (43) | 36 (37) | 23 (23) | 13 (13) | 45 (44) | 35 (36) | 23 (24) | 13 (12) | 46 (45) | 34 (35) | 24 (24) | 12 (12) | 46 (44) | 34 (36) | 24 (24) | 12 (12) |
|  | **2050** | 35 (32) | 45 (48) | 18 (16) | 18 (20) | 36 (33) | 44 (47) | 18 (17) | 18 (19) | 35 (34) | 45 (46) | 18 (18) | 18 (18) | 35 (33) | 45 (47) | 18 (17) | 18 (19) |
|  | **2080** | 27 (25) | 53 (55) | 14 (12) | 22 (24) | 30 (26) | 50 (54) | 15 (14) | 21 (22) | 34 (31) | 46 (49) | 17 (16) | 19 (20) | 32 (30) | 48 (50) | 17 (16) | 19 (20) |
| **Portugal** | **2020** | 11 (13) | 49 (47) | 5 (5) | 25 (25) | 12 (13) | 48 (47) | 5 (5) | 25 (25) | 11 (12) | 49 (48) | 5 (4) | 25 (26) | 15 (15) | 45 (45) | 7 (7) | 23 (23) |
|  | **2050** | 8 (6) | 52 (54) | 2 (2) | 28 (28) | 7 (6) | 53 (54) | 2 (2) | 28 (28) | 7 (5) | 53 (55) | 2 (1) | 28 (29) | 10 (8) | 50 (52) | 4 (3) | 26 (27) |
|  | **2080** | 8 (7) | 52 (53) | 4 (4) | 26 (26) | 8 (8) | 52 (52) | 4 (4) | 26 (26) | 6 (7) | 54 (53) | 3 (3) | 27 (27) | 6 (8) | 54 (52) | 3 (4) | 27 (26) |
| **Romania** | **2020** | 57 (39) | 27 (46) | 30 (22) | 10 (18) | 55 (39) | 29 (46) | 29 (22) | 11 (18) | 56 (43) | 28 (42) | 29 (24) | 11 (16) | 56 (42) | 27 (43) | 29 (23) | 10 (17) |
|  | **2050** | 50 (26) | 34 (59) | 23 (15) | 17 (25) | 49 (26) | 35 (59) | 23 (15) | 17 (25) | 49 (31) | 35 (54) | 24 (17) | 16 (23) | 49 (31) | 35 (54) | 22 (17) | 18 (23) |
|  | **2080** | 37 (26) | 48 (59) | 16 (13) | 24 (27) | 43 (27) | 42 (58) | 20 (13) | 20 (27) | 51 (33) | 33 (52) | 24 (17) | 16 (23) | 51 (35) | 33 (50) | 25 (18) | 15 (22) |
| **Slovakia** | **2020** | 53 (44) | 37 (47) | 23 (16) | 15 (23) | 53 (42) | 37 (48) | 23 (16) | 15 (23) | 54 (45) | 36 (46) | 23 (16) | 16 (23) | 56 (45) | 35 (46) | 24 (16) | 15 (23) |
|  | **2050** | 49 (33) | 42 (58) | 21 (16) | 18 (23) | 51 (34) | 40 (57) | 23 (16) | 16 (23) | 49 (37) | 41 (54) | 21 (17) | 17 (22) | 51 (38) | 40 (53) | 23 (17) | 16 (22) |
|  | **2080** | 41 (30) | 50 (61) | 17 (12) | 22 (27) | 42 (35) | 48 (56) | 18 (14) | 21 (25) | 47 (37) | 43 (54) | 19 (15) | 19 (24) | 48 (39) | 42 (52) | 20 (16) | 19 (23) |
| **Slovenia** | **2020** | 34 (30) | 45 (49) | 12 (13) | 27 (26) | 34 (29) | 45 (50) | 12 (13) | 27 (26) | 31 (31) | 47 (48) | 12 (13) | 27 (26) | 34 (31) | 45 (48) | 12 (13) | 27 (26) |
|  | **2050** | 40 (32) | 39 (47) | 16 (14) | 23 (25) | 42 (34) | 37 (45) | 17 (15) | 22 (24) | 40 (33) | 39 (46) | 15 (13) | 24 (26) | 39 (32) | 40 (47) | 15 (13) | 24 (26) |
|  | **2080** | 32 (30) | 47 (49) | 14 (13) | 25 (26) | 35 (32) | 44 (47) | 14 (13) | 25 (26) | 36 (31) | 43 (48) | 14 (13) | 25 (26) | 35 (30) | 43 (49) | 14 (13) | 25 (26) |
| **Spain** | **2020** | 29 (11) | 50 (68) | 18 (4) | 18 (32) | 27 (10) | 52 (69) | 16 (4) | 20 (32) | 27 (10) | 52 (69) | 15 (3) | 21 (33) | 29 (12) | 50 (67) | 17 (6) | 19 (30) |
|  | **2050** | 22 (9) | 57 (70) | 13 (4) | 23 (32) | 21 (9) | 58 (70) | 12 (4) | 24 (32) | 19 (10) | 60 (69) | 11 (4) | 25 (32) | 23 (12) | 56 (67) | 14 (6) | 22 (30) |
|  | **2080** | 16 (10) | 63 (69) | 10 (5) | 26 (31) | 16 (10) | 63 (69) | 10 (5) | 26 (31) | 24 (11) | 55 (68) | 16 (6) | 20 (30) | 26 (13) | 53 (66) | 16 (7) | 20 (29) |
| **Sweden** | **2020** | 40 (36) | 24 (28) | 18 (17) | 8 (9) | 40 (36) | 24 (28) | 18 (17) | 8 (9) | 40 (36) | 24 (28) | 18 (17) | 8 (9) | 40 (36) | 24 (28) | 18 (17) | 8 (9) |
|  | **2050** | 44 (39) | 20 (25) | 19 (18) | 7 (8) | 44 (40) | 20 (24) | 19 (18) | 7 (8) | 44 (41) | 20 (23) | 19 (18) | 7 (8) | 44 (39) | 20 (25) | 19 (18) | 7 (8) |
|  | **2080** | 38 (35) | 26 (29) | 15 (15) | 11 (11) | 41 (36) | 23 (28) | 17 (16) | 9 (10) | 43 (38) | 21 (26) | 18 (17) | 8 (9) | 42 (37) | 22 (27) | 17 (17) | 9 (9) |
| **UK** | **2020** | 26 (18) | 21 (30) | 14 (11) | 7 (10) | 26 (18) | 21 (29) | 14 (11) | 7 (10) | 26 (20) | 21 (28) | 14 (11) | 7 (10) | 26 (20) | 21 (28) | 14 (11) | 7 (10) |
|  | **2050** | 23 (20) | 25 (28) | 11 (10) | 10 (11) | 24 (20) | 23 (28) | 12 (10) | 8 (11) | 24 (20) | 24 (28) | 12 (10) | 9 (11) | 24 (20) | 24 (28) | 12 (10) | 9 (11) |
|  | **2080** | 19 (14) | 29 (34) | 8 (5) | 13 (16) | 20 (15) | 28 (33) | 9 (5) | 12 (16) | 20 (19) | 28 (29) | 9 (9) | 12 (12) | 21 (19) | 27 (29) | 9 (9) | 12 (12) |

| ***Plants*** | |  |  |  |  |  |  |  |  |  |  |  |  |  |  |  |  |
| --- | --- | --- | --- | --- | --- | --- | --- | --- | --- | --- | --- | --- | --- | --- | --- | --- | --- |
| **Austria** | **2020** | 338 (312) | 316 (348) | 6 (8) | 8 (7) | 337 (310) | 318 (350) | 6 (8) | 8 (7) | 333 (306) | 323 (353) | 6 (8) | 8 (7) | 337 (302) | 319 (358) | 6 (8) | 8 (7) |
|  | **2050** | 368 (332) | 284 (328) | 7 (6) | 7 (9) | 380 (347) | 275 (312) | 8 (8) | 6 (7) | 372 (326) | 279 (332) | 8 (7) | 6 (8) | 378 (334) | 277 (324) | 8 (7) | 6 (8) |
|  | **2080** | 281 (258) | 376 (401) | 6 (6) | 9 (9) | 313 (278) | 344 (380) | 6 (6) | 8 (9) | 328 (290) | 328 (370) | 6 (6) | 8 (9) | 325 (288) | 332 (373) | 6 (6) | 8 (9) |
| **Belgium** | **2020** | 119 (121) | 269 (269) | 6 (5) | 2 (3) | 120 (120) | 267 (270) | 6 (5) | 2 (3) | 121 (119) | 268 (271) | 6 (5) | 2 (3) | 117 (117) | 271 (273) | 6 (5) | 2 (3) |
|  | **2050** | 133 (135) | 257 (256) | 7 (7) | 1 (1) | 131 (132) | 259 (259) | 7 (7) | 1 (1) | 129 (130) | 260 (261) | 7 (7) | 1 (1) | 127 (122) | 263 (269) | 6 (6) | 1 (2) |
|  | **2080** | 128 (122) | 263 (269) | 6 (5) | 2 (3) | 134 (132) | 256 (259) | 6 (6) | 2 (2) | 134 (129) | 256 (262) | 7 (6) | 1 (2) | 135 (129) | 256 (262) | 7 (6) | 1 (2) |
| **Bulgaria** | **2020** | 293 (336) | 363 (293) | 6 (8) | 8 (5) | 295 (343) | 359 (288) | 6 (8) | 8 (5) | 282 (331) | 374 (300) | 4 (8) | 10 (5) | 296 (340) | 359 (291) | 5 (8) | 8 (5) |
|  | **2050** | 259 (335) | 397 (296) | 5 (9) | 9 (4) | 266 (345) | 391 (285) | 5 (9) | 9 (4) | 269 (333) | 388 (297) | 4 (9) | 10 (4) | 280 (335) | 378 (296) | 5 (9) | 9 (4) |
|  | **2080** | 208 (275) | 451 (356) | 4 (9) | 10 (4) | 222 (292) | 437 (339) | 5 (9) | 9 (4) | 224 (304) | 435 (327) | 3 (9) | 11 (4) | 241 (305) | 417 (326) | 5 (9) | 9 (4) |
| **Czech Republic** | **2020** | 312 (279) | 205 (381) | 7 (4) | 5 (10) | 306 (278) | 210 (382) | 7 (5) | 5 (9) | 310 (272) | 206 (386) | 7 (4) | 5 (10) | 309 (282) | 207 (378) | 7 (4) | 5 (10) |
|  | **2050** | 284 (231) | 234 (429) | 8 (4) | 4 (10) | 290 (234) | 226 (426) | 8 (4) | 4 (10) | 293 (234) | 222 (425) | 8 (4) | 4 (10) | 288 (241) | 228 (418) | 8 (4) | 4 (10) |
|  | **2080** | 221 (169) | 296 (491) | 5 (4) | 7 (10) | 245 (191) | 271 (469) | 6 (4) | 6 (10) | 255 (190) | 263 (469) | 6 (4) | 6 (10) | 252 (205) | 265 (455) | 6 (4) | 6 (10) |
| **Denmark** | **2020** | 186 (262) | 142 (259) | 2 (6) | 4 (6) | 186 (263) | 142 (258) | 2 (5) | 4 (7) | 181 (269) | 147 (252) | 2 (6) | 4 (6) | 182 (270) | 146 (251) | 2 (6) | 4 (6) |
|  | **2050** | 130 (238) | 198 (282) | 2 (7) | 4 (5) | 135 (249) | 193 (271) | 2 (7) | 4 (5) | 132 (245) | 195 (275) | 2 (7) | 4 (5) | 126 (248) | 201 (272) | 2 (7) | 4 (5) |
|  | **2080** | 83 (201) | 245 (320) | 2 (6) | 4 (6) | 87 (218) | 240 (302) | 2 (6) | 4 (6) | 94 (238) | 233 (283) | 1 (6) | 5 (6) | 90 (229) | 237 (292) | 1 (6) | 5 (6) |
| **Estonia** | **2020** | 207 (188) | 106 (139) | 3 (2) | 2 (4) | 204 (185) | 109 (142) | 3 (2) | 2 (4) | 205 (185) | 109 (143) | 3 (2) | 2 (4) | 206 (185) | 108 (143) | 3 (2) | 2 (4) |
|  | **2050** | 173 (141) | 140 (187) | 4 (2) | 1 (4) | 175 (146) | 137 (182) | 4 (2) | 1 (4) | 172 (143) | 141 (185) | 4 (2) | 1 (4) | 173 (132) | 141 (196) | 4 (2) | 1 (4) |
|  | **2080** | 130 (83) | 184 (245) | 1 (2) | 4 (4) | 140 (88) | 174 (240) | 1 (1) | 4 (5) | 149 (100) | 165 (228) | 3 (1) | 2 (5) | 147 (94) | 167 (234) | 3 (1) | 2 (5) |
| **Finland** | **2020** | 243 (216) | 86 (98) | 5 (3) | 1 (2) | 241 (213) | 88 (101) | 5 (3) | 1 (2) | 237 (211) | 93 (103) | 5 (3) | 1 (2) | 237 (212) | 92 (102) | 5 (3) | 1 (2) |
|  | **2050** | 223 (181) | 107 (133) | 5 (5) | 1 (0) | 223 (181) | 107 (132) | 5 (5) | 1 (0) | 223 (179) | 107 (134) | 5 (5) | 1 (0) | 222 (182) | 108 (132) | 5 (5) | 1 (0) |
|  | **2080** | 202 (137) | 128 (177) | 5 (1) | 1 (4) | 207 (149) | 123 (165) | 5 (1) | 1 (4) | 212 (159) | 118 (155) | 5 (3) | 1 (2) | 209 (157) | 120 (157) | 5 (2) | 1 (3) |
| **France** | **2020** | 331 (224) | 556 (107) | 9 (5) | 11 (1) | 333 (224) | 553 (107) | 9 (5) | 11 (1) | 327 (222) | 560 (109) | 8 (5) | 12 (1) | 332 (224) | 555 (107) | 9 (5) | 11 (1) |
|  | **2050** | 298 (196) | 586 (135) | 7 (5) | 13 (1) | 295 (196) | 589 (135) | 8 (5) | 12 (1) | 294 (195) | 591 (136) | 8 (5) | 12 (1) | 292 (195) | 592 (136) | 8 (5) | 12 (1) |
|  | **2080** | 272 (171) | 620 (160) | 9 (5) | 11 (1) | 285 (180) | 604 (151) | 9 (5) | 11 (1) | 300 (185) | 588 (146) | 9 (5) | 11 (1) | 296 (181) | 593 (150) | 9 (5) | 11 (1) |
| **Germany** | **2020** | 206 (312) | 408 (576) | 4 (9) | 9 (11) | 200 (308) | 413 (579) | 4 (9) | 9 (11) | 200 (307) | 413 (581) | 4 (9) | 9 (11) | 199 (307) | 415 (581) | 4 (9) | 9 (11) |
|  | **2050** | 193 (281) | 422 (607) | 5 (9) | 8 (11) | 190 (280) | 425 (607) | 5 (9) | 8 (11) | 192 (275) | 422 (612) | 5 (9) | 8 (11) | 196 (279) | 418 (608) | 5 (9) | 8 (11) |
|  | **2080** | 181 (257) | 434 (635) | 6 (10) | 7 (10) | 188 (267) | 428 (623) | 5 (9) | 8 (11) | 193 (282) | 420 (607) | 4 (9) | 9 (11) | 194 (278) | 419 (611) | 5 (9) | 8 (11) |
| **Greece** | **2020** | 189 (190) | 525 (425) | 5 (4) | 11 (9) | 188 (187) | 527 (428) | 5 (4) | 11 (9) | 195 (186) | 519 (428) | 5 (4) | 11 (9) | 194 (185) | 520 (430) | 5 (4) | 11 (9) |
|  | **2050** | 261 (185) | 458 (431) | 8 (5) | 8 (8) | 267 (186) | 453 (430) | 8 (5) | 8 (8) | 257 (184) | 463 (432) | 7 (5) | 9 (8) | 267 (185) | 451 (431) | 7 (5) | 9 (8) |
|  | **2080** | 214 (178) | 506 (437) | 7 (6) | 9 (7) | 222 (188) | 498 (427) | 7 (5) | 9 (8) | 212 (190) | 505 (426) | 6 (4) | 10 (9) | 229 (188) | 490 (427) | 6 (5) | 10 (8) |
| **Hungary** | **2020** | 176 (169) | 353 (552) | 6 (6) | 9 (10) | 175 (177) | 354 (545) | 7 (6) | 8 (10) | 177 (174) | 351 (548) | 7 (7) | 8 (9) | 181 (183) | 348 (537) | 8 (7) | 7 (9) |
|  | **2050** | 148 (247) | 379 (474) | 7 (9) | 8 (7) | 151 (254) | 376 (468) | 7 (8) | 8 (8) | 171 (252) | 356 (470) | 7 (9) | 8 (7) | 177 (268) | 349 (454) | 8 (9) | 7 (7) |
|  | **2080** | 104 (207) | 425 (515) | 4 (7) | 11 (9) | 113 (206) | 415 (516) | 5 (7) | 10 (9) | 142 (202) | 384 (520) | 5 (6) | 10 (10) | 145 (210) | 382 (512) | 5 (6) | 9 (10) |
| **Ireland** | **2020** | 99 (155) | 177 (374) | 3 (6) | 1 (9) | 99 (158) | 178 (371) | 3 (6) | 1 (9) | 98 (166) | 179 (363) | 3 (6) | 1 (9) | 98 (177) | 179 (352) | 3 (7) | 1 (8) |
|  | **2050** | 81 (137) | 195 (392) | 4 (6) | 0 (9) | 83 (140) | 193 (388) | 4 (5) | 0 (10) | 82 (159) | 194 (370) | 4 (6) | 0 (9) | 80 (166) | 196 (363) | 4 (5) | 0 (10) |
|  | **2080** | 73 (93) | 203 (436) | 4 (3) | 0 (12) | 78 (107) | 199 (422) | 4 (3) | 0 (12) | 79 (134) | 195 (395) | 4 (4) | 0 (11) | 79 (140) | 196 (389) | 4 (4) | 0 (11) |
| **Italy** | **2020** | 488 (112) | 434 (165) | 12 (3) | 10 (1) | 490 (112) | 433 (164) | 12 (3) | 10 (1) | 478 (110) | 443 (167) | 12 (3) | 10 (1) | 489 (111) | 432 (166) | 13 (3) | 9 (1) |
|  | **2050** | 421 (76) | 503 (201) | 12 (4) | 9 (0) | 421 (79) | 504 (198) | 12 (4) | 10 (0) | 418 (77) | 508 (200) | 12 (4) | 10 (0) | 428 (77) | 498 (200) | 13 (4) | 9 (0) |
|  | **2080** | 347 (62) | 579 (215) | 11 (3) | 11 (1) | 362 (69) | 564 (208) | 11 (4) | 11 (0) | 370 (75) | 555 (202) | 11 (4) | 11 (0) | 370 (73) | 556 (204) | 11 (4) | 11 (0) |
| **Latvia** | **2020** | 214 (318) | 117 (609) | 2 (10) | 4 (12) | 213 (313) | 116 (613) | 2 (10) | 4 (12) | 214 (299) | 117 (628) | 2 (9) | 4 (13) | 215 (311) | 116 (616) | 2 (10) | 4 (12) |
|  | **2050** | 189 (276) | 141 (651) | 3 (10) | 3 (12) | 192 (278) | 138 (649) | 3 (10) | 3 (12) | 185 (273) | 145 (654) | 3 (10) | 3 (12) | 189 (287) | 141 (640) | 3 (10) | 3 (12) |
|  | **2080** | 143 (263) | 188 (664) | 2 (10) | 4 (12) | 157 (265) | 174 (661) | 2 (11) | 4 (11) | 169 (258) | 162 (668) | 2 (9) | 4 (13) | 169 (268) | 162 (659) | 2 (11) | 4 (11) |
| **Lithuania** | **2020** | 224 (211) | 110 (120) | 2 (2) | 4 (4) | 222 (212) | 112 (119) | 2 (2) | 5 (4) | 222 (212) | 113 (119) | 2 (2) | 5 (4) | 222 (213) | 113 (118) | 2 (2) | 5 (4) |
|  | **2050** | 167 (189) | 167 (142) | 4 (3) | 3 (3) | 177 (193) | 157 (138) | 4 (3) | 3 (3) | 175 (183) | 159 (148) | 4 (3) | 3 (3) | 168 (189) | 166 (142) | 4 (3) | 3 (3) |
|  | **2080** | 135 (141) | 201 (190) | 2 (2) | 5 (4) | 141 (149) | 195 (182) | 2 (2) | 5 (4) | 160 (165) | 175 (166) | 2 (2) | 5 (4) | 150 (167) | 185 (164) | 2 (2) | 5 (4) |
| **Luxembourg** | **2020** | 137 (220) | 190 (117) | 2 (2) | 3 (5) | 133 (222) | 194 (115) | 2 (2) | 4 (5) | 135 (220) | 194 (117) | 2 (2) | 4 (5) | 132 (220) | 193 (117) | 2 (2) | 4 (5) |
|  | **2050** | 121 (167) | 206 (170) | 3 (4) | 3 (3) | 133 (178) | 195 (159) | 3 (4) | 3 (3) | 134 (174) | 194 (163) | 3 (4) | 3 (3) | 125 (171) | 203 (166) | 3 (4) | 3 (3) |
|  | **2080** | 81 (136) | 246 (201) | 3 (2) | 3 (5) | 102 (143) | 226 (194) | 5 (2) | 1 (5) | 111 (158) | 218 (179) | 5 (2) | 1 (5) | 103 (152) | 225 (185) | 5 (2) | 1 (5) |
| **Malta** | **2020** | 98 (141) | 41 (188) | 2 (3) | 1 (3) | 100 (133) | 40 (195) | 2 (2) | 1 (4) | 99 (134) | 39 (194) | 2 (2) | 1 (4) | 95 (134) | 40 (194) | 2 (3) | 1 (3) |
|  | **2050** | 119 (119) | 43 (210) | 2 (3) | 1 (3) | 121 (131) | 43 (198) | 2 (3) | 1 (3) | 120 (131) | 44 (198) | 2 (3) | 1 (3) | 116 (125) | 45 (203) | 2 (3) | 1 (3) |
|  | **2080** | 115 (82) | 49 (247) | 1 (3) | 1 (3) | 112 (97) | 49 (231) | 2 (4) | 1 (2) | 116 (107) | 44 (222) | 2 (4) | 1 (2) | 119 (97) | 42 (232) | 2 (4) | 1 (2) |
| **Netherlands** | **2020** | 104 (125) | 251 (56) | 4 (3) | 2 (1) | 103 (124) | 252 (55) | 4 (3) | 2 (1) | 102 (125) | 252 (55) | 4 (3) | 2 (1) | 99 (123) | 255 (56) | 4 (3) | 2 (1) |
|  | **2050** | 104 (135) | 250 (46) | 5 (3) | 1 (1) | 98 (134) | 256 (47) | 5 (3) | 1 (1) | 103 (135) | 251 (47) | 5 (4) | 1 (0) | 102 (133) | 255 (48) | 5 (3) | 1 (1) |
|  | **2080** | 111 (122) | 246 (58) | 5 (3) | 1 (1) | 114 (120) | 242 (61) | 5 (3) | 1 (1) | 106 (127) | 249 (53) | 5 (3) | 1 (1) | 105 (131) | 250 (51) | 5 (3) | 1 (1) |
| **Poland** | **2020** | 264 (98) | 263 (260) | 4 (5) | 8 (1) | 263 (98) | 264 (260) | 4 (5) | 8 (1) | 270 (99) | 258 (259) | 4 (5) | 8 (1) | 267 (98) | 260 (260) | 4 (5) | 8 (1) |
|  | **2050** | 211 (105) | 317 (253) | 6 (5) | 6 (1) | 215 (99) | 312 (259) | 6 (5) | 6 (1) | 225 (106) | 303 (252) | 6 (5) | 6 (1) | 223 (102) | 304 (256) | 6 (5) | 6 (1) |
|  | **2080** | 202 (112) | 327 (246) | 5 (5) | 7 (1) | 212 (111) | 316 (247) | 5 (5) | 7 (1) | 227 (107) | 299 (251) | 5 (5) | 7 (1) | 223 (106) | 306 (252) | 5 (5) | 7 (1) |
| **Portugal** | **2020** | 202 (252) | 285 (277) | 5 (4) | 3 (8) | 199 (253) | 284 (276) | 5 (4) | 3 (8) | 207 (256) | 278 (273) | 5 (4) | 3 (8) | 212 (256) | 275 (273) | 5 (4) | 3 (8) |
|  | **2050** | 148 (207) | 340 (322) | 5 (6) | 4 (6) | 151 (213) | 335 (314) | 4 (6) | 4 (6) | 157 (219) | 331 (310) | 5 (6) | 4 (6) | 166 (221) | 322 (308) | 5 (6) | 4 (6) |
|  | **2080** | 157 (191) | 334 (338) | 5 (5) | 4 (7) | 158 (206) | 332 (323) | 5 (5) | 4 (7) | 164 (226) | 324 (303) | 5 (5) | 4 (7) | 170 (217) | 319 (312) | 5 (5) | 4 (7) |
| **Romania** | **2020** | 383 (201) | 278 (289) | 6 (6) | 9 (3) | 380 (203) | 281 (288) | 7 (6) | 8 (3) | 382 (209) | 278 (281) | 6 (6) | 9 (3) | 384 (213) | 275 (278) | 6 (6) | 9 (3) |
|  | **2050** | 288 (140) | 371 (350) | 7 (4) | 8 (5) | 291 (146) | 370 (344) | 7 (4) | 8 (5) | 292 (147) | 369 (342) | 8 (3) | 7 (6) | 306 (150) | 355 (340) | 9 (4) | 6 (5) |
|  | **2080** | 228 (152) | 433 (338) | 7 (5) | 8 (4) | 247 (151) | 412 (338) | 7 (5) | 8 (4) | 275 (155) | 386 (335) | 7 (4) | 8 (5) | 275 (166) | 386 (324) | 7 (4) | 8 (5) |
| **Slovakia** | **2020** | 324 (330) | 237 (331) | 7 (7) | 6 (8) | 324 (325) | 237 (336) | 7 (7) | 6 (8) | 327 (335) | 234 (326) | 7 (7) | 6 (8) | 328 (340) | 234 (321) | 7 (7) | 6 (8) |
|  | **2050** | 277 (210) | 286 (452) | 8 (6) | 5 (9) | 281 (212) | 282 (450) | 8 (6) | 5 (9) | 289 (221) | 271 (441) | 8 (7) | 5 (8) | 287 (225) | 276 (437) | 8 (7) | 5 (8) |
|  | **2080** | 201 (166) | 360 (495) | 6 (3) | 7 (12) | 233 (186) | 330 (475) | 7 (4) | 6 (11) | 251 (212) | 310 (450) | 7 (6) | 6 (9) | 248 (217) | 315 (445) | 7 (6) | 6 (9) |
| **Slovenia** | **2020** | 264 (269) | 349 (293) | 8 (7) | 9 (6) | 270 (271) | 343 (291) | 8 (7) | 9 (6) | 261 (267) | 352 (296) | 8 (7) | 9 (6) | 260 (266) | 355 (297) | 8 (7) | 9 (6) |
|  | **2050** | 329 (240) | 287 (323) | 11 (6) | 6 (7) | 335 (244) | 281 (318) | 12 (6) | 5 (7) | 326 (252) | 290 (310) | 11 (6) | 6 (7) | 324 (246) | 292 (316) | 11 (6) | 6 (7) |
|  | **2080** | 254 (151) | 364 (410) | 10 (4) | 7 (9) | 284 (178) | 334 (385) | 10 (5) | 7 (8) | 279 (207) | 339 (356) | 10 (5) | 7 (8) | 277 (209) | 341 (354) | 10 (6) | 7 (7) |
| **Spain** | **2020** | 332 (234) | 499 (382) | 5 (8) | 8 (9) | 335 (234) | 495 (382) | 5 (8) | 8 (9) | 331 (228) | 500 (387) | 5 (8) | 8 (9) | 342 (229) | 488 (386) | 5 (8) | 8 (9) |
|  | **2050** | 276 (292) | 555 (326) | 6 (10) | 7 (7) | 287 (299) | 544 (319) | 6 (10) | 7 (7) | 274 (287) | 557 (331) | 6 (10) | 7 (7) | 283 (289) | 548 (328) | 6 (10) | 7 (7) |
|  | **2080** | 262 (219) | 571 (399) | 7 (10) | 6 (7) | 276 (245) | 555 (373) | 7 (10) | 6 (7) | 280 (241) | 550 (376) | 7 (9) | 6 (8) | 286 (247) | 545 (370) | 7 (9) | 6 (8) |
| **Sweden** | **2020** | 278 (259) | 147 (573) | 5 (4) | 4 (9) | 279 (259) | 147 (573) | 5 (4) | 4 (9) | 281 (263) | 146 (569) | 5 (5) | 4 (8) | 282 (268) | 146 (564) | 5 (5) | 4 (8) |
|  | **2050** | 267 (217) | 160 (614) | 6 (5) | 3 (8) | 269 (212) | 159 (619) | 6 (5) | 3 (8) | 272 (219) | 154 (613) | 7 (5) | 2 (8) | 265 (221) | 160 (610) | 6 (5) | 3 (8) |
|  | **2080** | 227 (232) | 202 (600) | 5 (7) | 4 (6) | 239 (228) | 189 (604) | 5 (6) | 4 (7) | 253 (243) | 175 (588) | 5 (6) | 4 (7) | 250 (237) | 178 (594) | 5 (6) | 4 (7) |
| **UK** | **2020** | 186 (270) | 226 (159) | 4 (5) | 1 (4) | 183 (269) | 229 (160) | 4 (5) | 1 (4) | 187 (270) | 225 (159) | 4 (5) | 1 (4) | 187 (268) | 225 (161) | 4 (5) | 1 (4) |
|  | **2050** | 172 (247) | 240 (181) | 4 (6) | 1 (3) | 178 (247) | 233 (181) | 4 (6) | 1 (3) | 177 (249) | 234 (180) | 4 (7) | 1 (2) | 174 (248) | 237 (181) | 4 (6) | 1 (3) |
|  | **2080** | 149 (208) | 263 (221) | 4 (5) | 1 (4) | 158 (224) | 254 (204) | 4 (5) | 1 (4) | 174 (230) | 238 (199) | 4 (5) | 1 (4) | 169 (229) | 242 (200) | 4 (5) | 1 (4) |

**Table S4a** – Variability in the numbers of species projected to gain (win) and lose (los) climatic suitability in European protected areas. Values are provided for different combinations of emission scenarios and time periods and using projections made with ensembles from seven bioclimatic modelling techniques and three general circulation models. Results for Red-List species (RL species) and for the complete pool of vertebrate and plant species considered (all). For each year, the winner and loser values in the first line refers to minimum and maximum values projected from the ensemble; second line refers to median value; and third line to standard deviation of values from the ensemble of forecasts.

|  | **A1FI** | | | | **A2** | | | | **B1** | | | | **B2** | | | |
| --- | --- | --- | --- | --- | --- | --- | --- | --- | --- | --- | --- | --- | --- | --- | --- | --- |
|  | **RL species** | | **all** | | **RL species** | | **all** | | **RL species** | | **all** | | **RL species** | | **all** | |
|  | **win** | **los** | **win** | **los** | **win** | **los** | **win** | **los** | **win** | **los** | **win** | **los** | **win** | **los** | **win** | **los** |
|  |  |  |  |  |  |  |  |  |  |  |  |  |  |  |  |  |
| ***Amphibians*** | |  |  |  |  |  |  |  |  |  |  |  |  |  |  |  |
| **2020** | 2;8 | 0;6 | 9;41 | 1;33 | 2;8 | 0;6 | 8;41 | 1;34 | 2;8 | 0;6 | 8;41 | 1;34 | 2;8 | 0;6 | 7;41 | 1;35 |
|  | 6 (2.05) | 2 (2.05) | 30  (8.8) | 12 (8.91) | 6 (2.12) | 2 (2.12) | 31  (9.39) | 11 (9.49) | 6 (1.93) | 2 (1.93) | 30  (9.01) | 12 (9.11) | 7 (1.88) | 1 (1.88) | 32  (9.53) | 10 (9.63) |
| **2050** | 1;8 | 0;7 | 12;41 | 1;30 | 1;8 | 0;7 | 14;42 | 0;28 | 1;8 | 0;7 | 11;41 | 1;31 | 2;8 | 0;6 | 13;42 | 0;29 |
|  | 5 (1.94) | 3 (1.94) | 28  (7.76) | 14 (7.82) | 5 (1.86) | 3 (1.86) | 27  (7.4) | 15 (7.47) | 5 (1.94) | 3 (1.94) | 28  (7.69) | 14 (7.75) | 5 (1.88) | 3 (1.88) | 28  (6.93) | 14 (7.01) |
| **2080** | 1;8 | 0;7 | 9;40 | 2;33 | 2;8 | 0;6 | 12;41 | 1;30 | 1;8 | 0;7 | 11;41 | 1;31 | 2;8 | 0;6 | 16;42 | 0;26 |
|  | 4 (2.18) | 4 (2.18) | 24  (8.41) | 18 (8.54) | 4 (2.13) | 4 (2.13) | 24  (7.84) | 18 (7.95) | 4 (2.16) | 4 (2.16) | 25  (7.45) | 17 (7.56) | 4 (2.09) | 4 (2.09) | 25  (6.95) | 17 (7.09) |
|  |  |  |  |  |  |  |  |  |  |  |  |  |  |  |  |  |
| ***Reptiles*** | |  |  |  |  |  |  |  |  |  |  |  |  |  |  |  |
| **2020** | 1;7 | 0;6 | 35;63 | 1;29 | 1;7 | 0;6 | 35;63 | 1;29 | 1;6 | 1;6 | 35;63 | 1;29 | 1;6 | 1;6 | 34;63 | 1;30 |
|  | 5 (1.53) | 2 (1.53) | 49  (8.87) | 15 (8.87) | 5 (1.51) | 2 (1.51) | 50  (8.39) | 14 (8.39) | 5 (1.66) | 2 (1.66) | 49  (8.81) | 15 (8.81) | 5 (1.47) | 2 (1.47) | 50  (8.48) | 14 (8.48) |
| **2050** | 2;5 | 2;5 | 30;53 | 11;33 | 2;5 | 2;5 | 31;54 | 10;33 | 1;5 | 2;6 | 27;55 | 9;37 | 2;5 | 2;5 | 31;55 | 9;33 |
|  | 3 (0.93) | 4 (0.93) | 44  (6.77) | 20 (6.68) | 3 (0.89) | 4 (0.89) | 43  (6.82) | 21 (6.82) | 3 (1.05) | 4 (1.05) | 45  (7.56) | 19 (7.55) | 3 (0.94) | 4 (0.94) | 42  (6.92) | 22 (6.92) |
| **2080** | 1;6 | 1;6 | 25;57 | 7;39 | 1;6 | 1;6 | 28;59 | 5;36 | 2;6 | 1;5 | 30;58 | 6;34 | 1;7 | 0;6 | 31;60 | 4;33 |
|  | 3 (1.23) | 4 (1.23) | 42  (9.09) | 22 (9.09) | 3 (1.54) | 4 (1.54) | 43  (8.76) | 21 (8.76) | 4 (1.34) | 3 (1.34) | 45  (7.75) | 19 (7.75) | 4  (1.6) | 3 (1.6) | 45  (7.72) | 19 (7.72) |
|  |  |  |  |  |  |  |  |  |  |  |  |  |  |  |  |  |
| ***Birds*** | |  |  |  |  |  |  |  |  |  |  |  |  |  |  |  |
| **2020** | 4;8 | 6;9 | 111;191 | 134;205 | 3;8 | 6;10 | 112;192 | 133;204 | 4;8 | 6;9 | 111;192 | 133;205 | 3;8 | 6;10 | 111;193 | 132;205 |
|  | 5  (1.2) | 9 (0.98) | 144 (25.03) | 172 (23.51) | 5 (1.24) | 9 (1.03) | 142 (24.32) | 174 (22.51) | 5 (1.28) | 9 (1.08) | 140 (25.11) | 179 (23.42) | 5 (1.33) | 9 (1.12) | 143 (25.67) | 176 (23.9) |
| **2050** | 2;9 | 5;11 | 109;206 | 110;208 | 2;9 | 5;11 | 108;190 | 132;209 | 2;9 | 5;11 | 103;189 | 133;214 | 2;9 | 5;11 | 104;193 | 130;213 |
|  | 7 (1.99) | 7 (1.61) | 160 (23.4) | 168 (22.58) | 7 (2.18) | 7 (1.79) | 162 (22.59) | 165 (20.81) | 7 (2.01) | 7 (1.65) | 157 (24.65) | 162 (22.92) | 7  (2.1) | 7 (1.71) | 162 (24.48) | 166 (22.68) |
| **2080** | 4;8 | 6;10 | 121;190 | 128;213 | 4;9 | 5;10 | 120;204 | 112;202 | 3;9 | 5;11 | 125;220 | 96;200 | 4;9 | 5;10 | 124;211 | 105;202 |
|  | 6 (1.38) | 8 (1.24) | 162 (24.33) | 164  (25) | 6 (1.48) | 8 (1.33) | 163 (26.03) | 159 (26.93) | 5 (1.73) | 8 (1.54) | 168 (28.72) | 158 (29.89) | 5  (1.5) | 8 (1.34) | 166 (27.39) | 159 (28.21) |
|  |  |  |  |  |  |  |  |  |  |  |  |  |  |  |  |  |
| ***Mammals*** | |  |  |  |  |  |  |  |  |  |  |  |  |  |  |  |
| **2020** | 7;13 | 4;10 | 54;103 | 36;84 | 7;13 | 4;10 | 56;103 | 36;82 | 6;13 | 4;11 | 55;101 | 38;83 | 6;13 | 4;11 | 55;103 | 36;83 |
|  | 9 (1.98) | 8 (1.98) | 71 (14.85) | 68 (14.96) | 9 (1.93) | 8 (1.93) | 72 (14.64) | 67 (14.73) | 9 (2.14) | 8 (2.14) | 72 (14.65) | 67 (14.74) | 9 (1.83) | 8 (1.83) | 72 (14.73) | 67 (14.79) |
| **2050** | 5;12 | 5;12 | 40;104 | 35;99 | 6;12 | 5;11 | 43;105 | 34;96 | 4;11 | 6;13 | 33;102 | 37;106 | 6;13 | 4;11 | 42;106 | 33;97 |
|  | 8 (1.99) | 9 (1.99) | 73 (17.54) | 66 (17.61) | 8 (1.83) | 9 (1.83) | 70 (16.1) | 69 (16.15) | 8 (2.25) | 9 (2.25) | 71 (19.05) | 68 (19.12) | 8 (2.06) | 9 (2.06) | 70 (17.89) | 69 (17.95) |
| **2080** | 4;13 | 4;13 | 33;102 | 37;106 | 5;13 | 4;12 | 38;103 | 36;101 | 5;13 | 4;12 | 35;102 | 37;104 | 5;13 | 4;12 | 38;102 | 37;101 |
|  | 8 (2.31) | 9 (2.31) | 64 (18.58) | 75 (18.84) | 9 (2.12) | 8 (2.12) | 72 (18.26) | 66 (18.51) | 8 (1.87) | 9 (1.87) | 73 (19.13) | 64 (19.49) | 9 (2.01) | 8 (2.01) | 74 (17.74) | 65 (18.06) |
|  |  |  |  |  |  |  |  |  |  |  |  |  |  |  |  |  |
| ***Plants*** | |  |  |  |  |  |  |  |  |  |  |  |  |  |  |  |
| **2020** | 0;3 | 0;3 | 527;1012 | 282;778 | 0;3 | 0;3 | 518;1032 | 262;787 | 0;3 | 0;3 | 514;1030 | 267;791 | 0;3 | 0;3 | 516;1036 | 258;789 |
|  | 1 (0.85) | 1 (1.03) | 630 (163.62) | 675 (167.86) | 1 (0.86) | 2 (1.03) | 635 (165.28) | 670 (169.45) | 1 (0.83) | 2 (1.03) | 630 (172.47) | 674 (176.39) | 1 (0.86) | 1 (1.03) | 635 (176.56) | 670 (181.13) |
| **2050** | 0;3 | 0;3 | 495;880 | 417;809 | 0;3 | 0;3 | 493;893 | 403;812 | 1;3 | 0;2 | 487;878 | 418;817 | 0;3 | 0;3 | 481;900 | 396;824 |
|  | 2 (0.89) | 1  (1) | 658 (108.5) | 647 (111.16) | 2 (1.04) | 1 (1.14) | 649 (107.95) | 656 (111.03) | 2 (0.81) | 1 (0.92) | 659 (116.19) | 646 (119.33) | 2 (0.96) | 1 (1.05) | 654 (116.5) | 651 (119.79) |
| **2080** | 0;3 | 0;3 | 373;911 | 386;932 | 0;3 | 0;3 | 445;933 | 363;860 | 0;2 | 0;3 | 460;916 | 381;845 | 0;3 | 0;3 | 459;937 | 359;846 |
|  | 1 (0.8) | 2 (0.98) | 521 (149.95) | 784 (154.01) | 1 (0.85) | 2 (1.03) | 559 (140.84) | 746 (144.69) | 1 (0.77) | 2 (1.00) | 571 (151.87) | 734 (155.08) | 1 (0.83) | 2 (1.02) | 566 (144.03) | 739 (147.74) |
|  |  |  |  |  |  |  |  |  |  |  |  |  |  |  |  |  |

**Table S4b** – Variability in the numbers of species projected to gain (win) and lose (los) climatic suitability in the Natura 2000 areas. Values are provided for different combinations of emission scenarios and time periods and using projections made with ensembles from seven bioclimatic modelling techniques and three general circulation models. Results for Habitat-directive species (HD species) and for the complete pool of vertebrate and plant species considered (all). For each year, the winner and loser values in the first line refers to minimum and maximum values projected from the ensemble; second line refers to median value; and third line to standard deviation of values from the ensemble of forecasts.

|  | **A1FI** | | | | **A2** | | | | **B1** | | | | **B2** | | | |
| --- | --- | --- | --- | --- | --- | --- | --- | --- | --- | --- | --- | --- | --- | --- | --- | --- |
|  | **HD species** | | **all** | | **HD species** | | **all** | | **HD species** | | **all** | | **HD species** | | **all** | |
|  | **win** | **los** | **win** | **los** | **win** | **los** | **win** | **los** | **win** | **los** | **win** | **los** | **win** | **los** | **win** | **los** |
|  |  |  |  |  |  |  |  |  |  |  |  |  |  |  |  |  |
| ***Amphibians*** | |  |  |  |  |  |  |  |  |  |  |  |  |  |  |  |
| 2020 | 7;31 | 1;25 | 9;41 | 1;33 | 5;31 | 1;27 | 7;41 | 1;35 | 5;31 | 1;27 | 7;41 | 1;35 | 6;31 | 1;26 | 8;41 | 1;34 |
|  | 21 (6.47) | 11 (6.59) | 28 (8.89) | 14  (9.01) | 21  (6.7) | 11 (6.82) | 28  (9.25) | 14  (9.37) | 21 (6.72) | 11 (6.84) | 28  (9.09) | 14  (9.2) | 22 (6.79) | 10 (6.91) | 28  (9.2) | 14  (9.31) |
| 2050 | 2;30 | 2;30 | 3;40 | 2;39 | 2;32 | 0;30 | 2;42 | 0;40 | 3;29 | 3;29 | 3;39 | 3;39 | 2;32 | 0;30 | 2;42 | 0;40 |
|  | 18 (6.54) | 14 (6.62) | 21 (8.89) | 21  (8.97) | 18 (6.95) | 14 (7.02) | 22  (9.29) | 20  (9.36) | 18 (7.17) | 14 (7.24) | 23  (9.75) | 19  (9.83) | 19 (7.03) | 13 (7.11) | 23  (9.37) | 19  (9.44) |
| 2080 | 0;30 | 2;32 | 0;39 | 3;42 | 1;31 | 1;31 | 2;41 | 1;40 | 5;30 | 2;27 | 6;39 | 3;36 | 6;32 | 0;26 | 7;42 | 0;35 |
|  | 14 (8.07) | 18 (8.16) | 17 (10.83) | 25 (10.93) | 15 (7.65) | 17 (7.75) | 18 (10.43) | 24 (10.53) | 16  (6.8) | 16  (6.9) | 19  (9.56) | 23  (9.66) | 17 (6.33) | 15 (6.42) | 21  (8.97) | 21  (9.08) |
|  |  |  |  |  |  |  |  |  |  |  |  |  |  |  |  |  |
| ***Reptiles*** | |  |  |  |  |  |  |  |  |  |  |  |  |  |  |  |
| 2020 | 15;35 | 1;21 | 25;62 | 2;39 | 18;35 | 1;18 | 25;62 | 2;39 | 14;35 | 1;22 | 26;63 | 1;38 | 14;35 | 1;22 | 26;63 | 1;38 |
|  | 22 (5.96) | 14 (5.81) | 42 (11.36) | 22 (11.28) | 24 (5.52) | 12 (5.39) | 42 (11.21) | 22 (11.15) | 23 (6.24) | 13 (6.15) | 42 (11.76) | 22 (11.72) | 25  (5.8) | 11 (5.67) | 44 (11.01) | 20 (10.94) |
| 2050 | 4;32 | 4;32 | 7;52 | 12;57 | 5;32 | 4;31 | 8;54 | 10;56 | 4;32 | 4;32 | 7;54 | 10;57 | 5;32 | 4;31 | 8;54 | 10;56 |
|  | 27 (8.03) | 9  (8.03) | 40 (13.12) | 24 (13.12) | 27 (8.29) | 9  (8.29) | 39 (13.54) | 25 (13.54) | 27  (8.2) | 9  (8.21) | 41 (13.46) | 23 (13.47) | 27 (8.24) | 9  (8.24) | 40 (13.65) | 24 (13.65) |
| 2080 | 1;35 | 1;35 | 1;57 | 7;63 | 2;35 | 1;34 | 2;58 | 6;62 | 4;35 | 1;32 | 8;58 | 6;56 | 3;35 | 1;33 | 6;58 | 6;58 |
|  | 23 (9.91) | 13 (9.91) | 35 (15.64) | 29 (15.64) | 24 (9.53) | 12 (9.53) | 36 (15.02) | 28 (15.02) | 24 (8.54) | 12 (8.54) | 38  (14) | 26  (14) | 25 (8.96) | 11 (8.96) | 40 (14.24) | 24 (14.24) |
|  |  |  |  |  |  |  |  |  |  |  |  |  |  |  |  |  |
| ***Birds*** | |  |  |  |  |  |  |  |  |  |  |  |  |  |  |  |
| 2020 | 54;94 | 72;111 | 109;190 | 136;217 | 56;97 | 69;109 | 112;192 | 134;214 | 55;97 | 69;111 | 110;196 | 130;214 | 54;97 | 69;109 | 110;195 | 131;213 |
|  | 76 (14.8) | 87 (14.44) | 145 (27.16) | 174 (26.33) | 76 (14.15) | 87 (13.7) | 144 (26.61) | 176 (25.71) | 72 (14.82) | 91 (14.39) | 143 (28.48) | 179 (27.58) | 72 (15.01) | 91 (14.41) | 142 (28.15) | 179 (27.07) |
| 2050 | 57;96 | 69;104 | 109;183 | 138;210 | 58;96 | 69;103 | 108;184 | 137;211 | 57;96 | 69;104 | 99;182 | 139;220 | 58;97 | 68;103 | 100;183 | 138;219 |
|  | 73 (12.28) | 89 (11.09) | 141 (22.18) | 180 (21.1) | 73 (12.43) | 91 (11.12) | 143 (23.3) | 182 (21.87) | 73 (12.55) | 92 (11.28) | 141 (24.97) | 182 (23.54) | 71 (13.07) | 93 (11.86) | 138 (25.45) | 186 (24.03) |
| 2080 | 47;95 | 70;114 | 107;186 | 140;214 | 47;96 | 69;114 | 112;188 | 138;210 | 47;98 | 67;114 | 111;190 | 136;211 | 46;96 | 69;115 | 111;189 | 137;212 |
|  | 68 (16.31) | 96 (15.22) | 128 (26.6) | 194 (25.76) | 68 (15.51) | 93 (14.41) | 130 (25.94) | 190 (24.93) | 78 (16.9) | 87 (16.16) | 150 (27.38) | 175 (26.91) | 76 (16.13) | 86 (15.29) | 143 (26.7) | 175 (25.94) |
|  |  |  |  |  |  |  |  |  |  |  |  |  |  |  |  |  |
| ***Mammals*** | |  |  |  |  |  |  |  |  |  |  |  |  |  |  |  |
| 2020 | 9;44 | 5;40 | 39;97 | 40;99 | 9;44 | 5;40 | 46;98 | 39;92 | 9;44 | 5;40 | 43;101 | 37;95 | 8;44 | 5;41 | 42;101 | 37;96 |
|  | 31 (10.51) | 19 (10.44) | 70 (16.13) | 69 (16.25) | 31 (10.17) | 19 (10.11) | 69 (15.63) | 70 (15.72) | 32 (10.23) | 18 (10.19) | 71 (15.51) | 68 (15.59) | 31 (10.35) | 19 (10.28) | 70 (16.08) | 69 (16.03) |
| 2050 | 4;43 | 7;45 | 29;101 | 38;109 | 5;43 | 7;44 | 32;100 | 39;106 | 3;42 | 8;46 | 30;100 | 39;108 | 3;45 | 5;46 | 38;104 | 35;99 |
|  | 22 (10.27) | 27 (10.09) | 58 (19.52) | 81 (19.35) | 23 (10.2) | 26 (10.09) | 55 (19.6) | 83 (19.45) | 25 (10.57) | 24 (10.43) | 63 (20.45) | 75 (20.25) | 26 (10.28) | 24 (10.12) | 61 (19.17) | 77 (18.96) |
| 2080 | 3;43 | 7;46 | 14;102 | 37;123 | 4;43 | 7;45 | 24;101 | 38;114 | 10;44 | 6;39 | 25;103 | 36;113 | 8;43 | 7;41 | 31;101 | 38;107 |
|  | 18 (10.76) | 32 (10.63) | 43 (23.32) | 95  (23.2) | 17 (10.5) | 32 (10.35) | 44 (21.37) | 94 (21.28) | 20  (9.7) | 29 (9.55) | 53 (21.07) | 86 (21.07) | 21 (9.61) | 29 (9.44) | 52 (20.12) | 87 (20.06) |
|  |  |  |  |  |  |  |  |  |  |  |  |  |  |  |  |  |
| ***Plants*** | |  |  |  |  |  |  |  |  |  |  |  |  |  |  |  |
| 2020 | 7;24 | 4;21 | 471;998 | 298;833 | 8;24 | 4;20 | 460;1008 | 289;844 | 6;25 | 3;22 | 453;1002 | 294;850 | 6;24 | 4;22 | 448;1015 | 280;856 |
|  | 13 (4.92) | 15 (4.86) | 577 (181.34) | 727 (185.13) | 13 (4.82) | 15 (4.76) | 575 (183.85) | 729 (187.64) | 13 (5.15) | 15 (5.08) | 565 (183.91) | 739 (187.79) | 13 (5.13) | 15 (5.07) | 565 (188.67) | 739 (192.67) |
| 2050 | 3;24 | 4;25 | 280;848 | 449;1024 | 4;24 | 4;24 | 284;860 | 437;1020 | 4;24 | 4;24 | 271;844 | 453;1033 | 4;24 | 4;24 | 267;874 | 423;1037 |
|  | 15 (5.84) | 13 (5.83) | 543 (159.47) | 762 (162.35) | 15 (6.04) | 13 (6.03) | 554 (162.21) | 751 (165.06) | 15 (5.88) | 13 (5.88) | 582 (169.36) | 723 (172.39) | 15 (5.75) | 13 (5.72) | 568 (172.1) | 737 (174.93) |
| 2080 | 0;24 | 4;28 | 80;897 | 400;1224 | 0;23 | 5;28 | 161;901 | 396;1143 | 1;24 | 4;27 | 246;895 | 401;1058 | 0;24 | 4;28 | 223;921 | 376;1081 |
|  | 10 (6.32) | 17 (6.27) | 350 (215.55) | 949 (219.22) | 11 (6.28) | 17 (6.23) | 408 (202.28) | 896 (205.94) | 12 (6.18) | 16 (6.15) | 415 (191.94) | 885 (195.83) | 12 (6.04) | 15 (6.02) | 417 (196.48) | 886 (200.28) |
|  |  |  |  |  |  |  |  |  |  |  |  |  |  |  |  |  |
