# Supplementary material for: Climate change threatens European conservation areas
Source: Ecol Lett. 2011 May;14(5):484–92. doi: 10.1111/j.1461-0248.2011.01610.x (PMC3116148; doi:10.1111/j.1461-0248.2011.01610.x)
Supplement: Supplementary file 2 [file ele0014-0484-SD2.doc]

**Table S5 -** Species projected win and lose climate suitability in in (in) and outside (out) protected areas (PA) and Natura 2000 areas (RN), for 2020, 2050 and 2080 under different emission scenarios. W stands for projected winners, whereas L stands for projected loser species.
